# Agro-morphological and molecular diversity in different maturity groups of Indian cauliflower *(Brassica oleracea var*. *botrytis L*.*)*

**DOI:** 10.1371/journal.pone.0260246

**Published:** 2021-12-10

**Authors:** K. N. Rakshita, Shrawan Singh, Veerendra Kumar Verma, Brij Bihari Sharma, Navinder Saini, Mir Asif Iquebal, Akanksha Sharma, Shyam Sunder Dey, T. K. Behera

**Affiliations:** 1 Division of Vegetable Science, ICAR-Indian Agricultural Research Institute, New Delhi, India; 2 ICAR Research Complex for North Eastern Hill Region, Barapani, Meghalaya, India; 3 Division of Genetics, ICAR-Indian Agricultural Research Institute, New Delhi, India; 4 ICAR-Indian Agricultural Statistical Research Institute, New Delhi, India; Chungnam National University, REPUBLIC OF KOREA

## Abstract

The present study analysed the molecular and agro-morphological diversity in a set of 92 diverse cauliflower genotypes and two each of cabbage and broccoli. Field evaluation of the genotypes was done in randomized block design (RBD) at two locations (*i*.*e*. IARI, New Delhi and ICAR-RC-NEH Region, Barapani) during *Rabi*2019-20. Genotypes showed variation for all the eight observed traits at both locations and, the differences in early and snowball groups were distinct. Pusa Meghna, DC-33-8, Pusa Kartiki and CC-14 were earliest for curd initiation. Genotypes showed higher values for curd traits at Delhi. Molecular diversity was detected with 90 polymorphic simple sequence repeats (SSR). Number of alleles ranged from 1 to 9 with mean value of 2.16 and the highest polymorphic information content (PIC) value was observed for primer BoGMS0742 (0.68) with a mean value of 0.18. Cluster analysis using agro-morphological traits substantiated classification of the genotypes for maturity groups. However, SSR analysis revealed four clusters and with a composite pattern of genotype distribution. STRUCTURE analysis also supported the admixture and four subpopulations. The studyindicates for introgression of genetic fragments across the maturity groups, thereby, potential for use in further genetic improvement and heterosis breeding.

## Introduction

Cauliflower (*Brassica oleracea* var. *botrytis* L., 2n = 2X = 18) is one of the most important vegetable crops grown worldwide. The genus *Brassica* comprises of around 40 different species including *Brassica oleracea*. The *B*. *oleracea* represents the popular group of ‘Cole’ crops or ‘Cole’ vegetables. Cauliflower is one of the important crops of this group and being grown across the world on 1.42 million ha area having the annual production of 26.90 million tonnes. Of this, China (40.5%) and India (33.2%) holds major share [1]. Cauliflower contains glucosinolates which are responsible for its health protective properties and sensory attributes such as pungency, aroma and flavour.

Economic portion of cauliflower is a prefloral fleshy apical meristem commonly known as ‘curd’ [2] which is eaten as vegetable or pickle or various regional culinary recipes. The curd contributes nearly 45% of the gross plant weight [3]. Cauliflower is a thermo-sensitive crop and temperature plays key role in the regulation of curd initiation and development through a group of major genes and modifiers [4]. The cauliflower was originated in Mediterranean region and introduced in different parts of the world by traders and botanists. Genetic changes took place in introduced genotypes for adaptive traits such as plant types, curding traits and flowering behaviour which could help in evolution of eight regional morphotypes of cauliflower [5]. These groups are being recognised as Italians or Original (Mediterranean), Cornish (England), Northerns (England), Roscoff (France), Angers (France), Erfurt and Snowball (Germany and Netherlands), Indian cauliflower (Northern India). Further, cauliflowers also classified according to their phylogeny as Italian, North-West European biennials, North European annuals, Asian and Australian highlighting the significance of regional factors and growing habit [6].

Cauliflower is an introduced crop in India and has been categorised into two broad groups namely (i) European (late or snowball) and (ii) India (tropical) types depending upon their temperature requirement for curding and reproductive phases. Typical Indian cauliflower (group1a & 1b and group 2) forms curd at higher temperature (16–27°C) than snowball group (10–16°C) and, an intermediate group designated as mid-late (group-3) requires 12 to 16°C for curd initiation and development [5]. Further, Indian type cauliflower flower and set seeds profusely in northern plains during winter season while snowball cauliflower does not bolt or set seeds in plains due to its prolonged low temperature requirement. Precisely, the Indian cauliflower is grouped into early (group-1), mid-early (group-2) and mid-late (group-3) on the basis of its specific temperature requirement for curd initiation and development as 20–27°C, 16–20°C and 12–16°C, respectively [7]. Deviation from the demarcated range of temperature leads to loss in yield and quality besides occurrence of various disorders like bracting, yellowing and loose curds (at higher temperature) and buttoning, fuzziness, riciness and pink colouration (at lower temperature) [8].

The tropical type germplasm of Indian cauliflower have strong phenotypic affinity with the European Cornish type for vigorous plant type, open growth habit, long stalk and leaves, loose, irregular shaped cream and yellow curds and strong curd flavour [5]. However, few leaf and curd traits such as presence of protective jacket leaves and good curds are also found to be similar with Roscoff and Italian cauliflower [9] which was due to planned crossing programmes. This, intentional crossing attempts created new set of germplasm in Indian cauliflower having desirable curd features and semi-erect plant stature [10]. This reflects well in its present-day cultivars which have enhanced curd size, white colour and strong compactness [8, 11]. Further, lot of exotic germplasm and locally bred inter-group progenies diversified the Indian cauliflower germplasm for use in heterosis and stress resistance breeding [12]. The shift in behaviour of the genetically governed adaptive/consumer traits was due to changes in combination of major and minor genes during the evolution of new genotypes. However, researchers reported good extent of diversity using morphological traits in snowball cauliflower [13–15] and different maturity groups of tropical cauliflower groups such as early [16] and mid group [17] but these traits are sensitive to environmental conditions, therefore, their observations are not adequate the level of variation in the genotypes for breeding use. Further, these studied were done in a specific location and with limited set of germplasm, and none of them attempted with a composite set of genotypes from all the maturity groups at diverse locations. Notably, understanding the level of diversity at molecular level and corroborate this information with prominent agro-morphological traits will be of breeders’ immediate use. Since, the polymorphism revealed among the genotypes by DNA markers is independent of environmental factors. For molecular diversity analysis, simple sequence repeats (SSR) were of great use, since they are robust, reliable and codominant DNA markers [18]. They are abundant in *Brassica oleracea* cytodeme and its related species [19] with high extent of cross-transferability in *Brassica* group [20]. These markers have been used in diversity analysis in different pools of *Brassica oleracea* [21–23] and also in linkage studies [24]. Ram et al. [25] reported effectiveness of SSRs in diversity analysis and genotype identification of snowball cauliflower. Therefore, the present study was planned to assess the molecular diversity using SSR markers and also observe the agro-morphological variation in the genotypes at two diverse locations.

## Materials and methods

### Plant material and growing environment

The experiment material comprised of total ninety-six (96) genotypes out of which 92 of cauliflower, 2 of cabbage and 2 of broccoli. Cauliflower genotypes were from the core set of germplasm representative of all the four maturity groups namely early (37), mid-early (25), mid-late (15) and late/snowball (13) groups. The genotypes of first three groups are developed and maintained by IARI, New Delhi while snowball group genotypes were obtained from IARI Regional Station, Katrain, Himachal Pradesh for use a reference, India. Besides, two specialty type cauliflowers ‘GPMT-1’ (green mustard type leaves) and ‘Orange type’ (orange curd) alongwith two each of Broccoli (‘DC-Brocco-13’ and ‘Delhi Purple Broccoli-1’) and tropical Cabbage (‘PA-1’ and ‘PA-2’) were also included in molecular analysis. ‘Orange type’ cauliflower genotype was not shared with Barapani centre due to lack of permission. Seedlings were raised on nursery beds transplanted (35 days old) at 60 × 45 cm (no. of plants per plot = 30) in complete randomized block design (RBD) with three replications at IARI, New Delhi (28°35’ N, 77° 12’ E, 228.6 m above mean sea level) and ICAR Research Complex for NEH Region, Barapani, Meghalaya (25°41’ N, 91°55’ E, 960 m above mean sea level) during 2019–20, under plain and mid-hill conditions, respectively. Delhi location had wider range of temperature (3–36°C) than Barapani location (5–27°C). Similarly, the soil pH of Delhi site was high (6.8) than Barapani (5.2). Standard crop practices were followed for the crop at both locations [7]. Days to 50% curd initiation (DCI), days to 50% curd maturity (DCH), number of leaves/plants, gross plant weight (g), curd traits namely curd length or polar diameter (cm), curd width or equatorial diameter (cm), marketable curd weight (g) and net curd weight (g) were recorded from five random plants in each plot. The curd traits were observed as per the procedure described by [26, 27].

### DNA extraction and SSR analysis

Genomic DNA was extracted from the fresh leaf samples collected from field grown healthy young plants at Delhi site by using standard CTAB protocol [28]. Appropriate quantification of DNA was done by running on 0.8% agarose gel. Additionally, quality and accurate quantity of the genomic DNA was also analysed by Nanodrop spectrophotometer (Eppendorf) and diluted with TE buffer to yield a working DNA having appropriated concentration of 25-30ng/μl.

A set of 100 primers from *Brassica* group were screened for polymerase chain reaction (PCR) amplification. The sequence information of 90 SSRs which generated polymorphic amplicons is given in S1 Table. All the SSRs were amplified by PCR in 10 μl volume having 50 ng genomic DNA, 1.0 U *Taq*DNA polymerase (Hi media Laboratories, Mumbai, India), 1×PCR assay buffer with 1.5 mM MgCl_2_, 10 pmol of each primer (forward and reverse) and 100 μM of dNTPs mix (Thermo Scientific). All the primers were amplified using touchdown PCR in an Eppendorf Master cycler using the following cycling programme: initial denaturation at 94°C for 5 min followed by 30 cycles of denaturation at 94°C for 1 min; primer annealing at 55–65°C for 1 min (varied with primer); primer extension at 72°C for 2 min and final extension at 72°C for 10 min. The programme was made to retain the samples at 4°C until they were collected and stored at -20°C.

### Electrophoresis and fragment detection

The amplified PCR products were mixed with 1 μl of 1X loading dye (Bromophenol blue) and resolved on 3% agarose gels in 1X TAE buffer and stained with ethidium bromide. The bands were visualised under UV light in a gel documentation unit (Alpha Imager, Cell biosciences, Santa Clara, CA).

The scoring of amplicons was done manually using a reference of 50bp ladder. The data matrix was made using amplicon size. Power Marker v3.25 software was used for analysis of polymorphism information content (PIC), major allele frequency and cluster analysis [29]. Similarity index was calculated using Nei’s formula [30]. The UPGMA (unweighted pair group method with arithmetic mean) of the NTSYS software version 2.02i [31] was used to generate the corresponding dendrogram. Population structure analysis was done by STRUCTURE version 2.3.4. Models were tested for K-values ranging from 2 to 10 with 10 independent runs each and 100,000 Markov chain Monte Carlo (MCMC) iterations. The most likely number of clusters was chosen by plotting the Ln P(D) values against ΔK values with the best K-value selected according to the Evanno test [32] using Structure Harvester (http://taylor0.biology.ucla.edu/structureHarvester/).

### Statistical analysis

The data from agro-morphological traits were recorded and subjected to analysis of variance (ANOVA) using online OPSTAT software [33] (http://14.139.232.166/opstat/). Mean and standard deviation for the observations was calculated using Microsoft Excel 2019. DARwin 6 software was used for diversity analysis and generating dendrogram for cauliflower genotypes.

## Results

### Agro-morphological diversity

**Fig 1:**
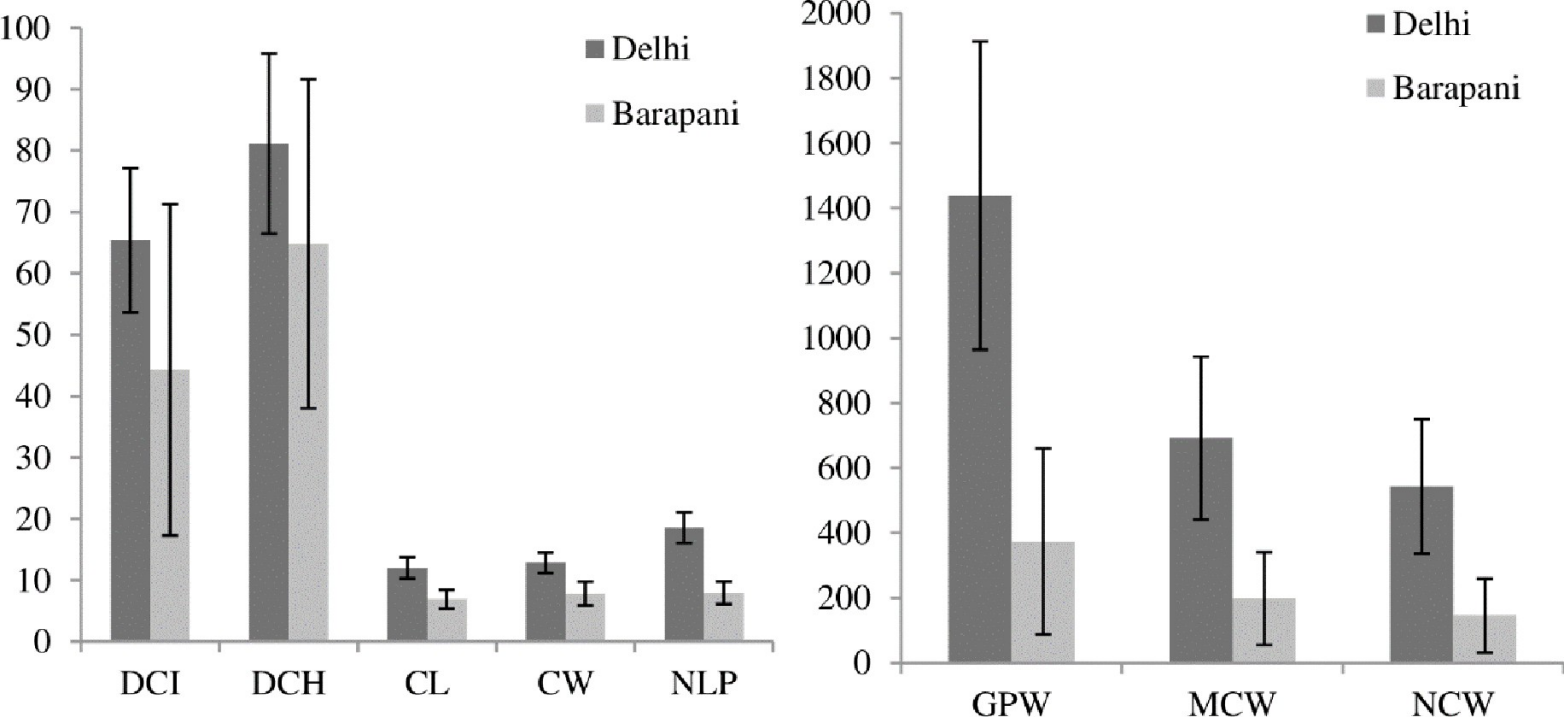
Comparison in mean values of agro-morphological traits at two Delhi and Barapani locations. DCI- Days for 50% curd initiation, DCH- Days for 50% curd harvesting, CL- curd length (cm), CW- curd width (cm), GPW–gross plant weight (g), MCW- marketable curd weight (g) and NCW–net curd weight (g).

Significant variations were observed between and within the maturity groups of cauliflower for all the eight observed agro-morphological traits (Table 1). Location effect was evident on the observed traits in 91 common genotypes. Overall, the mean values of the traits of each maturity group were significantly higher at Delhi location than at Barapani centre (Fig 1). The DCI (days to 50% curd initiation) in cauliflower genotypes was ranged from 48.0 to 93.3 days at Delhi and 16.7 to 123.0 days at Barapani (Table 1). Mean values at both locations were 65.41 days and 44.27 days, respectively. Pusa Meghna, DC-33-8, Pusa Kartiki and 30-Early took minimum period for DCI at Delhi and CC-14, CC-15, DC-903 and Early Kunwari at Barapani centre. Curd initiation took maximum days in KT-6 (123.0 days) followed by KT-20 (121.6 days), PSBK-1 (120.3 days) and KT-22 (116.7 days) at Barapani centre Genotypes showed wide range for DCH (days after transplanting) at Delhi (60–120 days) and Barapani (32.0–141.3 days). Maximum days for curd initiation were taken by KT-22, KT-2, KT-6 and Kt-25 at Delhi location and KT-22, PSBK-1, KT-6 and KT-17 at Barapani.

**Table 1:** ANOVA of agro-morphological traits of cauliflower at Delhi and Barapani centres.

Traits	Mean		Minimum		Maximum		MSS				CD (5%)		
	Delhi	Barapani	Delhi	Barapani	Delhi	Barapani	Factor A (df = 1)	Factor B (df = 90)	Interaction A × B (df = 90)	Error (n = 362)	Factor (A)	Factor (B)	Factor (A × B)
Days to 50% curd initiation	43.9	65.8	16.7	19.3	65.3	123.0	65956.0	1033.4	1513.4	7.8	0.47	3.17	4.48
Days to 50% curd harvest	62.0	83.8	34.0	32.0	79.7	141.3	64491.5	1177.6	1313.4	8.7	0.50	3.36	4.75
Curd length (cm)	8.6	10.4	3.8	5.0	13.4	16.1	420.7	5.0	45.5	0.8	0.15	0.98	1.39
Curd width (cm)	9.8	11.0	4.2	4.6	16.0	16.6	191.2	6.6	49.4	1.2	0.18	1.23	1.74
Gross plant weight (g)	733.4	1078.9	129.2	160.0	2583.3	2883.3	16298680.8	431987.4	2026062.0	18960.7	23.19	156.41	221.20
Marketable curd weight (g)	347.5	542.0	62.0	65.0	1100.0	1416.7	5162453.8	116505.1	446368.2	2885.5	9.05	61.02	86.29
Net curd weight (g)	265.4	424.0	38.3	41.7	875.0	1091.7	3433594.3	74296.8	294559.4	2498.1	8.42	56.77	80.29
No. of leaves/plant	13.0	13.5	5.0	4.7	23.8	26.3	38.7	14.1	185.1	2.2	0.25	1.67	2.36

Curd length was ranged from 8.2 cm (VV) to 16.1 cm (Pusa Snowball Kt-25) with a descending order of Pusa Snowball Kt-25> Khenzan-6-1>KT-2>Pusa Snowball K-1>KT-2>KT-22. Wide variation for curd length (3.8–10.0 cm) was observed at Barapani. Likewise, curd width was recorded to be maximum in Pusa Snowball Kt-25 (16.6 cm) and minimum in DC-33-8 (7.7 cm) at Delhi. At Barapani, it ranged from 4.2 cm (DC-3023-2) to 11.8 cm (KT-2).

The highest gross plant weight was recorded in KT-22 (2883.3 g) and minimum in P-903 (475.0 g). Descending order was DC-310>BR-2>DC-310-22>KT-25>KT-17. Significantly low gross plant weight was recorded from Barapani centre which ranged from 129.2 g (DC-3025-5) to 1210 g (Pusa Snowball K-1). The descending order was Pusa Snowball K-1>KT-22>KT-6>KT-20>KT-2. Marketable curd weight ranged from 208.3g (P-903) to 1416 g (BR-2) with a grand mean of 690.7g at Delhi location. In Barapani condition, it was ranged from 62.0 to 633.3 g. It was maximum in KT-20 and minimum in DC-334 (62.0 g). Wide range for net curd weight was observed at both Delhi (108.3–1091.7g) and Barapani (38.8–540 g) locations. Number of leaf per plant also varied among the genotypes which ranged from 12.7 (DC-41-5) to 26.0 (BR-2) in Delhi condition and 4.7 (DB-6) to 12.3 (KT-13-1) at Barapani. Interaction among the 91 genotypes and two locations was worked out on the observed eight traits (Table 2). The genotypic effects were significant for all the traits indicating considerable divergence in the cauliflower germplasm. Location influenced all the traits under study and the genotype × location effects were also significant for all the eight traits.

**Table 2:** Observations on agro-morphological traits on cauliflower genotypes at Delhi and Barapani locations.

Genotypes	Days to 50% curd initiation		Days to 50% curd harvesting		Curd length (cm)		Curd width (cm)		Gross plant weight (g)		Marketable curd (g)		Net curd weight (g)		No. of leaves /plant	
	Delhi	Barapani	Delhi	Barapani	Delhi	Barapani	Delhi	Barapani	Delhi	Barapani	Delhi	Barapani	Delhi	Barapani	Delhi	Barapani
PES	62.0	42.0	74.0	58.3	11.9	6.5	13.7	7.9	1950.0	286.7	658.3	100.0	591.7	81.7	23.8	8.3
30 Early	54.0	26.3	67.3	45.3	10.3	5.8	12.2	10.1	1300.0	249.2	655.6	152.5	525.0	130.0	22.0	10.0
PusaKartiki	52.0	25.7	67.0	45.0	10.8	6.3	12.3	8.2	1316.7	206.7	625.0	108.3	466.7	85.0	19.7	7.7
DC-28	61.0	31.3	72.7	71.3	9.4	6.5	12.0	8.1	855.0	193.3	451.7	100.0	310.0	76.7	19.8	9.3
DC-8	59.7	23.0	72.7	39.0	10.7	4.9	12.3	7.0	1041.7	188.3	486.7	95.0	421.7	71.7	20.2	8.0
DC-327-14-8-3	60.0	33.0	72.0	53.0	11.1	6.1	11.8	6.1	1533.3	256.7	496.7	141.7	433.3	118.3	20.2	8.3
DC-85	54.3	20.0	68.3	58.0	11.6	6.7	14.0	8.2	1466.7	191.7	418.3	101.7	325.6	80.0	23.8	6.3
DC-33-8	51.7	28.0	64.7	52.0	9.3	7.0	7.7	8.9	1058.3	271.7	416.7	168.3	306.7	140.0	19.2	8.7
DC-209	56.7	22.0	70.3	40.0	10.8	6.5	12.9	8.7	1333.3	231.7	636.1	125.0	525.0	96.7	20.8	7.0
DC351aa	54.3	38.0	68.7	56.0	11.0	10.0	11.7	11.3	1066.7	381.7	508.9	243.3	358.3	201.7	17.3	8.3
P-903	54.7	18.3	68.0	41.3	8.4	6.6	11.0	8.4	475.0	208.3	208.3	103.3	108.3	76.7	15.7	7.7
DC-395aa	56.3	38.0	66.3	56.0	10.3	10.0	12.3	11.3	925.0	381.7	533.3	243.3	343.3	201.7	16.8	8.3
PusaMeghna	48.0	27.0	60.7	49.7	8.5	7.7	8.8	9.5	1133.3	300.0	352.8	186.7	276.7	156.7	15.7	11.0
DC-41-5	54.7	40.3	68.0	56.0	9.9	5.3	11.2	7.6	966.7	216.7	475.0	123.3	375.0	100.0	12.7	7.0
Raja	57.7	37.3	70.0	53.7	11.0	5.9	12.1	6.5	930.0	306.7	563.9	153.3	426.7	96.7	15.4	8.0
DC-63	54.7	32.3	66.3	77.0	11.3	4.5	10.2	4.5	993.3	151.8	497.2	65.2	344.4	47.7	16.9	6.3
SabourAgrim	62.7	30.3	73.3	78.7	10.8	4.3	11.2	4.2	1133.3	189.0	500.0	77.0	408.3	46.7	18.4	5.3
PusaAshwini	55.7	33.0	69.3	49.0	11.2	7.3	12.5	8.7	1444.4	225.0	638.9	131.7	489.2	110.0	17.8	8.0
SEL-113	54.7	32.0	68.0	53.0	10.4	6.7	11.8	7.4	1225.0	296.7	572.2	143.3	450.0	93.3	21.3	11.0
CC-12	54.7	36.7	69.7	46.0	9.8	6.2	12.9	8.5	941.7	280.0	406.2	173.3	320.0	146.7	16.5	6.3
SEL-121	55.7	41.0	70.0	53.0	10.6	6.6	11.5	7.9	1008.3	275.0	502.8	153.3	425.0	120.0	15.0	9.3
CC-13	57.0	33.7	70.3	50.3	11.1	7.0	11.7	8.2	1133.3	338.3	449.3	295.0	368.0	186.7	18.8	9.7
SEL-124	54.7	28.0	68.7	47.7	9.9	6.0	12.1	8.1	1001.7	288.3	525.0	161.7	408.3	126.7	18.8	8.3
CC-14	57.0	18.3	72.0	41.0	11.2	6.3	12.5	9.2	865.0	195.0	453.3	101.7	362.2	80.0	16.3	6.3
SEL-42	54.0	26.7	69.0	40.0	11.3	7.7	12.2	9.3	1116.7	335.7	533.3	193.0	451.7	148.3	20.5	10.7
CC-15	57.7	16.7	71.7	34.0	9.3	6.0	10.8	7.5	991.7	183.3	465.7	91.7	373.3	73.3	17.8	7.0
SEL-7	54.7	28.0	65.3	45.7	11.2	6.6	12.1	8.1	916.7	210.0	508.3	106.7	383.3	76.7	16.8	8.3
DC-3003-1	55.7	25.7	73.3	47.0	12.0	7.1	13.7	7.9	1308.3	284.2	521.7	184.2	401.7	156.7	19.3	6.7
SEL-71	57.0	27.0	70.7	46.3	10.3	5.8	11.7	6.3	933.3	212.5	525.0	114.2	379.5	86.7	16.2	6.7
DC-3023-2	57.0	38.3	72.3	61.3	12.4	3.8	12.5	4.2	1341.7	210.0	708.3	103.3	548.3	71.7	19.7	7.3
SEL-9	57.0	29.7	71.0	69.3	11.6	5.0	12.3	5.3	1150.0	187.5	625.0	89.2	491.7	68.3	13.7	6.7
DC-3025-5	57.7	24.3	72.0	45.0	12.6	4.8	13.3	7.1	1375.0	129.2	541.7	237.5	424.0	100.0	18.0	7.7
VV	55.3	25.3	67.0	67.0	8.2	5.1	11.2	5.1	908.3	169.7	286.7	80.0	206.7	60.3	20.3	6.7
DC-3030-2	60.3	33.0	73.7	47.3	12.4	6.5	12.2	7.4	1258.3	206.7	663.3	296.7	510.0	183.3	15.3	8.0
PusaDeepali	61.7	32.0	74.0	69.3	11.3	4.3	12.0	4.3	1075.0	174.0	525.0	72.3	405.0	47.3	15.5	6.3
Early Kunwari	54.7	18.7	65.7	69.7	8.6	4.7	11.8	5.1	1008.3	186.7	320.0	88.3	253.3	65.0	19.0	6.7
DC-308	59.7	38.7	74.7	59.0	12.6	5.8	15.8	5.0	2200.0	185.0	925.0	85.0	650.0	60.0	19.7	5.7
DC-309	63.7	30.0	77.7	48.3	13.4	7.8	16.0	9.6	2200.0	340.0	883.3	190.0	625.0	135.0	22.7	7.3
DC-310	61.3	39.3	78.3	56.3	12.5	4.2	13.5	4.7	2583.3	177.0	1100.0	77.0	875.0	50.3	20.8	5.3
DC-321	61.7	30.3	75.7	51.7	11.1	7.2	12.8	10.5	1200.0	237.3	641.7	113.0	441.7	76.7	16.7	7.0
DC-325	64.7	24.0	79.7	35.0	11.6	5.9	11.1	4.6	1258.3	160.0	630.6	62.0	516.7	38.3	17.7	5.0
DC-334	65.3	32.7	79.3	48.7	11.1	4.0	13.2	4.3	841.7	183.3	588.9	85.0	396.7	61.7	16.3	6.7
DC-351	60.0	41.0	76.7	61.7	12.0	9.3	11.5	8.6	1633.3	241.7	658.3	133.3	541.7	103.3	16.8	5.0
DC-352	54.3	29.7	72.0	43.0	11.5	6.1	11.3	6.2	1041.7	240.0	566.7	153.3	383.3	121.7	18.0	7.0
DC-383	60.0	22.7	74.0	34.3	11.6	6.1	12.7	5.1	1433.3	199.7	650.0	82.7	483.3	51.7	22.3	5.3
DC-385	64.3	32.7	78.7	47.7	11.9	5.0	10.2	7.0	1213.3	187.3	616.7	80.3	506.7	50.0	20.7	6.7
DC-402	65.0	50.3	80.3	62.7	11.6	8.2	12.4	9.8	1466.7	476.7	575.0	253.3	466.7	161.7	21.3	10.3
DC-522	59.7	31.3	70.7	47.7	10.2	7.1	13.5	6.5	1550.0	188.0	575.0	82.3	451.7	56.7	21.7	6.3
DC-754	54.3	25.7	72.3	45.3	11.8	6.2	10.5	5.4	968.3	193.3	476.0	81.7	400.0	53.3	15.0	5.0
DC-260-328	59.3	50.3	75.7	62.3	12.9	5.2	12.6	5.3	1333.3	175.0	570.8	76.7	435.0	51.7	18.0	5.7
DC-310-22	60.0	24.7	75.7	32.0	13.0	7.3	13.7	5.4	2383.3	191.7	950.0	88.3	791.7	60.0	20.8	5.0
DC-BR-36	61.7	49.7	75.0	75.0	11.8	8.4	13.2	10.7	1033.3	496.7	515.0	306.7	385.0	220.0	18.0	8.7
DC-CCM*HR	64.7	56.7	78.0	76.7	11.9	9.8	13.2	11.7	1816.7	355.0	541.7	200.0	445.0	146.7	19.3	7.0
DC-DB-15	64.3	49.0	82.0	61.7	12.9	7.2	13.2	7.9	1258.3	403.3	771.7	190.0	631.7	110.0	15.7	9.0
DC-DB-6	68.3	22.0	82.0	36.7	12.1	5.6	12.5	4.6	1166.7	189.2	561.7	84.2	429.3	56.7	15.8	4.7
Himgiri	61.0	23.3	73.3	34.0	12.6	7.6	13.0	4.9	1225.0	194.2	508.3	79.2	418.3	46.7	15.3	5.7
DC-IJ-1	66.0	25.0	83.0	40.7	12.7	6.2	12.2	7.2	1600.0	160.0	716.7	65.0	605.0	41.7	16.0	6.3
PCF-102	68.3	29.3	83.0	45.0	11.4	5.6	11.9	7.8	1690.0	255.0	650.0	143.3	513.3	110.0	20.3	5.7
PCF-278	73.0	19.3	88.7	47.7	12.5	9.7	12.0	9.6	1016.7	303.3	550.0	196.7	396.7	166.7	13.2	8.0
PCF-373	60.0	24.7	76.0	46.0	14.3	7.6	15.0	8.7	2000.0	223.3	900.0	123.3	766.7	96.7	18.0	8.0
PusaSharad	59.3	26.0	75.7	41.0	11.7	7.5	11.1	10.2	1200.0	249.0	645.8	138.0	491.7	110.0	19.5	6.3
DC-466	81.3	34.3	100.0	62.0	12.3	5.1	12.9	5.1	1419.4	214.2	750.0	92.5	581.7	60.0	16.7	5.7
DC-476	87.7	38.7	107.3	60.3	13.0	7.2	13.8	8.7	1746.7	486.7	918.1	308.3	743.3	240.0	21.0	7.3
DC-18-19	72.0	52.7	89.0	76.0	13.6	7.2	15.0	8.5	1283.3	410.0	586.9	220.0	408.3	150.0	17.8	9.3
DC-3-5-1-2	72.7	47.3	91.0	63.0	11.6	8.0	11.8	9.7	1308.3	396.7	612.2	216.7	454.2	146.7	16.5	9.3
BR-1	70.7	52.7	84.7	78.3	12.8	6.8	11.8	9.2	1166.7	490.0	551.7	270.0	462.5	176.7	20.8	8.7
BR-161	72.3	54.0	87.7	80.0	14.3	8.2	12.8	9.6	1312.5	401.7	755.0	211.7	613.3	125.0	20.2	9.0
BR-2	71.7	56.0	87.0	83.0	14.3	6.7	16.0	8.4	2508.3	385.0	1416.7	175.0	1091.7	103.3	26.3	8.7
BR-202-2	71.7	53.7	87.7	78.3	12.6	8.3	12.5	8.9	1432.5	433.3	805.0	230.0	692.5	156.7	22.7	8.0
BR-207	72.7	54.0	87.7	80.0	13.3	8.4	12.5	9.3	1446.7	441.7	775.2	280.0	635.0	223.3	20.6	9.7
CC-32	62.0	53.7	78.7	68.7	12.7	9.3	14.1	9.7	1258.3	391.7	812.5	188.3	670.8	160.0	17.8	9.3
DPCa-5	76.0	52.7	97.0	74.0	13.7	7.1	13.2	8.8	1670.0	366.7	941.3	193.3	730.0	123.3	20.2	8.3
DPCa-7	73.7	49.0	93.0	61.7	12.9	7.2	14.9	7.9	2090.0	403.3	872.2	190.0	701.7	110.0	19.3	9.0
Lawyana	72.3	107.7	94.7	122.0	14.3	6.7	14.9	8.9	1550.0	533.3	925.0	283.3	713.3	180.0	16.7	7.7
PusaShukti	70.0	52.7	92.3	76.0	14.0	7.2	14.5	8.5	2191.7	410.0	1158.3	220.0	948.3	150.0	20.8	9.3
DC-SM	76.3	43.0	92.3	63.0	13.1	6.7	12.5	7.4	1692.0	411.7	833.3	191.7	667.0	110.0	19.5	11.0
EC-162587	72.0	56.0	87.7	71.7	14.3	8.3	12.8	8.3	1700.0	565.0	945.3	350.0	754.0	276.7	21.5	8.7
Kalapatta	92.0	45.7	116.3	60.0	14.4	7.8	16.3	9.7	1403.3	458.3	968.3	278.3	825.0	206.7	15.3	8.7
Khenzan-60-1	83.3	47.0	107.7	78.3	15.6	7.3	15.2	5.1	1808.3	224.3	902.5	98.3	706.7	61.7	20.7	5.0
KT-13-1	88.0	111.3	106.3	128.7	13.3	8.8	15.0	7.6	1941.7	893.3	975.0	400.0	766.7	313.3	16.0	11.8
KT-17	91.3	99.3	109.0	117.3	13.8	8.4	15.2	7.4	2233.3	950.0	1116.7	526.7	947.5	453.3	19.7	11.3
KT-178	88.3	112.0	105.7	128.3	13.5	9.9	14.8	8.5	1958.3	920.0	1108.3	400.0	884.0	338.3	20.0	10.0
KT-18	87.7	104.7	107.7	119.7	14.4	8.1	14.2	10.3	1950.0	1063.3	1246.7	586.7	984.2	353.3	20.7	10.7
KT-25	86.3	91.7	109.0	111.0	16.1	8.3	16.6	10.0	2250.0	1123.3	1241.7	480.0	1016.7	370.0	19.0	10.3
KT-2	92.7	106.0	118.0	120.7	15.2	8.6	16.0	11.8	2516.7	1136.7	1220.8	610.0	995.0	366.7	19.3	9.7
KT-20	92.3	121.7	111.3	141.0	14.5	9.2	15.0	10.7	2016.7	1246.7	1125.0	633.3	872.0	526.7	18.5	10.7
KT-22	92.0	116.7	120.3	132.3	15.1	8.9	15.4	9.6	2883.3	1303.3	1141.7	596.7	871.7	501.7	18.3	12.3
KT-6	93.3	123.0	116.3	138.0	15.0	9.3	14.8	8.7	1883.3	1250.0	1106.7	633.3	841.7	540.0	16.2	10.3
PSBK-1	83.0	120.3	106.3	141.3	15.3	9.4	16.3	10.0	1981.7	1310.0	1123.3	583.3	864.0	483.3	19.2	11.7
GPMT-100	62.7	36.7	77.7	58.0	9.5	7.0	12.3	7.3	1059.2	340.0	386.7	190.0	301.0	136.7	17.8	9.3
First Group	58.7	45.3	73.3	74.0	11.3	4.3	12.7	6.0	1441.7	245.0	579.2	121.7	483.3	88.3	17.3	9.3
C.D. (5%)	4.5	4.0	5.7	3.1	1.5	1.3	1.8	1.7	306.0	64.0	113.5	45.5	107.3	38.2	2.8	1.8

**Fig 2:**
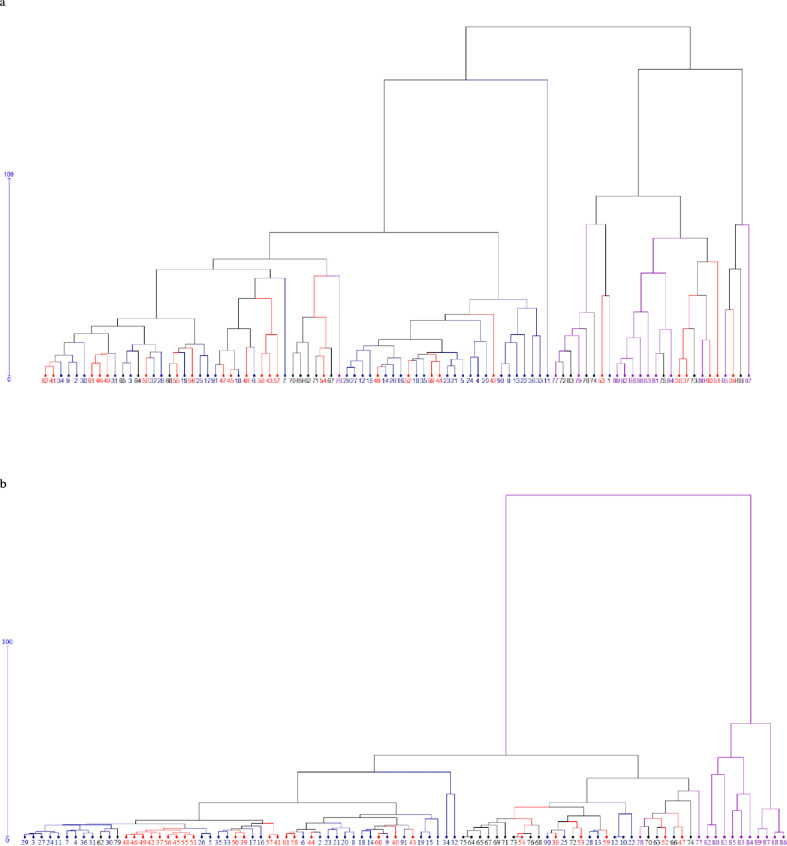
Dendrogram depicting relationship among cauliflower genotypes based on morphological traits observed at Delhi (a) and Barapani (b) centres. Blue- Early, Red- Mid-early, Black- Mid-late, Pink- Late or snowball.

Clustering of 91 genotypes through DARwin 6 software using the observations from eight agro-morphological traits from Delhi condition revealed two main clusters (Fig 2A). Each cluster had two sub-clusters. Sub-cluster 1a had majority of genotypes from early and mid- early group of cauliflower while sub-cluster 1b consists of only one genotype DC 903. In cluster 2, most of the genotypes were of mid-late and snowball groups while some of genotypes of mid-early group such as DC-308, DC-309, DC-CCM*HR, DC-310, DC-310-22 and PCF-373. In Barapani condition, there are two distinct clusters (Fig 2B). In that, cluster 1 consists of all the early, mid-early and mid-late cauliflower along with one genotype from snowball group (EC-162587). The cluster two consists of 10 genotypes of snowball group (KT-13-01, KT-17, KT-178, KT-18, KT-25, KT-2, KT-20, KT-22, KT-6 and PSBK-1.

### Molecular diversity

**Fig 3:**
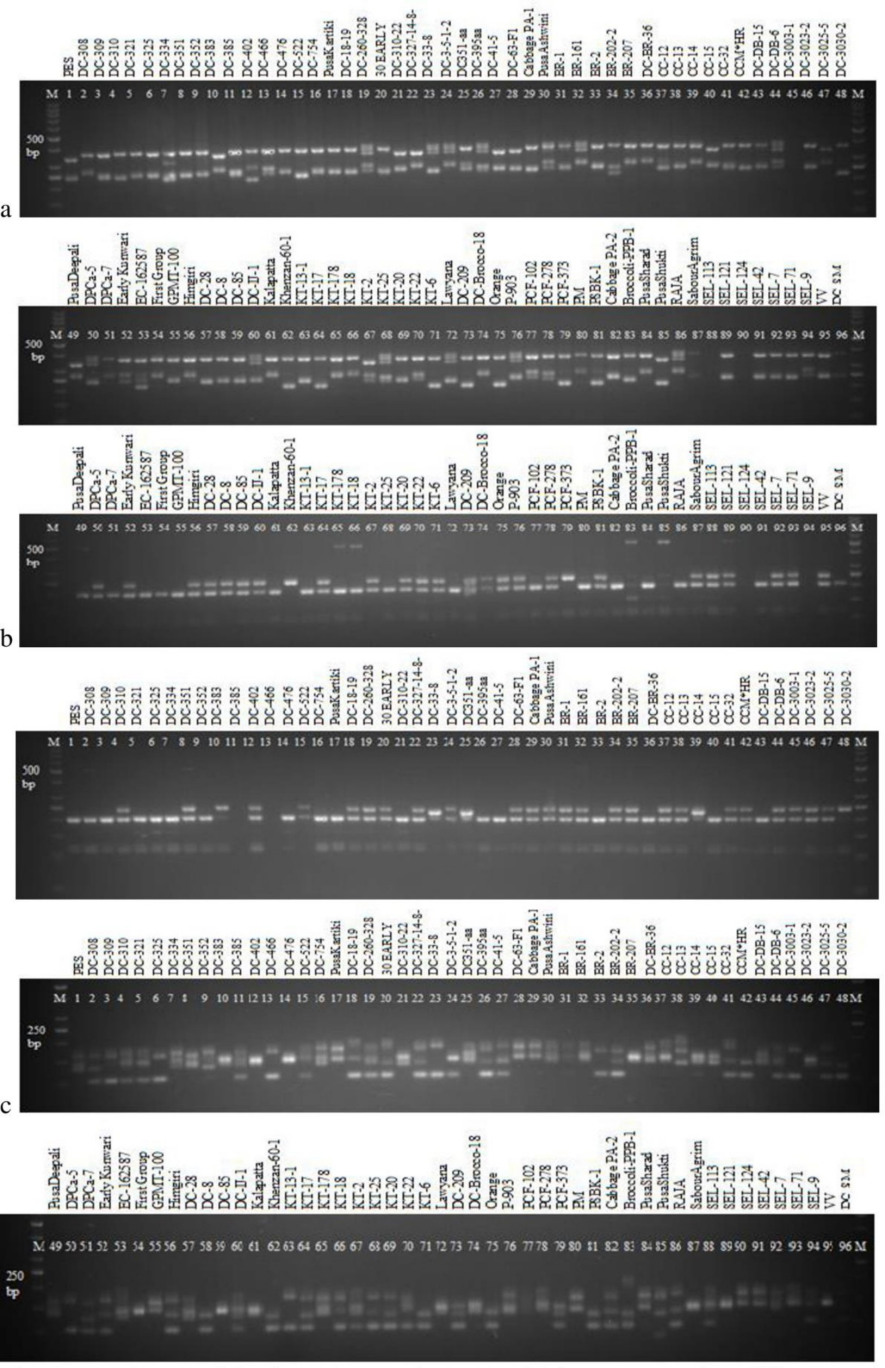
PCR amplification of genomic DNA of 96 genotypes of cauliflower and related crops with SSR markers. BoGMS0742 (a), BoGMS 0929 (b) and OI 10DO3 (c). M- Marker.

Molecular diversity was assessed in 96 diverse genotypes including 91 of white cauliflower, one of orange cauliflower and two each of broccoli and tropical cabbage. For this, 232 genomic SSR primers from *Brassica oleracea* group were screened with 96 genotypes. Among them, 95 showed good amplification in almost genotypes. Of them, 59 were polymorphic. The amplification of selected SSR markers is shown in Fig 3A–3C. Information on different polymorphic primers with their major allele frequency, number of genotypes amplified, number of alleles, gene diversity and polymorphic information content (PIC) value are presented in Table 3. Numbers of bands amplified by 90 primers were ranged from 1 to 9, with the mean value of 2.16.

**Table 3:** SSR Loci, major allele frequency, gene diversity, heterozygosity value (He), number of alleles andPIC value in the cauliflower genotypes.

Marker	Major Allele frequency	No. of genotypes amplified	No. of alleles	Band size	No. of polymorphic allele	Gene diversity	Hetero-zygosity (He)	PIC value	f value
BoSF2747	0.75	73.00	3.00	250,300	2	0.40	0.12	0.35	0.70
BoSF2212	0.95	83.00	3.00	300,320	2	0.10	0.11	0.10	-0.05
FITO348	0.61	87.00	4.00	225,250,275	3	0.48	0.02	0.38	0.95
BoPM14	0.68	92.00	3.00	200,220,250	3	0.48	0.15	0.43	0.69
BoESSR303	0.92	94.00	3.00	250,300	2	0.15	0.12	0.14	0.22
BoESSR165	0.92	96.00	2.00	800,900	2	0.14	0.05	0.13	0.64
BoESSR371	0.82	54.00	4.00	250,275,300	3	0.30	0.02	0.28	0.94
BoESS*R2*62	1.00	84.00	1.00	160	0	0.00	0.00	0.00	-
BoESSR333	0.94	96.00	2.00	325,350	2	0.12	0.08	0.11	0.29
BoESSR935	0.71	92.00	3.00	500,550,600	3	0.45	0.24	0.41	0.47
BoESSR343	0.72	91.00	2.00	120.160	2	0.40	0.56	0.32	-0.38
Cnu286	0.93	54.00	2.00	175,250	2	0.14	0.00	0.13	1.00
BoSF1537	0.51	91.00	3.00	225,300	2	0.51	0.99	0.38	-0.96
BoSF2717	0.64	96.00	2.00	150,175	2	0.46	0.30	0.35	0.35
BoGMS0692	0.53	96.00	2.00	300,350	2	0.50	0.00	0.37	1.00
BoSF1613	0.93	74.00	4.00	250,260	2	0.14	0.11	0.14	0.24
Na10D01	0.54	92.00	3.00	125,150,160,	3	0.53	0.00	0.43	1.00
BoSF1163	0.84	96.00	2.00	400,600	2	0.27	0.32	0.23	-0.19
BoSF1103	1.00	14.00	1.00	300	0	0.00	0.00	0.00	-
BoESSR934	1.00	95.00	1.00	100	0	0.00	0.00	0.00	-
BoESSR702	1.00	94.00	1.00	300	0	0.00	0.00	0.00	-
BoESSR122	0.69	94.00	3.00	375,400	2	0.44	0.00	0.36	1.00
BoPM16	0.51	96.00	2.00	225,400	2	0.50	0.99	0.37	-0.98
BoPM14	0.88	96.00	2.00	200,250	2	0.22	0.00	0.19	1.00
BoPM21	1.00	93.00	1.00	225	0	0.00	0.00	0.00	-
BoPM-6	1.00	91.00	1.00	450	0	0.00	0.00	0.00	-
Boe878	1.00	79.00	1.00	100	0	0.00	0.00	0.00	-
SoRA43	1.00	96.00	1.00	175	0	0.00	0.00	0.00	-
BoGMS0632	0.95	86.00	3.00	260,270	2	0.10	0.02	0.10	0.77
BoGMS0348	0.76	89.00	2.00	175,200	2	0.37	0.10	0.30	0.73
Ni4-B06	1.00	79.00	1.00	120	0	0.00	0.00	0.00	-
Na14-E11	0.49	90.00	3.00	90,120	2	0.51	0.99	0.39	-0.93
Na10-G06	1.00	94.00	1.00	240	0	0.00	0.00	0.00	-
BOSF1252	0.51	92.00	2.00	100,250	2	0.50	0.99	0.37	-0.98
BoGMS0596	0.59	90.00	2.00	150,175	2	0.48	0.81	0.37	-0.68
BoGMS0374	0.92	79.00	3.00	500,520	2	0.14	0.00	0.14	1.00
BoGMS0327	0.49	89.00	3.00	220,250	2	0.55	0.99	0.46	-0.78
BoGMS0162	0.50	92.00	3.00	100,125,250	3	0.59	1.00	0.51	-0.69
BoGMS1464	0.84	92.00	2.00	250,300	2	0.27	0.00	0.24	1.00
BoGMS1452	0.50	71.00	2.00	100,150	2	0.50	1.00	0.38	-1.00
BoGMS0941	0.75	92.00	3.00	250,275,300	3	0.41	0.39	0.37	0.04
BoESSR719	1.00	92.00	1.00	300	0	0.00	0.00	0.00	-
BoESS*R2*16	1.00	59.00	1.00	200	0	0.00	0.00	0.00	-
BnGMS490	1.00	94.00	1.00	350	0	0.00	0.00	0.00	-
BoGMS1465	0.79	96.00	2.00	350,375	2	0.33	0.00	0.28	1.00
BrBAC231	1.00	96.00	1.00	300	0	0.00	0.00	0.00	-
BoGMS0929	0.69	91.00	2.00	200,250	2	0.43	0.54	0.34	-0.25
BoGMS0742	0.39	93.00	9.00	250,350,400	3	0.73	0.99	0.68	-0.36
BoGMS1164	0.59	95.00	2.00	250,275	2	0.48	0.00	0.37	1.00
BoGMS1510	1.00	95.00	1.00	225	0	0.00	0.00	0.00	-
BoGMS0692	0.72	94.00	2.00	325,350	2	0.40	0.00	0.32	1.00
BoSF1637	1.00	88.00	1.00	50	0	0.00	0.00	0.00	-
BoSF1163	0.78	77.00	2.00	400,600	2	0.34	0.36	0.28	-0.05
BoESS*R2*51	0.88	93.00	2.00	250,260	2	0.22	0.25	0.19	-0.14
BoGMS1432	0.99	82.00	2.00	250	0	0.02	0.00	0.02	1.00
BoSF1162	1.00	79.00	1.00	275	0	0.00	0.00	0.00	-
BoSF1166	0.58	83.00	2.00	275,300	2	0.49	0.00	0.37	1.00
BoSF1269	1.00	88.00	1.00	250	0	0.00	0.00	0.00	-
Na10-B08	1.00	48.00	1.00	50	0	0.00	0.00	0.00	-
BoESSR145	0.85	61.00	2.00	250,260	2	0.25	0.07	0.22	0.74
BoSF1205	0.99	92.00	2.00	260	0	0.02	0.00	0.02	1.00
BOSF1210	0.95	87.00	3.00	225,275	2	0.09	0.00	0.09	1.00
BoSF1212	0.51	96.00	2.00	175,275	2	0.50	0.99	0.37	-0.98
BoSF1221	0.87	75.00	2.00	250,260	2	0.22	0.07	0.20	0.70
BOSF1302	1.00	94.00	1.00	250	0	0.00	0.00	0.00	-
BrMS015	1.00	68.00	1.00	250	0	0.00	0.00	0.00	-
CB1034A	0.89	88.00	3.00	100	0	0.20	0.10	0.19	0.49
CB10623	0.84	91.00	3.00	150,160	2	0.28	0.07	0.26	0.77
SORA26	1.00	47.00	1.00	50	0	0.00	0.00	0.00	-
Oi13C12	0.58	85.00	4.00	400,600	2	0.50	0.80	0.39	-0.60
OI10D03	0.55	95.00	5.00	100,150,175	3	0.61	0.59	0.56	0.05
BoSF1004	0.79	79.00	7.00	250	0	0.36	0.10	0.34	0.72
OI12F02	0.93	90.00	4.00	150,250	2	0.13	0.00	0.12	1.00
BoGMS1307	0.94	94.00	2.00	200	0	0.12	0.13	0.11	-0.06
BoGMS0394	0.50	95.00	2.00	100,150	2	0.50	1.00	0.38	-1.00
BoGMS0083	0.79	95.00	2.00	200,250	2	0.33	0.42	0.28	-0.26
Na10D-09	0.88	85.00	2.00	150	0	0.21	0.00	0.19	1.00
Na10DD11	0.98	87.00	3.00	150	0	0.03	0.01	0.03	0.67
BoPM15	0.97	69.00	2.00	150,250	2	0.06	0.00	0.05	1.00
MYb28A09	1.00	92.00	1.00	300	0	0.00	0.00	0.00	-
BoGMS0576	1.00	16.00	1.00	150	0	0.00	0.00	0.00	-
Na12-F03	1.00	96.00	1.00	175	0	0.00	0.00	0.00	-
MYb28B1	1.00	96.00	1.00			0.00	0.00	0.00	-
BOGMS0952	1.00	96.00	1.00	250	0	0.00	0.00	0.00	-
BoESSR186	1.00	93.00	1.00	225	0	0.00	0.00	0.00	-
BoSF1202	1.00	93.00	1.00	250	0	0.00	0.00	0.00	-
BoSF1202	1.00	91.00	1.00	150	0	0.00	0.00	0.00	-
O10B02	0.95	91.00	5.00	150,250	2	0.10	0.02	0.09	0.77
CB10179	1.00	85.00	1.00	150	0	0.00	0.00	0.00	-
CB10179	1.00	92.00	1.00	225	0	0.00	0.00	0.00	-
Mean	0.84	85.34	2.16	250,300	2	0.21	0.19	0.18	0.12
Minimum	0.39	14.00	1.00			0.00	0.00	0.00	-1.00
Maximum	1.00	96.00	9.00			0.73	1.00	0.68	1.00

PIC- Polymorphic information content; NaN- Not.

The highest number of alleles were generated to be 9 by BoGMS0742 followed by BOSF1004 (7), O10B02 (5) and OI10D03 (5). Four alleles were observed from FITO348, BOESSR371, BOSF1613, OI13C12 and OI12F02 markers. Eighteen primers amplified 3 alleles, 31 primers 2 alleles and 32 primers amplified only one allele. Two gene-based STS markers Myb28A09 and Myb28B1 linked to glucosinolates content in *Brassica juncea* [34, 35] also analysed in the germplasm and Myb28B1 marker was found to be monomorphic while Myb28A09 could amplify one band in 92 genotypes.

The polymorphic information content (PIC) was highest for BoGMS0742 (0.68) followed by OI10D03 (0.56) and BoGMS (0.51), BOPM (0.43). Twenty-four SSR markers had PIC value in range of 0.0 to 0.25 which indicates for low polymorphism level. Overall, mean PIC value of 90 SSR was 0.18 but 32 of these SSRs had PIC value 0. The mean of PIC value of 58 SSR markers (0.28) had moderate polymorphism with mean PIC value of 0.27. Average gene diversity was 0.21 and it was maximum in BoGMS0742 (0.73) followed by OI10D03 (0.61), BoGMS0162 (0.59) and BoGMS0327 (0.55). Out 90 markers, 58 had gene diversity in range of 0.02 to 0.73 and 32 markers did show 0 value.

**Fig 4:**
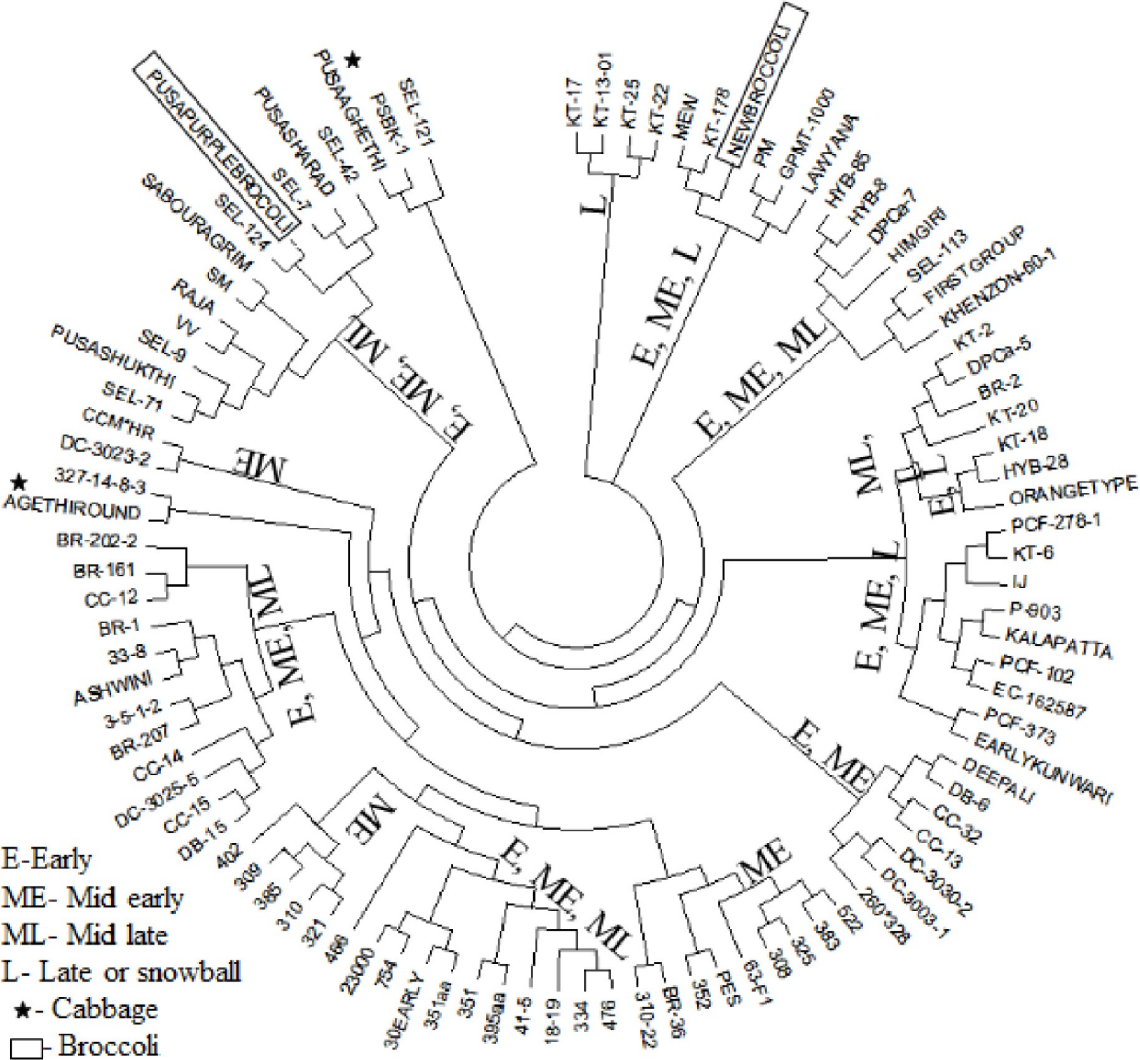
Dendrogram showing dissimilarity using UPGMA cluster analysis demonstrating association among 96 genotypes of cauliflower and related crops.

The UPGMA dendrogram obtained from SSR markers analysis could reveal four clusters of 96 genotypes as shown in the Fig 4. Cluster I had four genotypes (KT-17, KT-13-01, KT-26 and KT-22), cluster II consists of six genotypes DC 209, KT-178, DC-Brocco-18, Pusa Meghna, GPMT-1000 and Lawyana. Cluster III was the largest cluster with maximum number of genotypes (83). It was further sub-divided into two sub-clusters, one with three genotypes including two of cauliflower (PSBK-1 and Sel-121) and one of cabbage (PA-2). Genotypes of Broccoli (Delhi Purple Broccoli-1 or ‘DPB-1’ and DC-Brocco-18) and cabbage genotypes (‘PA-1’ and ‘PA-2’) did not group in crop specific clusters.

**Fig 5:**
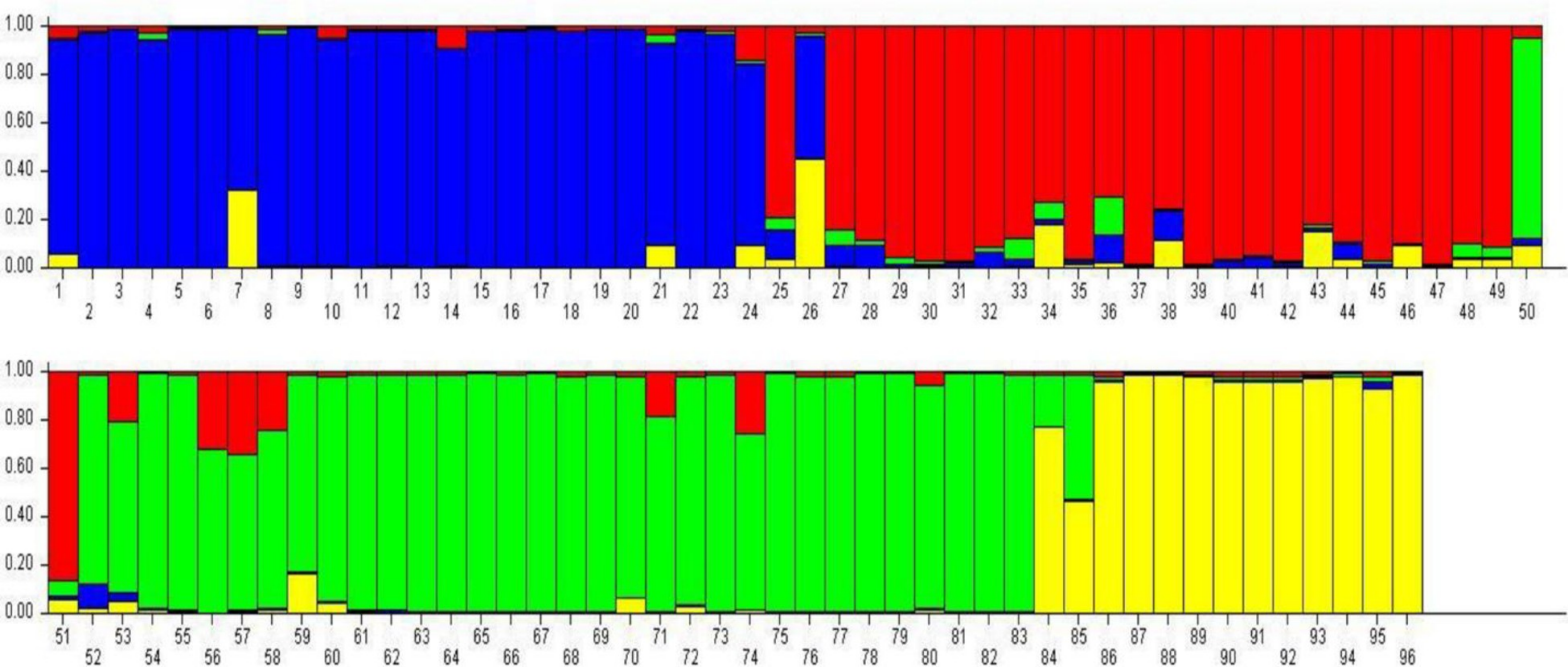
Analysis of substructure of the cauliflower genotypes using STRUCTURE software. Each genotype is represented by a vertical bar, which is partitioned into K colored segments that represent individual’s estimated membership coefficient (Q) to the K (= 4)clusters (STRUCTURE2.3.4). Four subpopulations were G1-Blue, G2-Yellow, G3-Red and G4-Green.

The STRUCTURE 2.3.4 based analysis of genotyping data from 90 SSR markers with K value from 2 to 10 and revealed the showed the population of the 96 genotypes was a mixed population consisting of four-sub-populations. These subpopulation groups were denoted as G1, G2, G3 and G4 and comprised of 24, 25, 34 and 13 genotypes, respectively (Fig 5). Almost 52 genotypes including 17 of G1, 9 of G2, 20 of G3 and 6 of G4 showed no admixture while remaining had small to large extent of admixture for the investigated SSR markers. Only one genotype No. 25 (DC 351aa) had admixture from four groups while No. 11 (DC 385) had from three subpopulations and 25 had a mixture of two groups. Almost all subpopulations contained genotypes from different groups of curd maturity. These results indicate that the grouping of genotypes on the basis of SSR markers was inconsistent with the traditional curd maturity-based grouping of the genotypes (*r*^*2*^ = 0.00031).

However, main cluster II had all the genotypes from late/snowball group. Sub-clusters in main-cluster III also had genotypes predominantly from mid-early group namely DC 402, DC 309, DC 385, DC 310 and DC 321. Similarly, DC 522, DC 383, DC 325 and DC 308 genotypes of mid-early also made a single sub-cluster. Two of the four newly developed genotypes (DC 3030–2 and DC-3003-1) from open market materials could make part of one sub-cluster. DC 23000 (Pusa Kartiki), DC 754, 30-Early and DC 351aa genotypes of early maturity groups also clustered in same group. However, most of the sub-clusters had genotypes from different maturity groups.

## Discussion

### Agro-morphological diversity

Cauliflower is a thermo-sensitive crop and there are four groups of cauliflower made on the basis of temperature requirement for curding traits namely early (20–27°C), mid-early (16–20°C), mid-late (12–16°C) and late or snowball (10–16°C) [15]. Small curds (200–600 g) of early group and large size curds (>1000 g) are associated to late or snowball group [8] which is due to growing days and temperature factor during curd initiation and development stages. Genotypes of different maturity groups had variation for phenological traits namely curd initiation, curd maturity and curd size at two locations which proved the influence of temperature on expression of these traits. Since, the climate of Barapani centre was relatively cool and damp than Delhi condition, and cool temperature and high relatively humidity favour early curd initiation. The curd maturity is positively correlated with curd traits indicating that the prolonged growth period increases curd weight. Accurate identification and characterization of germplasm is important for cultivar development and breeder’s rights protection [36]. In Delhi and Barapani conditions, only two main clusters were observed from eight agro-morphological traits. [13] studies 52 genotypes of snowball cauliflower and reported 10 clusters. [24] studied diversity in 45 genotypes of cauliflower from early (3), mid-early (7), mid-late (8) and snowball (7) groups, besides 20 collections from other countries namely China (6), Russia (5) and Netherlands (8). They used only few genotypes in early and mid-groups which were not sufficient to depict the available diversity in Indian cauliflower. The present study analysed large set of diverse germplasm including early (37), mid-early (25), mid-late (15) and snowball (13) groups of cauliflower. Morphological variation in the genotypes was in the line of previous reports on Indian cauliflower [16, 17, 37, 38], Irish cauliflower collections [39] and snowball cauliflower [13, 14]. We found some promising genotypes at both locations for marketable curd weight namely CC-13, Pusa Meghna and DC-903 in early groups, DC-BR-36, DC-476, BR-2 and DC-18-19 in mid-late group, KT-6, KT-20, PSBK-1 and KT-2 in late or snowball group. Of them, CC-13 is a self-incompatible line hence can be of immediate use for hybrid breeding.

### Molecular diversity

Molecular markers are useful tools to estimate the extent of genetic diversity present in the germplasm. The SSR markers are still widely used and preferred over other marker systems such as RAPD, RFLP, and AFLP for their procedural requirements, robustness, and reproducibility [40]. The SSR markers have been well employed in understanding the genetic variations *Brassica* species for diversity studies [20, 23, 41, 42]. Earlier, RAPD, ISSR and SSR markers have been used for linkage analysis in cauliflower for characterization of self-incompatible lines [43], genetic diversity analysis [44] and linkage analysis with downy mildew [45, 46] and black rot [47]. The SSRs with high polymorphism are useful for parent selection, mapping of specific traits and their introgression. In present study, 90 polymorphic markers generated good amount of diversity in 92 genotypes of cauliflower and two each of broccoli and cabbage. However, these SSRs could not separate out broccoli and cabbage from cauliflower lines. This might be due to the fact that the cabbage, cauliflower and broccoli are evolved from a common source *Brassica sylvestris* during the evolutionary process with major and minor mutations for physiological arrests at different stages of growth and developmental stages. Further, the SSRs used in the present study were not specific to the regions of the chromosome(s) which demarcates differences in these three crops. Similar results were also presented by Lowe et al. [48] while explaining use of SSRs in *Brassica* species. Slipped strand mis-pairing and occurrence of insertion and deletion during the evolutionary processes of these crops might have contributes such morphological changes [41] and the SSRs used in present study were not targeted for these mutations.

The SSR based dendrogram revealed that (i) all the four main clusters and most of their sub-clusters had mixed set of genotypes from three or more maturity groups, (ii) certain sub-clusters (i.e. sub-cluster-IIa) and few nodes in sub-cluster IVa had genotypes particular to maturity group such as sub-cluster IIa (KT-17, KT-13-01, KT-25 and KT-22 of late group) and three nodes of sub-cluster IVa (one, DC-321, DC-310, DC-385, DC-309; two: DC-402, CCM*HR and DC-3023-2; three: DC-522, DC -383, DC-325 and DC-308), (iii) in one sub-cluster, the genotypes of mid-late and late group were placed together, and (iv) the genotypes of broccoli and cabbage were grouped alongwith cauliflower and they could not make separate clusters. Similar results were earlier reported by Vanlalneihi et al. [44] while studying genetic diversity using 26 SSR markers in 48 genotypes of three maturity groups of Indian cauliflower. The SSR markers could corroborate the agro-morphological grouping to some extent with respect to Indian and Snowball cauliflower only. But, the distinction was not enough as expected, which could be due to limited extent of variation at genetic level, because most of the growth and developmental traits were common in both groups, however, the level and intensity of expression traits had variation.

Polymorphic information content (PIC) value for primer is important indicator for level of polymorphism to use in molecular studies. The PIC value > 0.5 indicates for high polymorphism, 0.25–0.5 for moderate polymorphism, and <0.25 for low polymorphism [49]. The PIC values in the present investigation indicated low to moderate level of diversity. Moreover, only three markers had PIC value higher than 0.50 namely BoGMS0742, OI10D03 and BoGMS0162 which can be considered to be highly polymorphic while 31 SSRs had PIC value in moderate range. The observations agree with the earlier reports of average PIC value of 0.316 by Zhu et al. [22] while studying diversity in 165 cauliflower inbred lines primarily derived from southeast China. However, our observations were less than the PIC value of 0.571 reported by El-Esawi et al. [21] in Ireland collection which could be due their only 12 SSRs and 25 genotypes from diverse pool.

We observed good extent of diversity which was reflected grouping of cauliflower genotypes, however, it does not establish that the extent of diversity in the investigated genotypes was narrow. Tonguç and Griffiths [24] demonstrated least SSR diversity in cauliflower probably because they used genotypes of a narrow gene pool. Astarini et al. [50] reported diversity in cauliflower genotypes from Lembang in Western Java and showed its relatedness with current cultivars from India and the Australian.

Therefore, it is suggested to use a greater number of markers having well distribution in all the nine chromosomes to make a conclusive study on the extent of diversity in the cauliflower germplasm in India. Overall, the effectiveness of SSR markers in assessing the extent of diversity in cauliflower agreed with earlier reports of Astarini et al. [50], Plieske and Struss [51] and Li et al. [52].

The SSR marker-based subpopulation structures of cauliflower are not consistent with the agro morphological groups. STRUCTURE analysis of genotyping data made four sub-groups with prominence of admixture. It revealed that all three groups of Indian cauliflower had genetic regions from each other and also from snowball group. Similarly, the regions in snowball group were also matched with the Indian types. Thus, the present study could reveal that the present day Indian cauliflower germplasm was evolved as a result of intentional or natural intermixing between typical Indian types and introduced improved varieties/lines. The SSR marker-based analysis showed a varied level of heterozygosity in the tested genotypes of cauliflower. Further, these markers could not group two genotype of each cabbage and broccoli in separate groups, indicating for presence of sufficient genetic variation in these genotypes. These results are in conformity with the findings of Zhu et al. [22]. They investigated 165 cauliflower inbred lines from southeast China using 43 SSR markers and inconsistency between STRUCTURE based subpopulation and agro-morphological traits (curd maturity, curd solidity or geographical origins) based grouping. The information with 90 SSR markers is partially consistent with the functional grouping of few genotypes for curd initiation and maturity groups. Since, curding is a genetically complex trait and influenced by environmental factors [5, 53]. Admixture in the genotypes could be due to introgression of genomic fragments in genotypes of F_6_ to F_15_ generations. Further, snowball group genotypes have introgression from European cauliflower and few genotypes of Indian cauliflower had genomic regions from exotic tropical types They are under purification or advance breeding stages as also revealed by the genomic SSR markers.

The first detailed analysis of genotypes from all the four diverse maturity groups at two distinct locations could reveal that (i) the curd initiation and development are critical to temperature factor in cauliflower, (ii) agro-morphological observations revealed that the grouping of cauliflower genotypes on the basis of temperature requirement for curd initiation and development was found to be effective (iii) the genetic diversity across the groups was revealed by the SSR markers but to small extent, and (iv) present day germplasm of Indian cauliflower had admixture from other maturity groups and also from snowball/European types. From this, we suggest that using of a greater number of SSR markers for identification of desired and novel alleles which will assist in DNA fingerprinting, genome mapping, linkage map construction, gene tagging etc. and pyramiding of these novel genes from different genotypes of cauliflower increases the diversity in order to obtain the valuable and desired hybridization combination for future use in breeding of cauliflower for marker assisted breeding.

## Supporting information

S1 Table Primer sequence used in present study. (DOCX)

S1 File (ZIP)

S1 Raw data (XLSX)
