# Supplementary material for: Agro-morphological and molecular diversity in different maturity groups of Indian cauliflower (Brassica oleracea var. botrytis L.)
Source: PLoS One. 2021 Dec 10;16(12):e0260246. doi: 10.1371/journal.pone.0260246 (PMC8664203; doi:10.1371/journal.pone.0260246)
Supplement: S1 Table — (DOCX) [file pone.0260246.s001.docx]

**Table S1. Primer sequence used in present study**

| **Sl. No.** | **Primer code** | **Primers Sequence (5’-3’)** | |
| --- | --- | --- | --- |
|  |  | **Forward primer (5’-3’)** | **Reverse primer (5’-3’)** |
| 1 | BoSF2747 | 5'GCAGGGGAGACTTGTGTCAT | 5’TATGCTTTGCTGTGCCTGAG |
| 2 | BoSF2212 | 5'GAACCCAAGGAAACATGCAC | 5’TCAGAGTGTCCTCTGCCTCA |
| 3 | FITO348 | 5'TGTATCTCAAACAAACCTGTAA | 5’AATCAAACTGCCACATAGGA |
| 4 | BoPM14 | 5'CAGAGGTGGAGTGAGAAAGA | 5’GATGAAGAATGGAACCCTAAA |
| 5 | BoESSR303 | 5'GAACCCACCTTCCTTCAACA | 5'GCGATTTTTCAGGAGAAGTC |
| 6 | BoESSR165 | 5'GTTTGACCCCGTCAGATGAT | 5'GACTTCGACGCTTCACCTTC |
| 7 | BoESSR371 | 5'GAGATGTCGACGTGACTGGA | 5'CCTCCTCCACAACGGTCTAA |
| 8 | BoESSR262 | 5'ACACCACCGACAAGATCTCC | 5'GCTTCGCCACGTTTATCTTC |
| 9 | BoESSR333 | 5'CCTTGGTCTTCTCCGATGAG | 5'ATGATCGTGAACGTCCCATT |
| 10 | BoESSR935 | 5'GGCTTCTCGCTAATTTCATT | 5’CGCCTCGATCAATCTTCTTC |
| 11 | BoESSR343 | 5'TTCTATGCGCCTCCCCTAAT | 5'GTTTCTGCACATCCGAACCT |
| 12 | Cnu286 | 5'AGTTGCCCCTATTCATGCAC | 5’AATGCGTTCATGTGGGGATA |
| 13 | BoSF1537 | 5'TCTTGAGAACACGGAGACCA | 5’TTCAACTTCTTGCTTCGTCG |
| 14 | BoSF2717 | F:5'CCGACAGCAAACAAGATCAA | R: 5’TCAGACCATCTGTTTGGTCG |
| 15 | BoGMS0692 | 5' AACTGTGTCTTGGATGTCTTG | 5’ AAGGTTAGTCGTGTCGTAAA |
| 17 | Na10D0 | 5'GAGACATAGATGAGTGAATCTGG | 5’CATTCGTTGTGGACGGTCGG |
| 18 | BoSF1163 | 5'GGTTGACCGCAATCATG | 5’TGGTCATGGTTGTGGTTTGT |
| 19 | BoSF1103 | 5'TCTCGGCATCTTATGGCTCT | 5’CCACACCGGAGTTTAGCTGT |
| 20 | BoESSR934 | 5'GTTCGCCAAATCCAAAAGC | 5'ATCTCACTTTCGCCATTGCT |
| 21 | BoESSR702 | 5'GTGGTTGTGTTGCTGGAATG | 5'TCTGATGACTCGCTCGAATG |
| 22 | BoESSR122 | 5'AATCGAAGCTATTCGTGTA | 5'CATGTGTTGGCTGTTTCAGG |
| 23 | BoPM16 | 5'ACGGATAAAGGAGGAGAAGA | 5’CTGCTTAGTTAGCGTTGAGAAG |
| 24 | BoPM14 | 5'CAGAGGTGGAGTGAGAAAGA | 5’GATGAAGAATGGAACCCTAAA |
| 25 | BoPM21 | 5'TAAGGGTGAGAAGCAGAGAG | 5’AAGTAAACCGATAGAATACCAGA |
| 26 | BoPM-6 | 5'GTCACAAGGATCAGAGGAAT | 5’GTTAGGGTTTAGAGTTGAGGG |
| 27 | Boe878 | 5'TTCTTGCTTTCCCAAGGAGA | 5’GACTCTGACAACGACGACGA |
| 28 | SoRA43 | 5'GCGCGTGTGGGATCAGAA | 5’CTTCTCCACCGTCGATCG |
| 29 | BoGMS0632 | 5'ATCATCGTCCTCTTCTTCTTC | 5’TATCATCCTTATTGGGTCTC |
| 30 | BoGMS0348 | 5'CGTGAGTGCTTCCTCTGT | 5’TCCTTTGTCATCTTCTCCAA |
| 31 | Ni4-B06 | 5'GGTAAGAAAATGTCTGCGCC | 5’TTGCTGCAACTTCTCATTCG |
| **Sl. No.** | **Marker code** | **Forward primer (5’-3’)** | **Reverse primer (5’-3’)** |
| 32 | Na14-E11 | F:5'TCATCCTTCTCACACCAAAATC | R: 5’CCTCGAAATAGCTCCAACCC |
| 33 | Na10-G06 | 5'TGAGAAGGGGAACAGTCGAG | 5’TGTGTTGTTTTGGCTTTTGG |
| 34 | BOSF1252 | F:5'CCAACTGCATACAAGTTCAA | R: 5’TCAGTCCATATGTAAGGTCG |
| 35 | BoGMS0596 | 5'AACGGAAAGTGAAACCTAAGA | 5’GAGCAAGACCATCTCCAAC |
| 36 | BoGMS0374 | 5'CATCTTCATCTCACACAAACA | 5’ATAACCGAAATAGCAAAGTAG |
| 37 | BoGMS0327 | 5'CCTCTATCTGTGGCTGTGATT | 5’CCTTTCTCTATGAAGTAGGCTG |
| 38 | BoGMS0162 | 5'ACTGTTTCTAAGCCATTGTTG | 5’TTCCCTCTCTCAAGGTGTAA |
| 39 | BoGMS1464 | 5'CTGATGAACGGAGACACAG | 5’AAGCAAAGCAGAGCATAAAC |
| 40 | BoGMS1452 | 5'CGGTGGGTGTGTAGTTAGTT | 5’TTCTATCAGTTCCAAGTTCCA |
| 41 | BoGMS0941 | 5'GTTGAAGAAACTAAGGAGGAAA | 5’GAACGACAGCGAAGAGAG |
| 42 | BoESSR719 | 5'TCTCTTTGCTCGTTCCACCT | 5’CTGCTAAGGGTGACGATGCT |
| 43 | BoESSR216 | 5'CGGAAGAAGACGTTGAGGAG | 5’GGTTTCCGCTATCCAGAA |
| 44 | BnGMS490 | 5'AGTTGGTAGCCAAAGTGAAA | 5’CTCTTATGCGATTCTCATCC |
| 45 | BoGMS1465 | 5'GAGGTGTTGGATACTGTGCT | 5’TGTTGTTGGTGTTAGTGGTG |
| 46 | BrBAC231 | 5'CCTTACCCGACTGATTTTGG | 5’CGCGGAAAATGTTTGAGTT |
| 47 | BoGMS0929 | 5'TCAGACCCAAAGCCAGTT | 5’TTGTGGAAGATGAAACCATT |
| 48 | BoGMS0742 | 5'TCTCTCTCCTCTTTGCTTTG | 5’GTATCAGACATTATTCACACGA |
| 49 | BoGMS1164 | 5'CGATTCAAACTCAAACCAAC | 5’AATAAAGAGACAGGGCGG |
| 50 | BoGMS1510 | 5'GCGAAAGGGTAGAGAAGAGT | 5’TATTTGGTTACAAGTGCGAAG |
| 51 | BoGMS0692 | 5' AACTGTGTCTTGGATGTCTTG | 5’ AAGGTTAGTCGTGTCGTAAA |
| 52 | BoSF1637 | 5'GGATTGCCTGAGTTTATTCTT | 5’GAGTGAATGTGTCATGTCTTCTCCTTCATTGA |
| 53 | BoSF1163 | 5'GGTTGACCGCAATCATGC | 5’TGGTCATGGTTGTGGTTTGT |
| 54 | BoESSR251 | 5'GAAGGGTCGATTACGATCCA | 5'GAAGTGGTCCAACCAGAGGA |
| 55 | BoGMS1432 | 5'AAGTCGGCACAAGGTGTT | 5’AGGGATTTAGAGTTTCGTGTT |
| 56 | BoSF1162 | 5'GTGCAAGGATAAACGGG | 5’TTCGCCTGTGAAACCTTC |
| 57 | BoSF1166 | 5'CTTGACCCATTTTGACCA | 5’CATGTCAACAAATCCCCC |
| 58 | BoSF1269 | 5'AATCAAATAGCCGCAGCG | 5’TCAAAAACCGTGACATCG |
| 59 | Na10-B08 | 5'AGAGAAAAACACTTCCCGCC | 5’GTGAGCTTTGCGAAACACG |
| 60 | BoESSR145 | 5'GGGCGAGGATGGTTACTATA | 5'TCATACCCCAAGGCTATTTT |
| 61 | BoSF1205 | 5'CCACCAGTTCCTGCAACT | 5’TTACTGAAAGCCAAACCG |
| 62 | BOSF1210 | 5'GGCCATACTAATTTTTCA | 5’CGTCAAAAGTTTATTAGA |
| 63 | BoSF1212 | 5'TAAGGAAGCAATCCACCA | 5’CTTTCCCAATCCTTTCCC |
| 64 | BoSF1221 | 5'CCCTCTTAAATTAACACA | 5’TGTCTTTCTTCTCCTAAT |
| **Sl. No.** | **Marker code** | **Forward primer (5’-3’)** | **Reverse primer (5’-3’)** |
| 65 | BOSF1302 | F:5'CCGACAGCAAACTAGATCTA | R: 5’TCAGCACATCTATGTGGTCG |
| 66 | BrMS015 | 5'TCGCCAATAGAACCCAAAACTT | 5’CATCTCCATTGCTGCATCTGCT |
| 67 | CB1034A | 5'TACGGGATAGGCGACGAA | 5’CCTAAGCGAGACGTATTG |
| 68 | CB10623 | 5'GAGATCGAAGGTCTCGGT | 5’GAGTCGAAACAGTGGTGG |
| 69 | SORA26 | 5'TGTTTACCTGTTGGAGAT | 5’AACCCTAAGCATCTGCGA |
| 70 | Oi13C12 | 5'AGAGGCCAACAAAGAACACC | 5’GAAGCAGCACCAGTGACAAG |
| 71 | OI10D03 | 5'AAGCCACGTGAAGAAAGTCC | 5’GCCAAAGACCTCAAAGATGG |
| 72 | BoSF1004 | 5'GAAGGCCCACATAAGGTT | 5’CGATCTAAATAGCGAGCC |
| 73 | OI12F02 | 5'GGCCCATTGATATGGAGATG | 5’CATTTCTCAATGATGAATAGT |
| 74 | BoGMS1307 | 5' TGGCGATAAAGAGGAGAAC | 5’ CGAAGAGAGAGAAACAAAGAA |
| 75 | BoGMS0394 | 5' CCCTTACTTTGTTCAGGTTTC | 5’ CATCTCTACCCACCACACA |
| 76 | BoGMS0083 | 5'GCAGAGATGAAAGAGATAAACA | 5’AACTCCTACCAGCGAGAAA |
| 77 | Na10D-09 | 5’AAGAACGTCAAGATCCTCTGC | 5’ACCACCACGGTAGTAGAGCG |
| 78 | Na10DD11 | 5'GAGACATAGATGAGTGAATCTGG | 5’CATTCGTTGTGGACGGTCGG |
| 79 | BoPM15 | 5'AGATTGTCCGTTCTTGTATTT | 5’CCCTGTTTCTTGTAGTGTTATG |
| 80 | MYb28A09 | 5’GAGTTGTCTCTCTCTCGGTCTC | TTGTCGGAGGAATCAGAATC |
| 81 | BoGMS0576 | 5'ACCTGGAGTTGAGACGGG | 5’CAGTGTTGAGTGTTCTTCTATT |
| 82 | Na12-F03 | 5'GGCGACATAGATTTGAACCG | 5’TCCACTTTCTCTCTCTTCCCC |
| 83 | MYb28B1 | AAGGGGCATGGACCACCGA | TATCCTCTTCATTAATCTGCTCAG |
| 84 | BOGMS0952 | 5’CAGTGAGTAACATTTGGCTG | 5’CGAGAGAGAAAGTGATGAGAG |
| 85 | BoESSR186 | 5'CTTCTTCTTTCGCAGCGTCT | 5'TGAAACCATCGTCCATGAAA |
| 86 | BoSF1202 | 5'GCCTTATCAGCTGTATCC | 5’CCAGTCCCAGTGATGTTG |
| 87 | BoSF1202 | 5'GCCTTATCAGCTGTATCC | 5’CCAGTCCCAGTGATGTTG |
| 88 | OI 10B02 | 5'ACTAAGCTATTAACATTGA | 5’GAATCCTGATTGCCTTAG |
| 89 | CB10179 | 5'ACGAAGCAAATAACAAAGA | 5’GAAACCCGAAAGCCTAAG |
| 90 | CB10179 | 5'ACGAAGCAAATAACAAAGA | 5’GAAACCCGAAAGCCTAAG |
