# Supplementary figures and images for: Agro-morphological and molecular diversity in different maturity groups of Indian cauliflower (Brassica oleracea var. botrytis L.) (part 2 of 2)
Source: PLoS One. 2021 Dec 10;16(12):e0260246. doi: 10.1371/journal.pone.0260246 (PMC8664203; doi:10.1371/journal.pone.0260246)

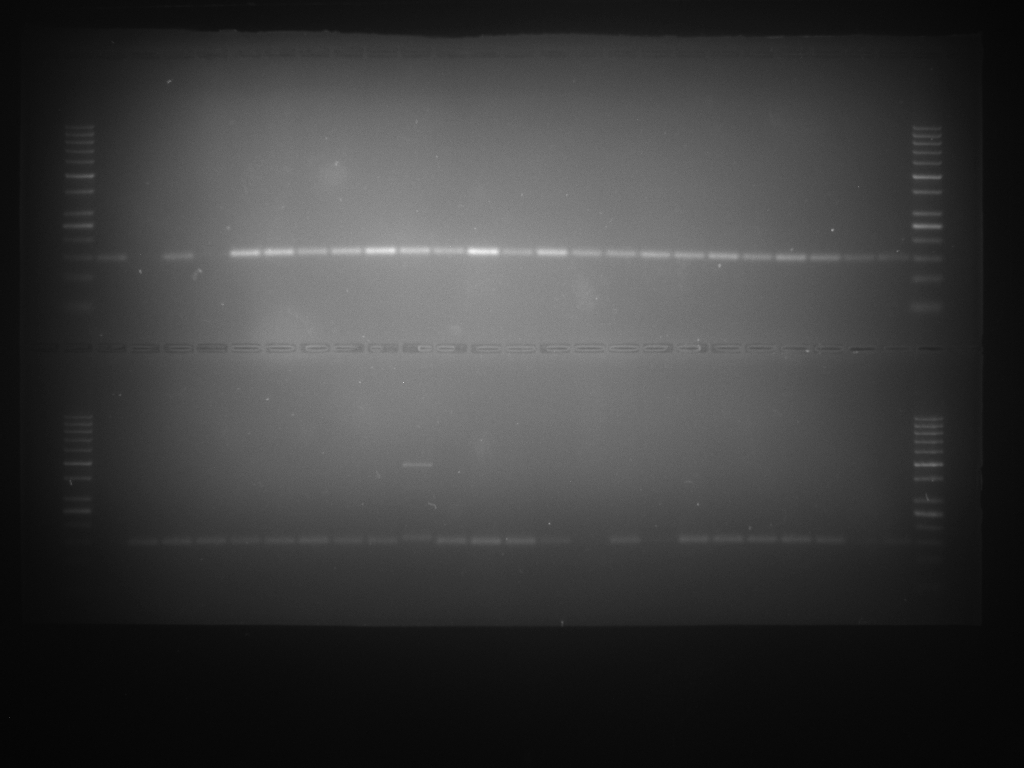

Supplement: S1 File — (ZIP) [file pone.0260246.s002.zip › OI10179 27-02-2020.jpg]

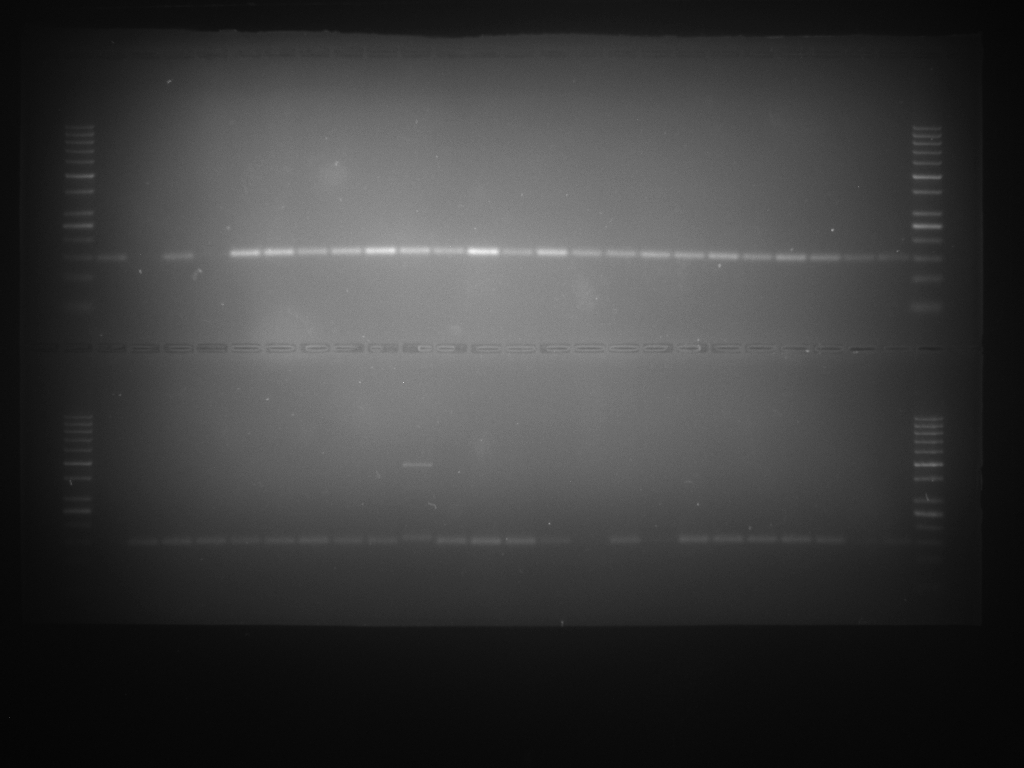

Supplement: S1 File — (ZIP) [file pone.0260246.s002.zip › OI10179 LANE3,4 27-02-2020.jpg]

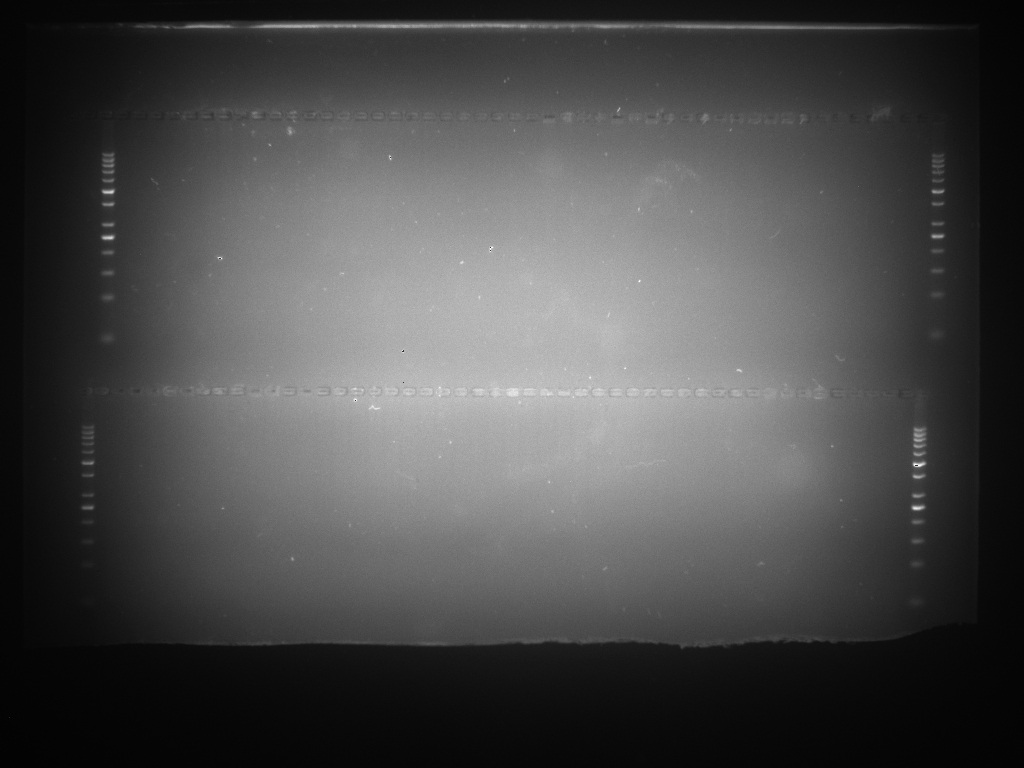

Supplement: S1 File — (ZIP) [file pone.0260246.s002.zip › RA2A04 27-2-2020.jpg]

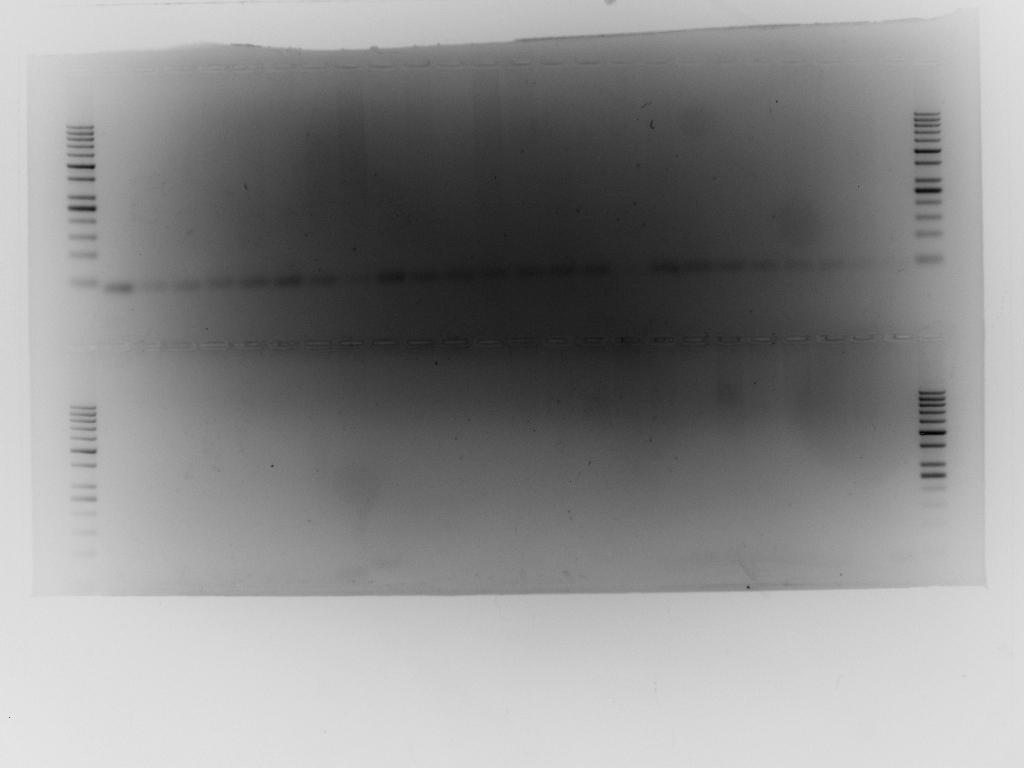

Supplement: S1 File — (ZIP) [file pone.0260246.s002.zip › REVERSE IMAGE GLR-5.jpg]

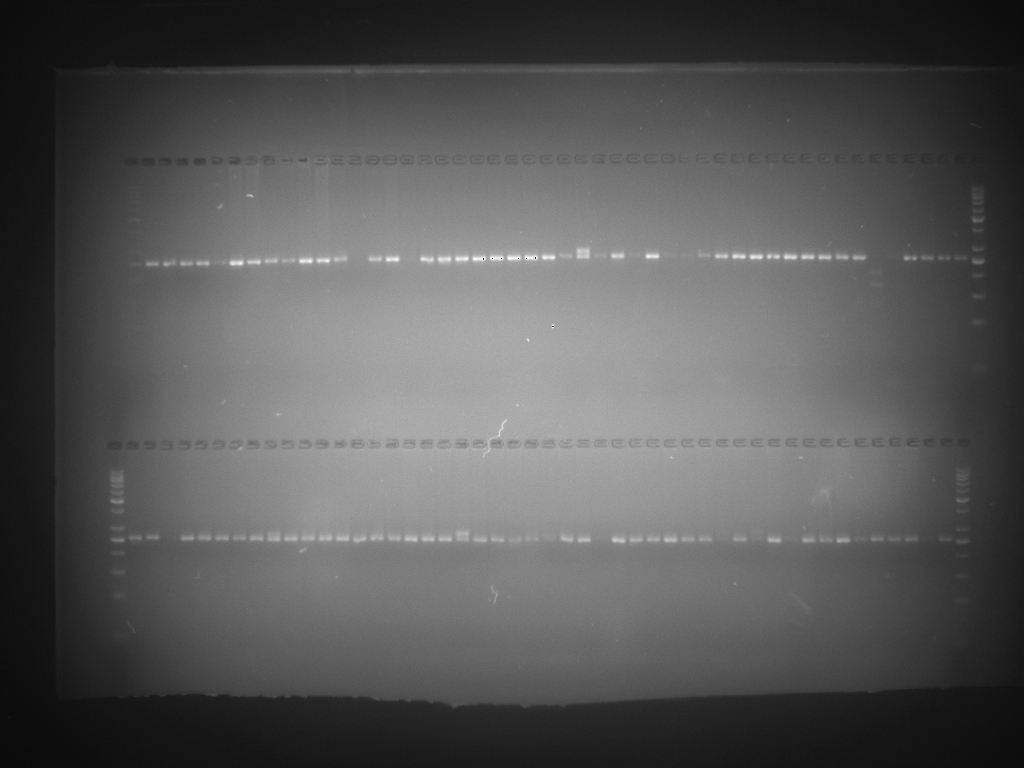

Supplement: S1 File — (ZIP) [file pone.0260246.s002.zip › ruskita - 1 1.2.2020.jpg]

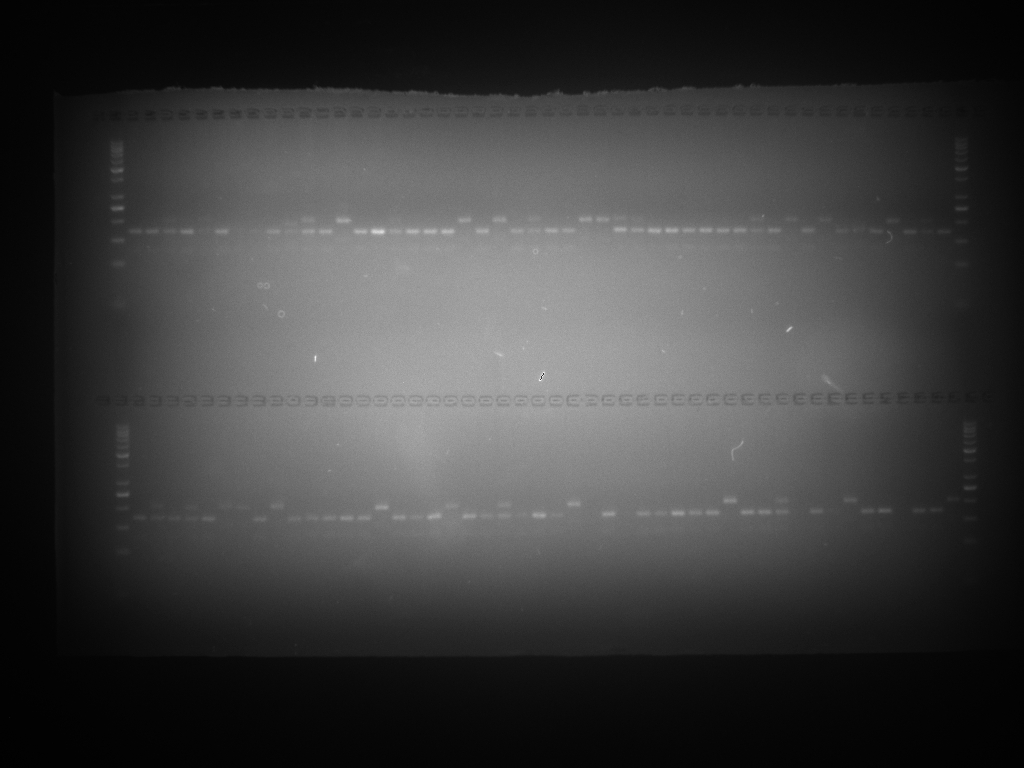

Supplement: S1 File — (ZIP) [file pone.0260246.s002.zip › ruskita - 2 1.2.2020.jpg]

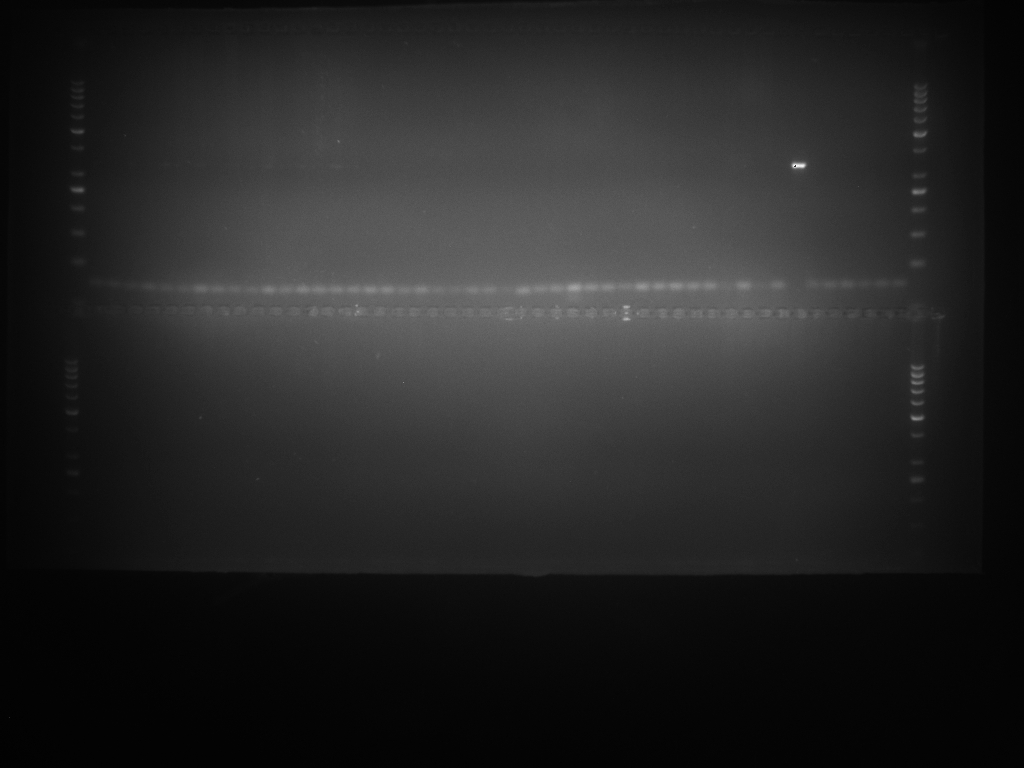

Supplement: S1 File — (ZIP) [file pone.0260246.s002.zip › SORA267 3-2-2020 LANE 3 AND 4.jpg]
