# Supplementary figures and images for: Agro-morphological and molecular diversity in different maturity groups of Indian cauliflower (Brassica oleracea var. botrytis L.) (part 1 of 2)
Source: PLoS One. 2021 Dec 10;16(12):e0260246. doi: 10.1371/journal.pone.0260246 (PMC8664203; doi:10.1371/journal.pone.0260246)

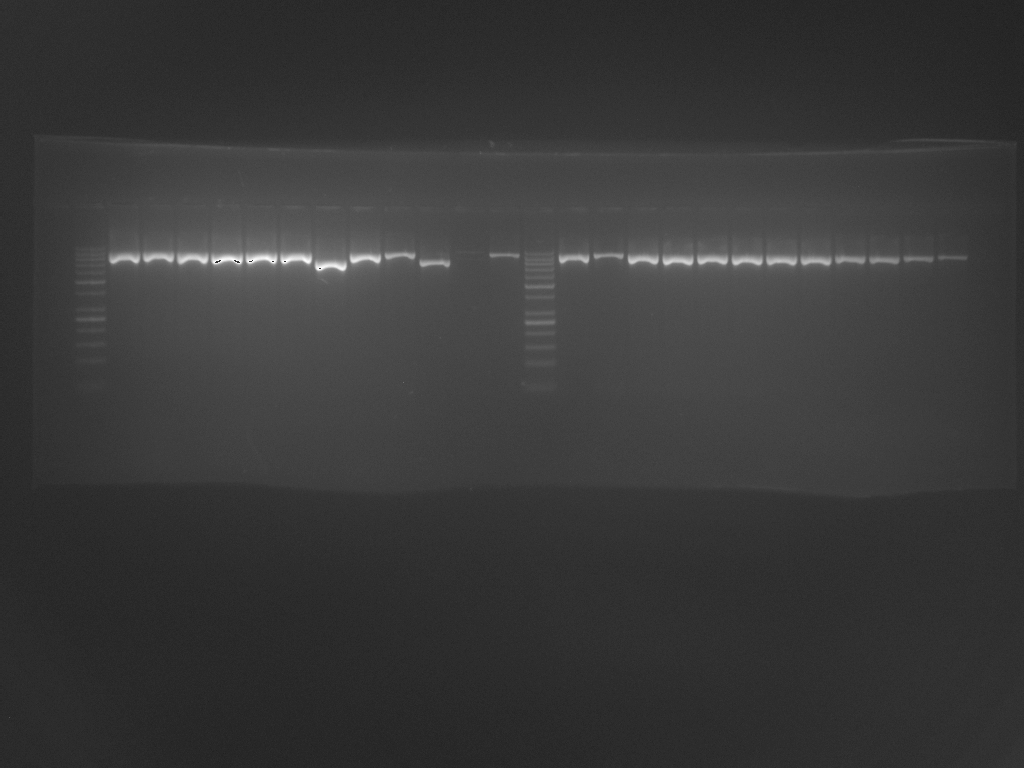

Supplement: S1 File — (ZIP) [file pone.0260246.s002.zip › 1a. ssr 165 lanee 1 31 12 2019.jpg]

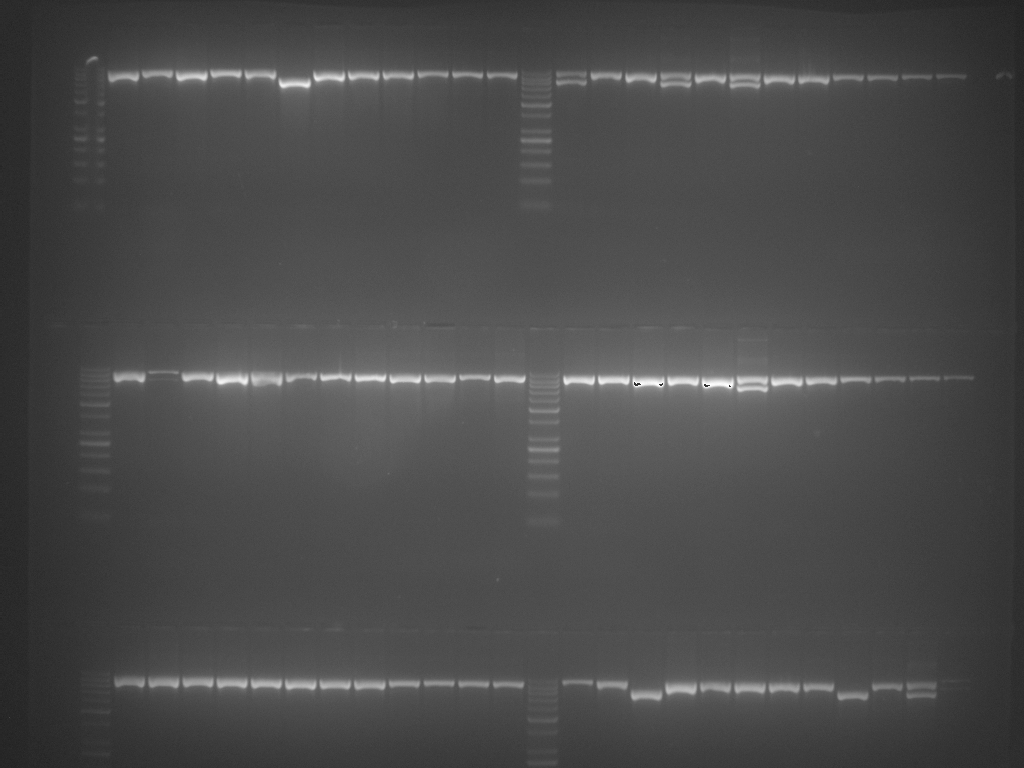

Supplement: S1 File — (ZIP) [file pone.0260246.s002.zip › 1b. ssr 165 lanee 234 31 12 2019.jpg]

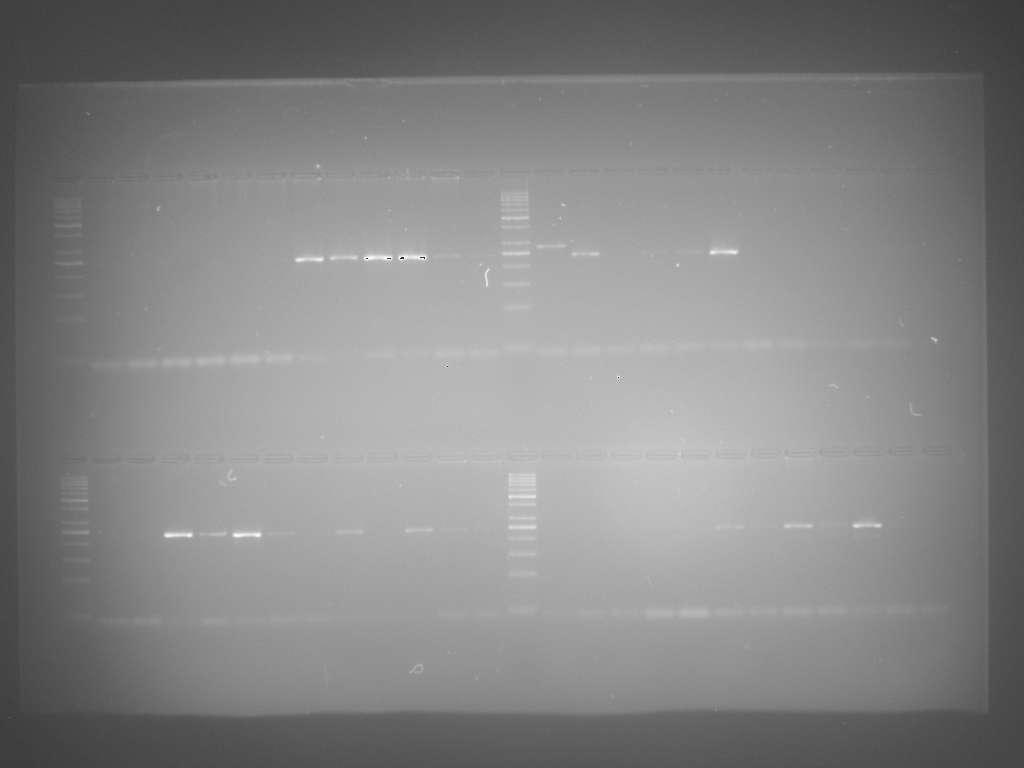

Supplement: S1 File — (ZIP) [file pone.0260246.s002.zip › 2a. SSR371 312020 LANE 1AND 2.jpg]

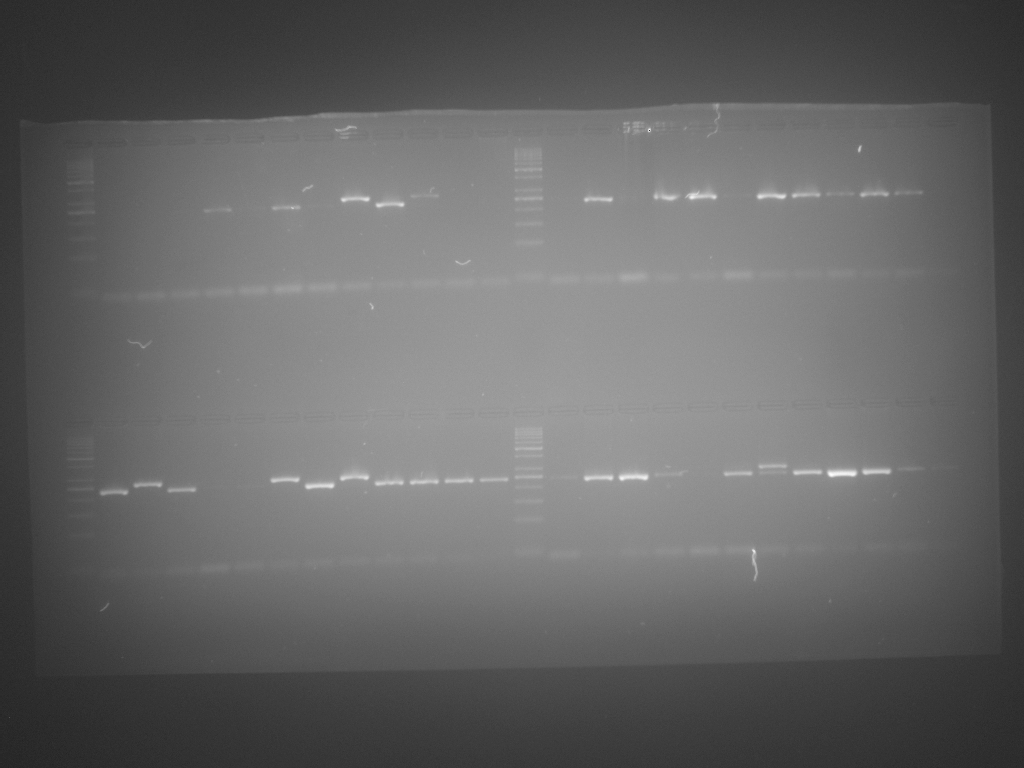

Supplement: S1 File — (ZIP) [file pone.0260246.s002.zip › 2b. SSR 371 312020 LANE 3 AND 4.jpg]

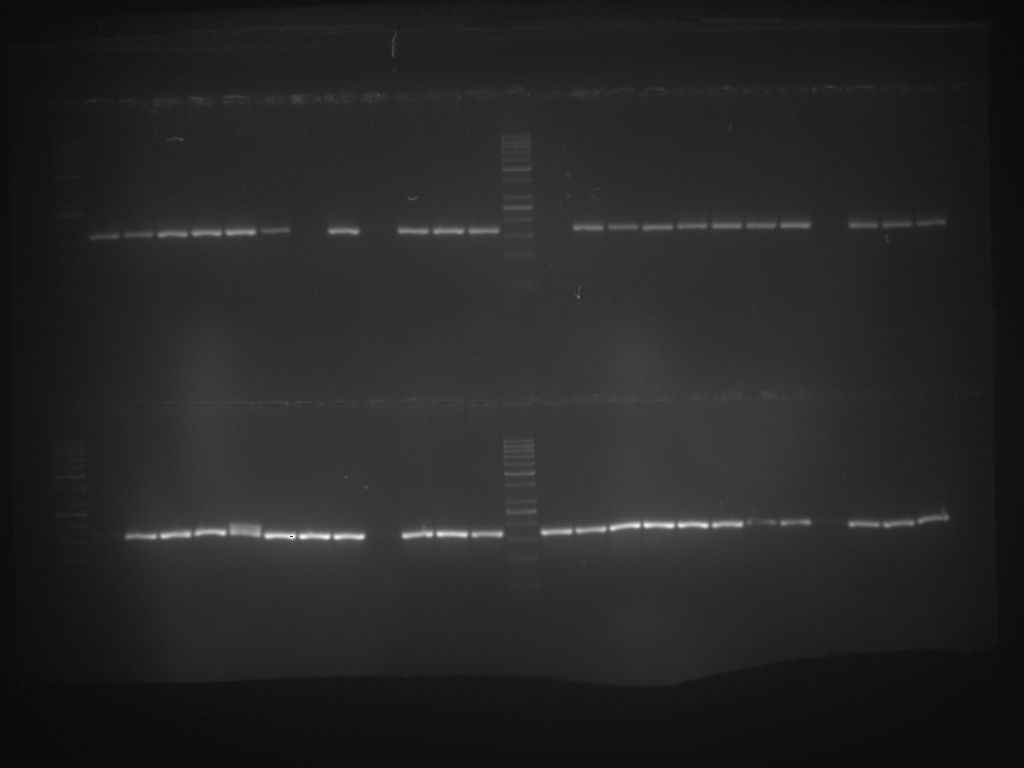

Supplement: S1 File — (ZIP) [file pone.0260246.s002.zip › 3a. lane 1 and 2 boessr 262 1,1.2020.jpg]

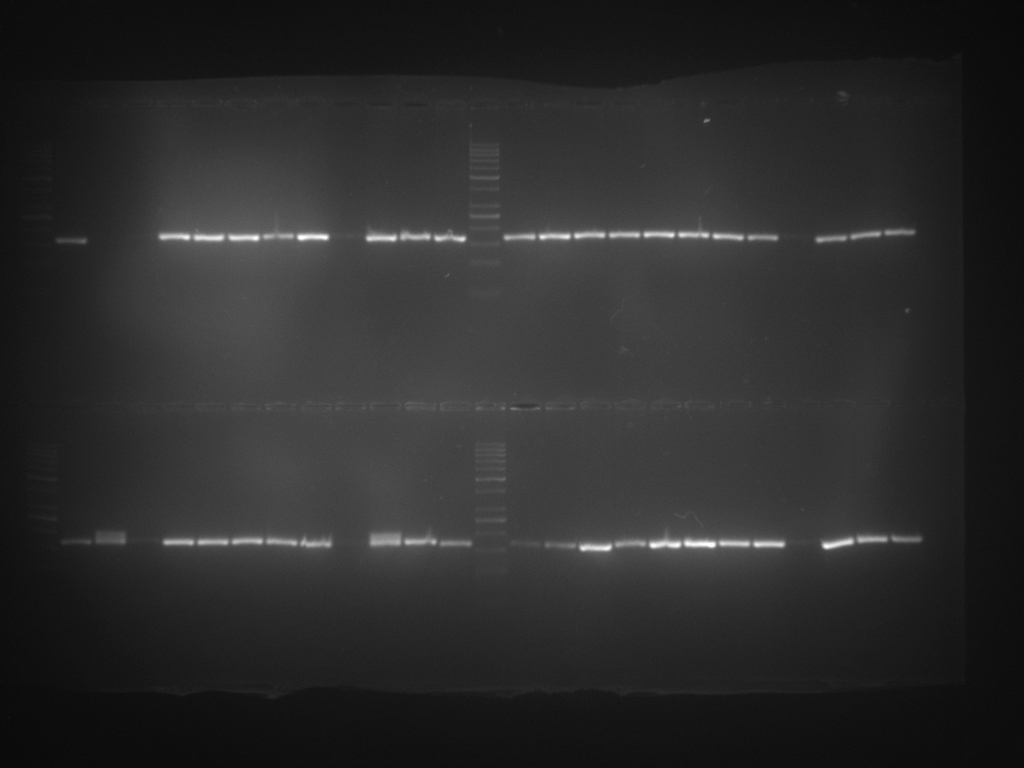

Supplement: S1 File — (ZIP) [file pone.0260246.s002.zip › 3b. lane 3 and 4 boessr 262 112020.jpg]

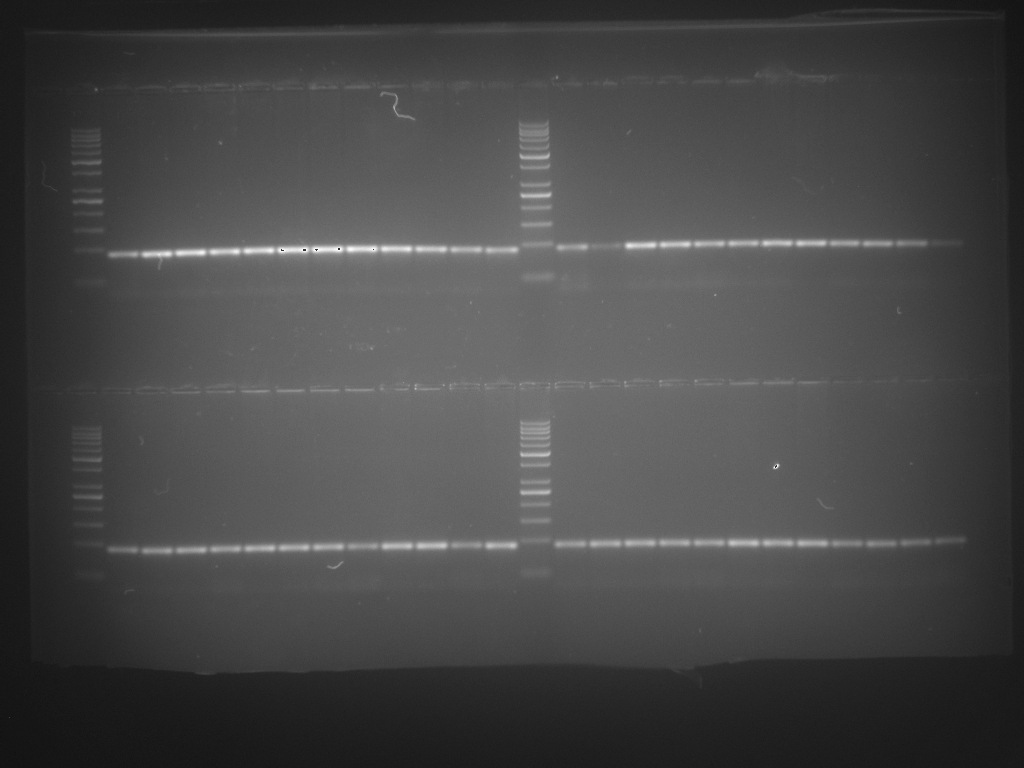

Supplement: S1 File — (ZIP) [file pone.0260246.s002.zip › 4. sswr934 212020 lane 1 and 2.jpg]

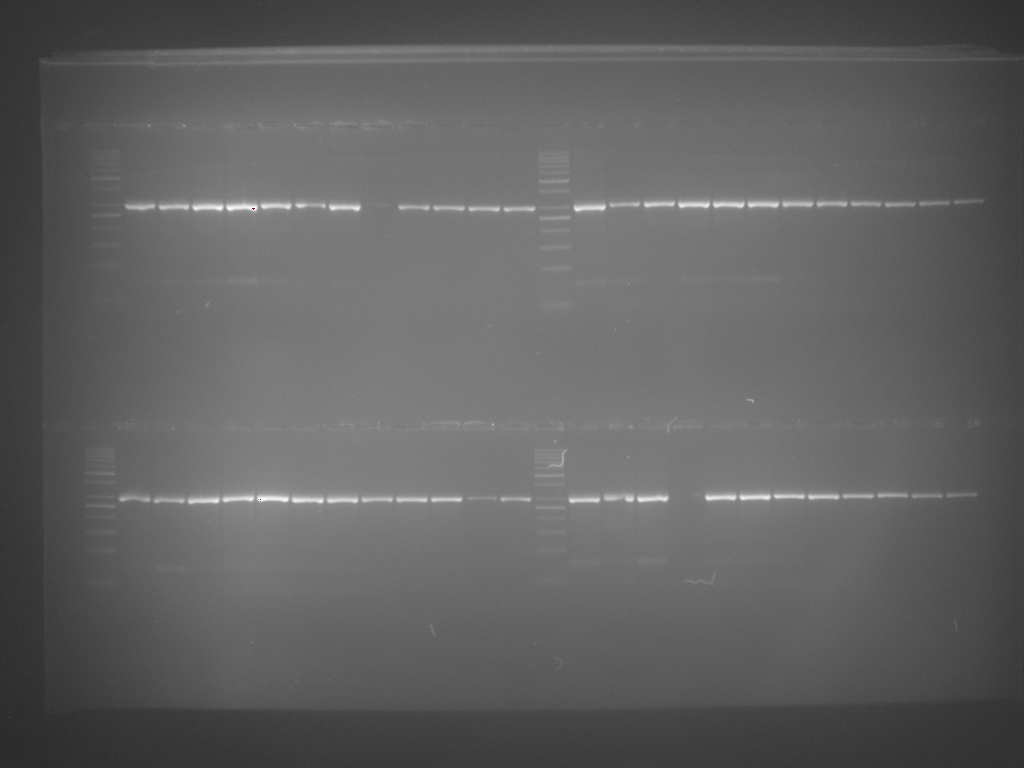

Supplement: S1 File — (ZIP) [file pone.0260246.s002.zip › 5a. BOESSR 702 2.1.20 (1 AND 2).jpg]

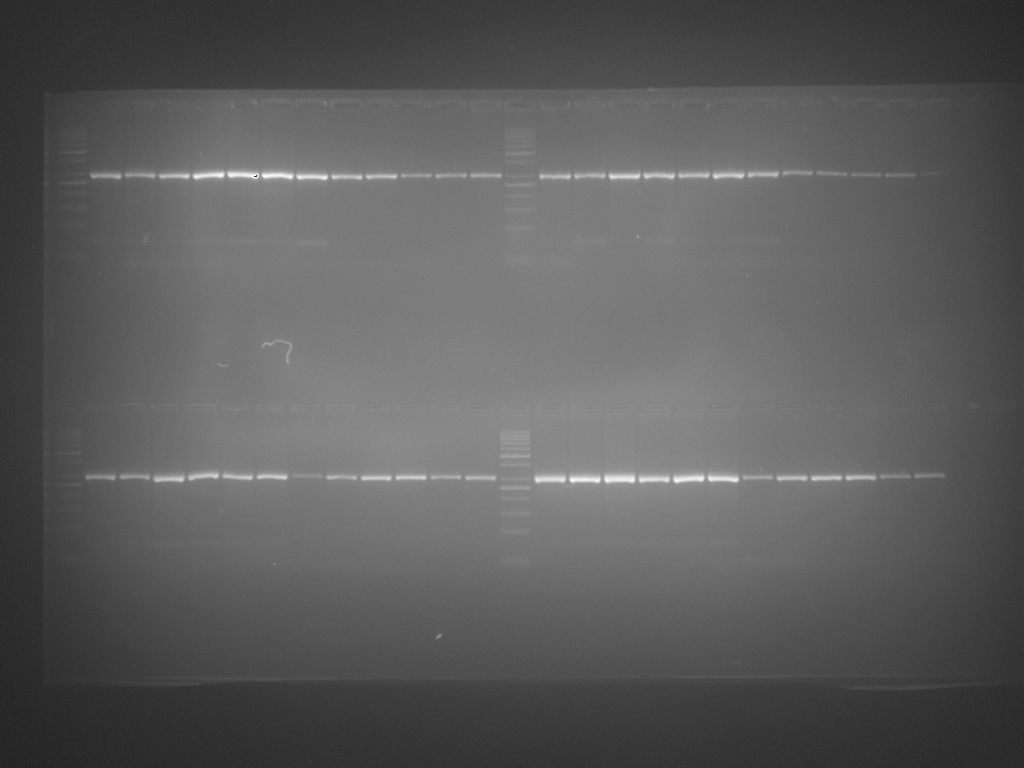

Supplement: S1 File — (ZIP) [file pone.0260246.s002.zip › 5b. BOESSR 702 (3,4).jpg]

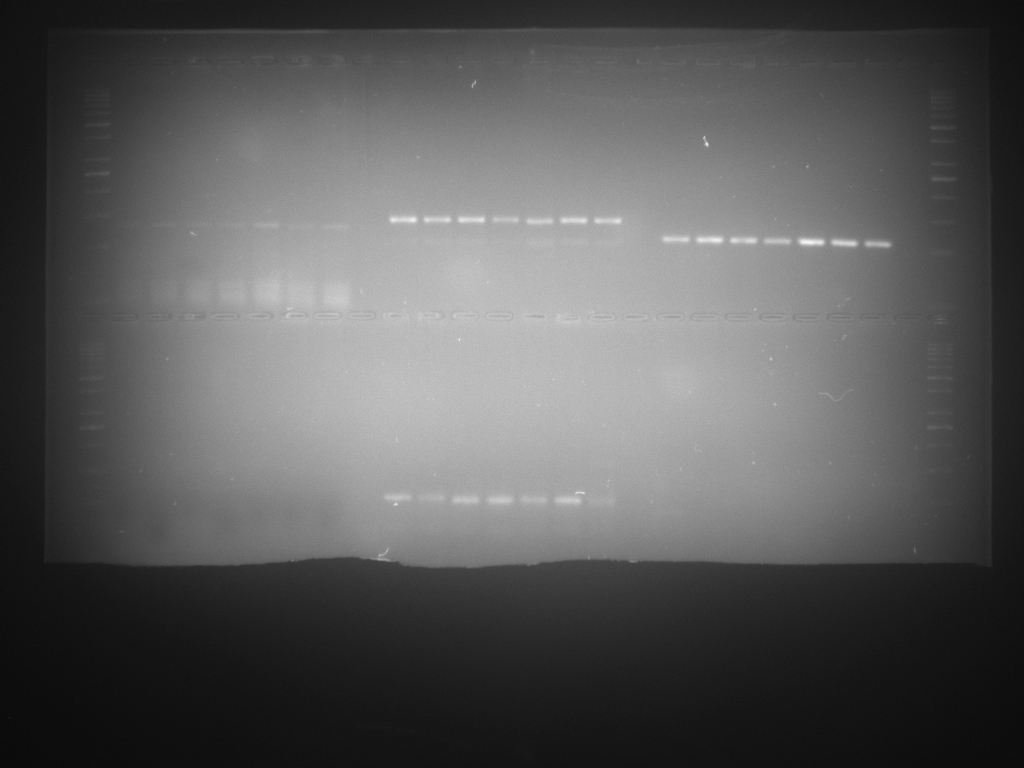

Supplement: S1 File — (ZIP) [file pone.0260246.s002.zip › 06,21,26,80,83,85.jpg]

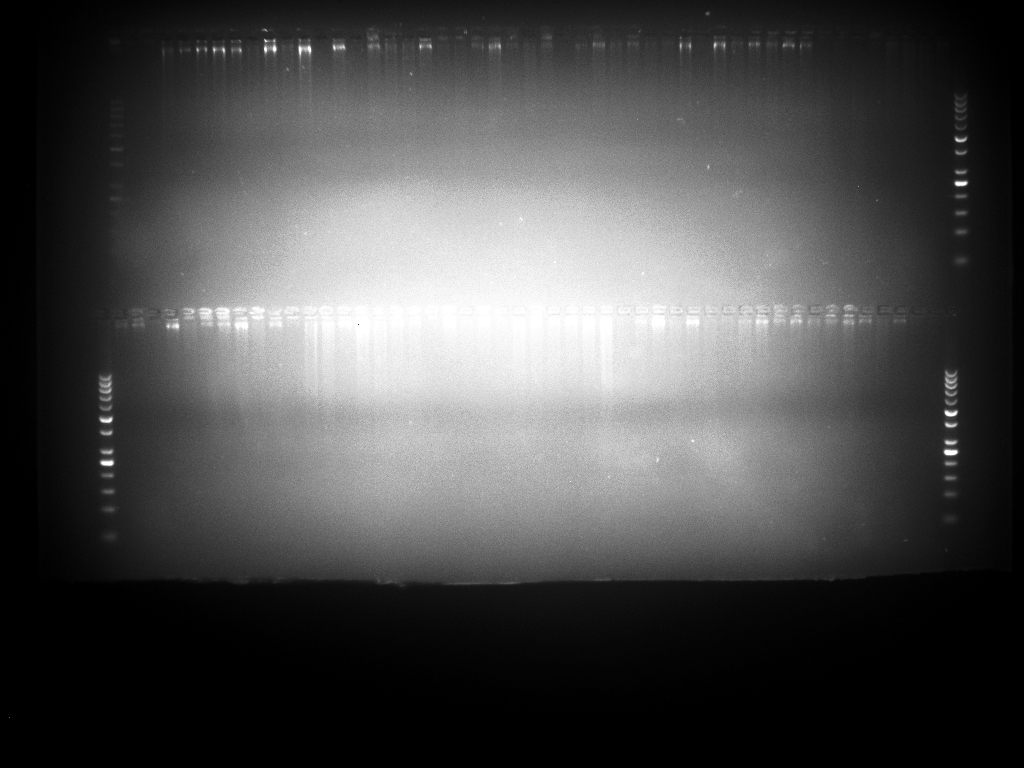

Supplement: S1 File — (ZIP) [file pone.0260246.s002.zip › 6-2-2020 MORIBG.jpg]

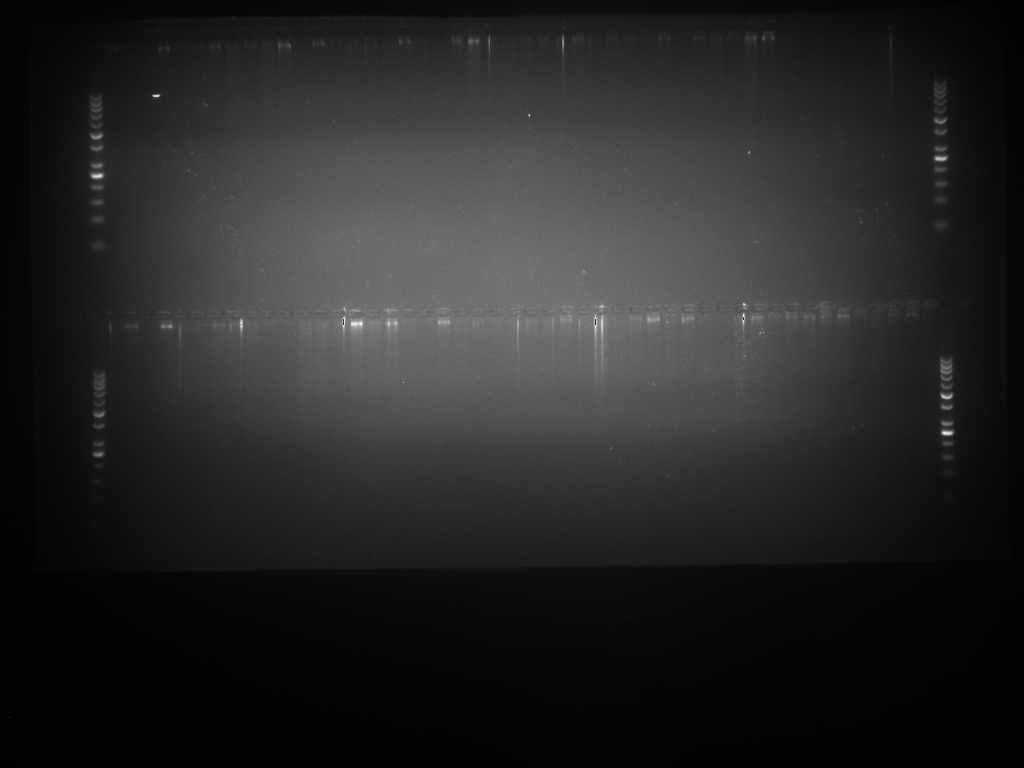

Supplement: S1 File — (ZIP) [file pone.0260246.s002.zip › 6-2-2020 MORIG 2.jpg]

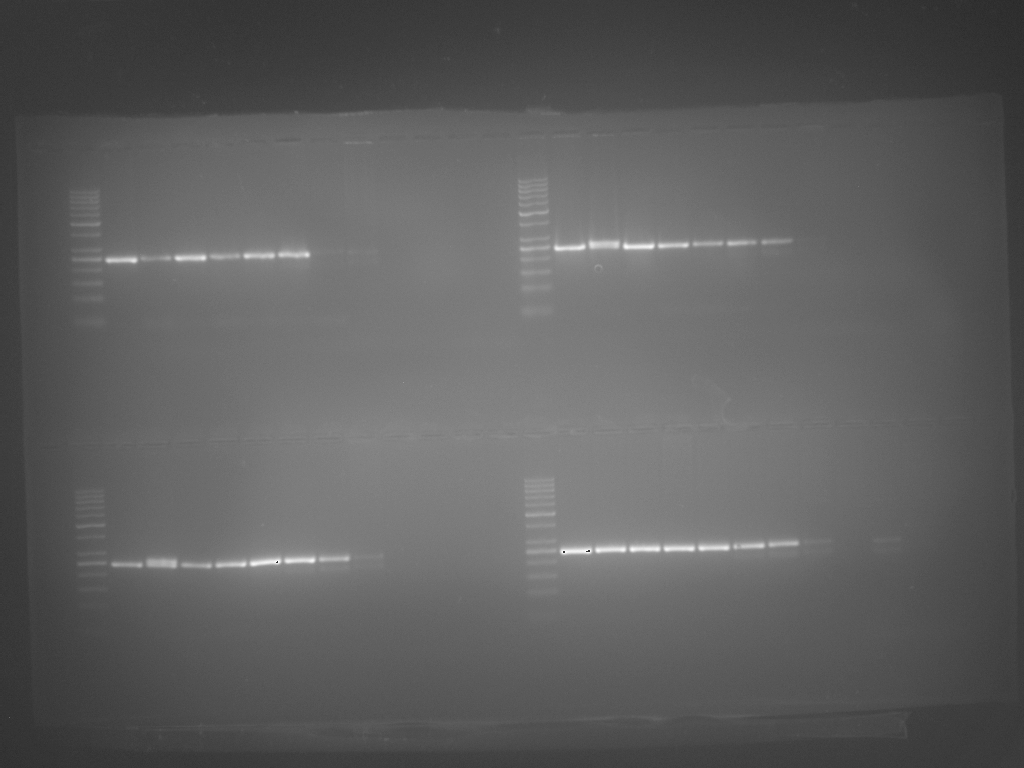

Supplement: S1 File — (ZIP) [file pone.0260246.s002.zip › 6b. ssr145 1.1.2020 lane 3 and 4.jpg]

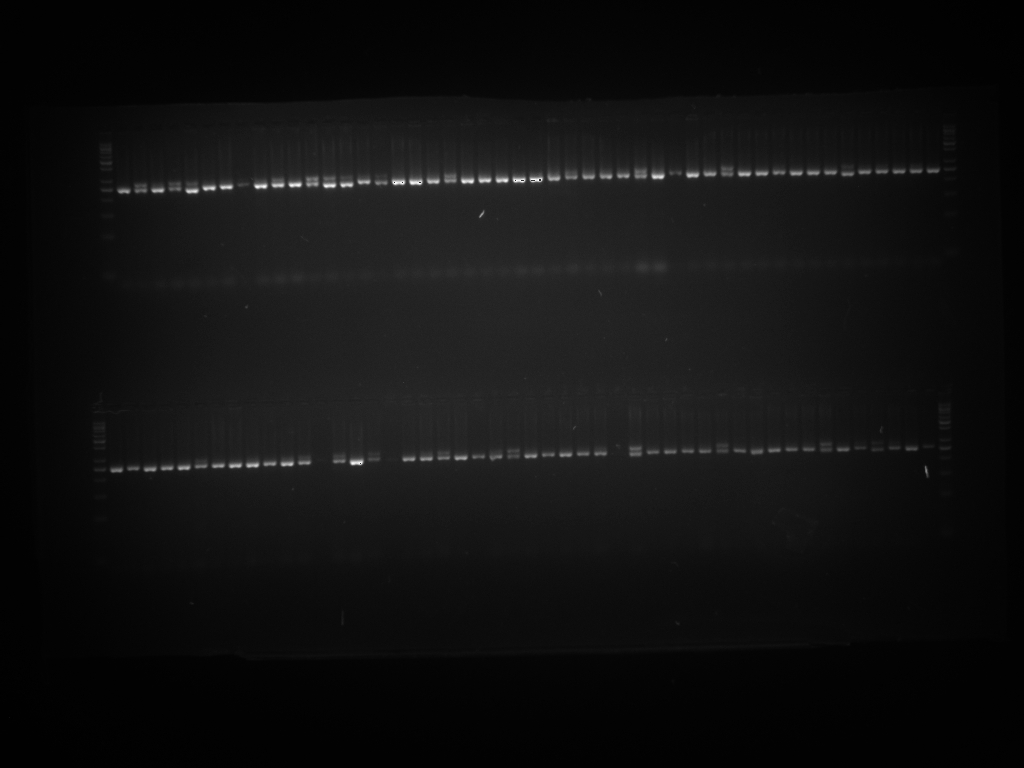

Supplement: S1 File — (ZIP) [file pone.0260246.s002.zip › 9. lane 1 and 2 ssr 303 512020.inf.jpg]

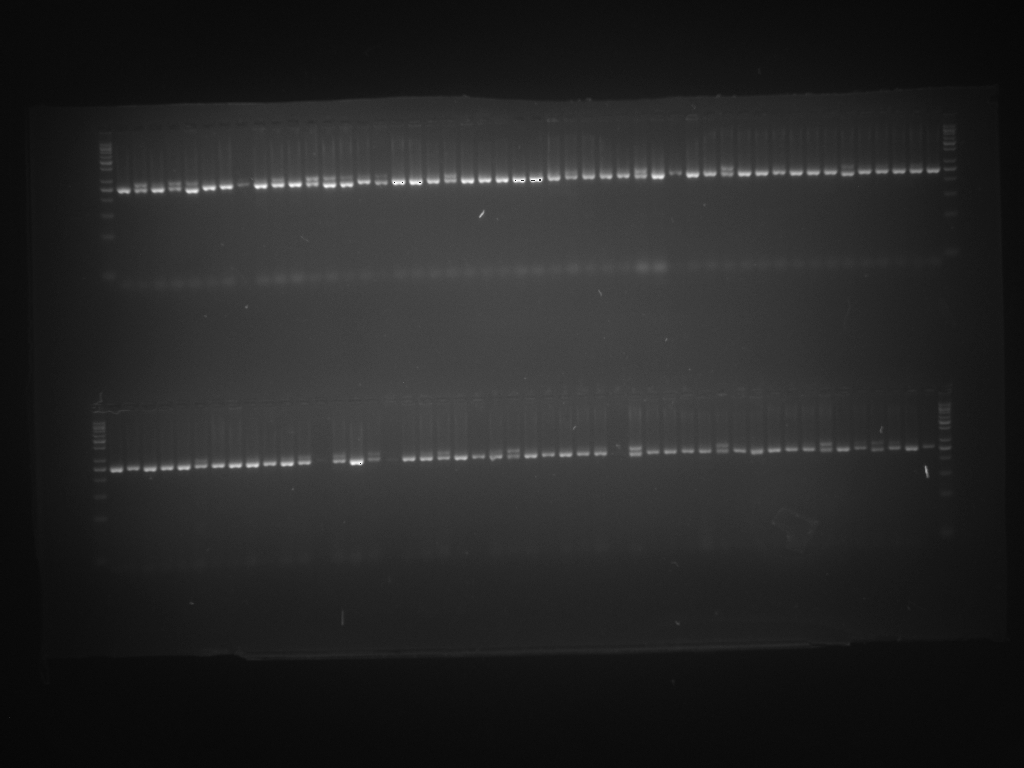

Supplement: S1 File — (ZIP) [file pone.0260246.s002.zip › 10. ssr 251 lane 3 and 4 512020.jpg]

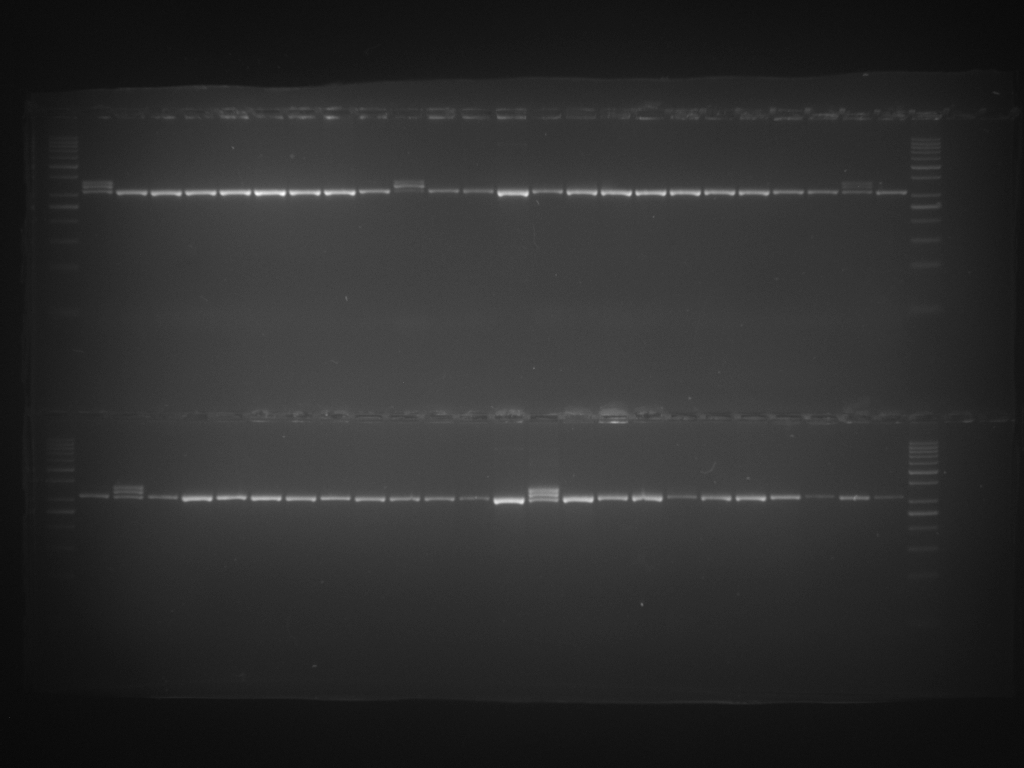

Supplement: S1 File — (ZIP) [file pone.0260246.s002.zip › 11b. lane 3 and 4 boessr333 412020.inf.jpg]

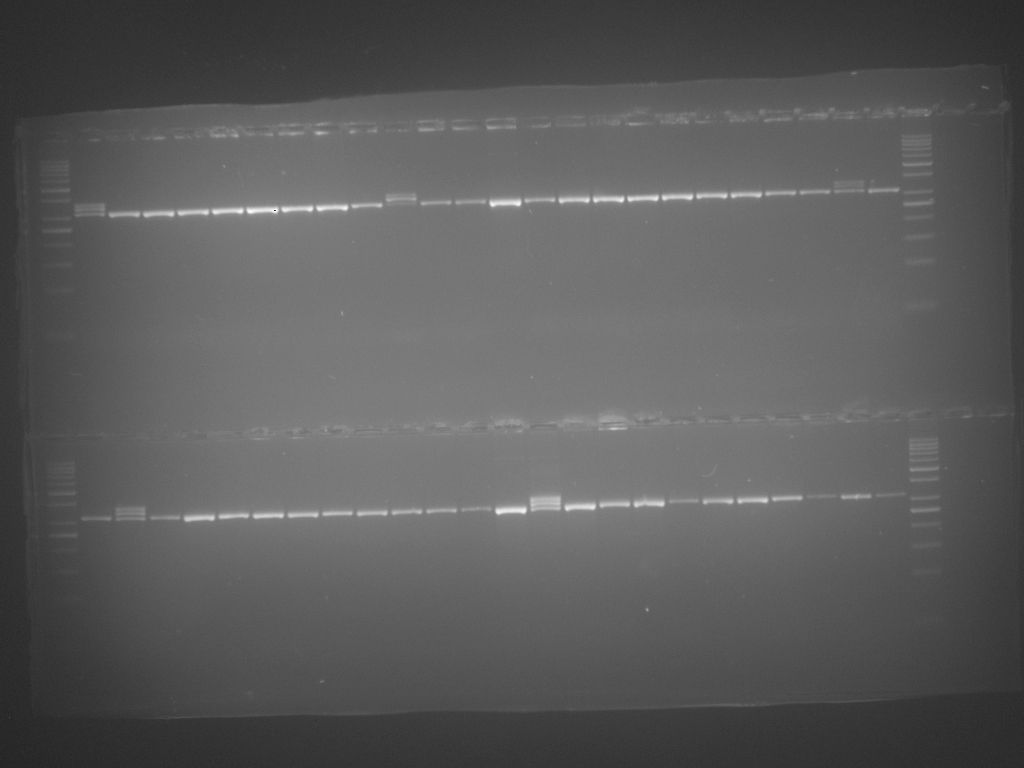

Supplement: S1 File — (ZIP) [file pone.0260246.s002.zip › 11b. lane 3 and 4 boessr333 412020.jpg]

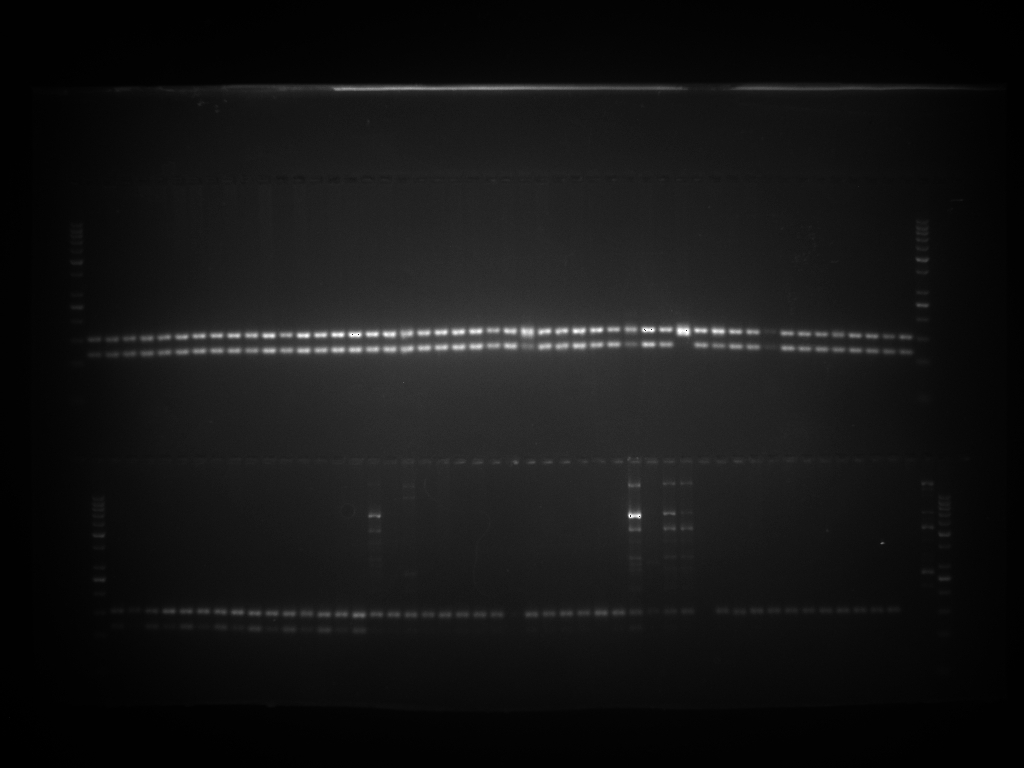

Supplement: S1 File — (ZIP) [file pone.0260246.s002.zip › 13.. boessr 343 lane 1 and 2 1712020.jpg]

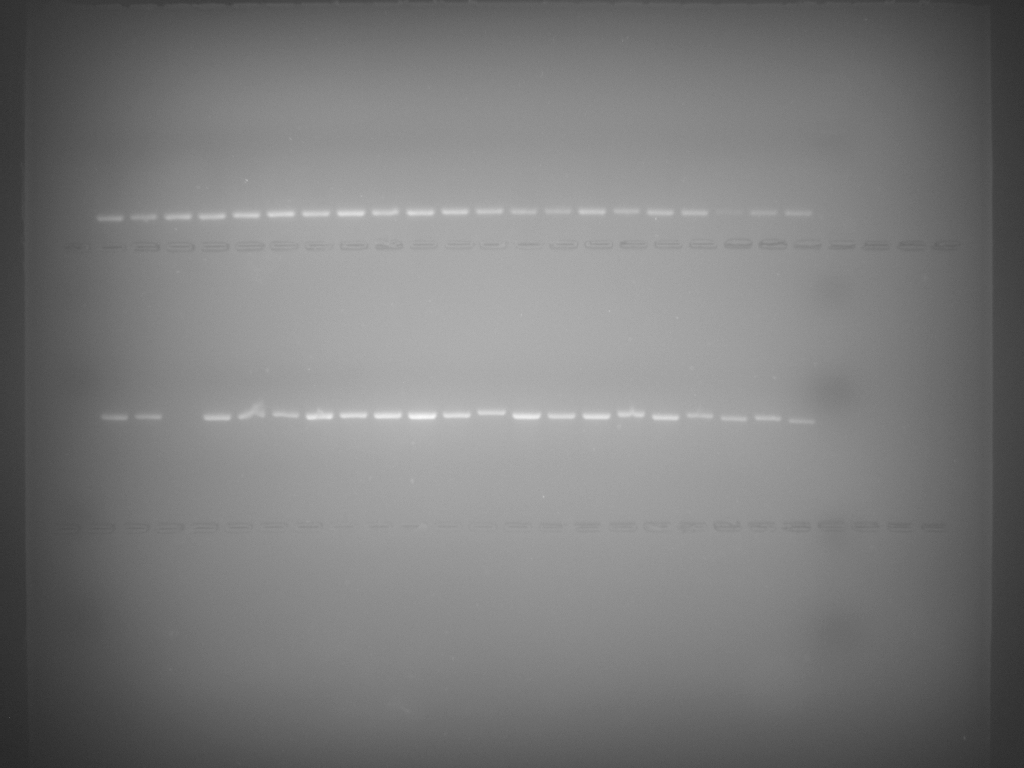

Supplement: S1 File — (ZIP) [file pone.0260246.s002.zip › 15. ND 20 27.1.20.jpg]

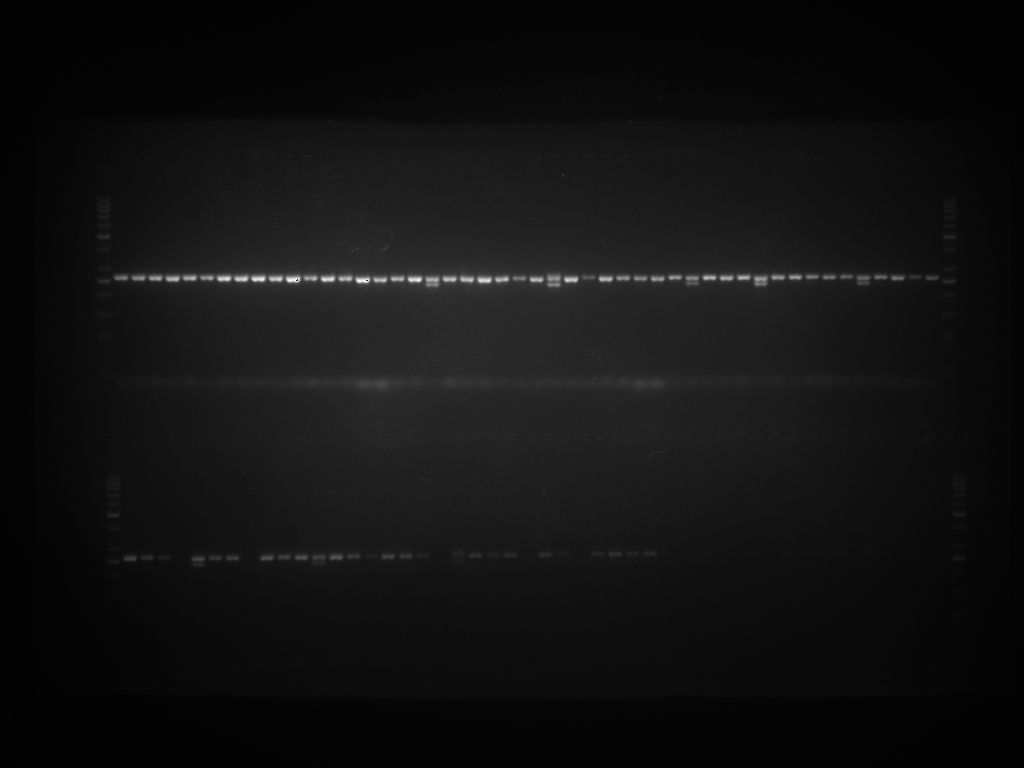

Supplement: S1 File — (ZIP) [file pone.0260246.s002.zip › 17. bosf 1613 lane 3 and 4 1712020.jpg]

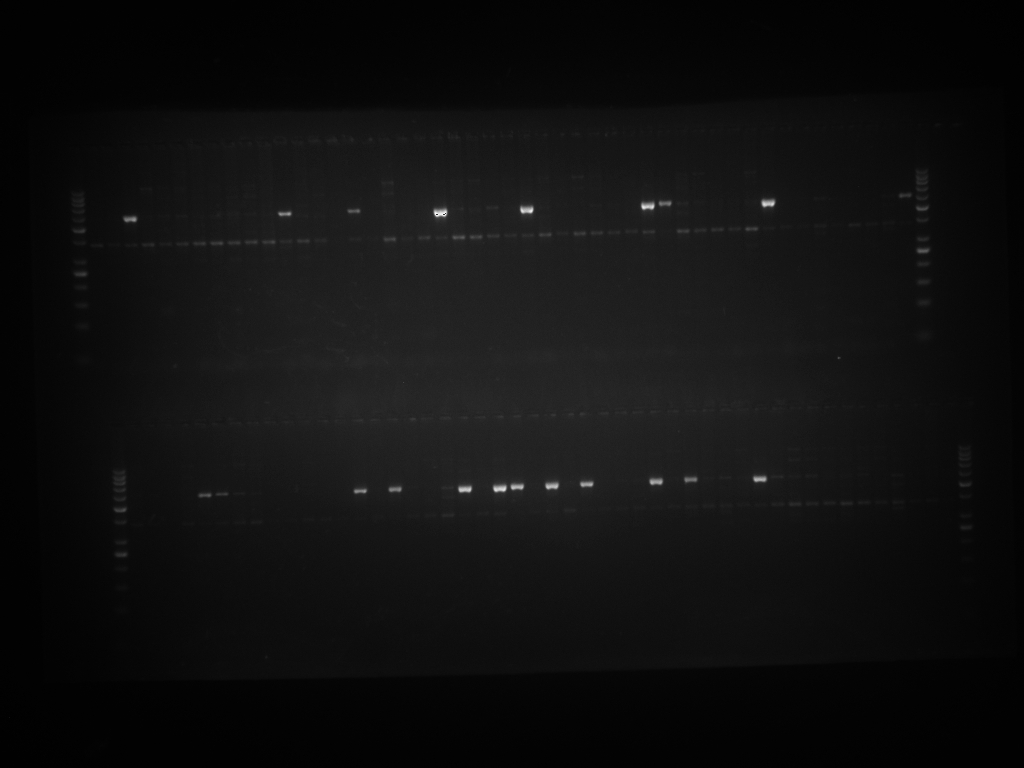

Supplement: S1 File — (ZIP) [file pone.0260246.s002.zip › 19. BOSF1163 1712020 LANE 3 AND 4.jpg]

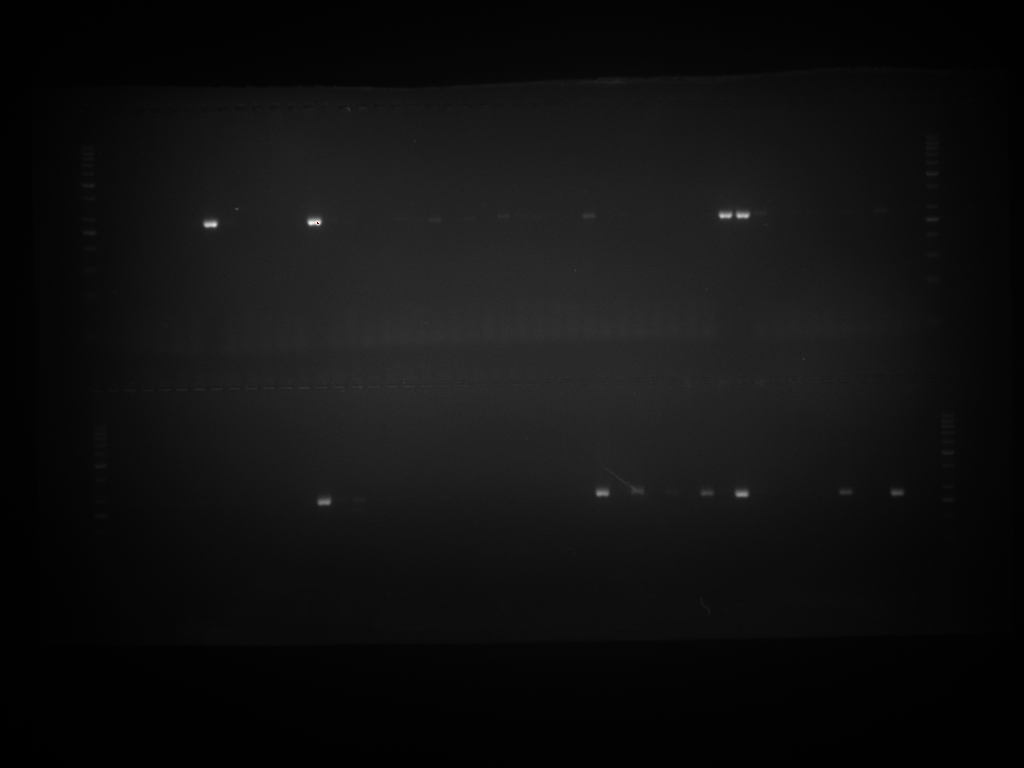

Supplement: S1 File — (ZIP) [file pone.0260246.s002.zip › 20. BOSF1103 lane 3 and 4 1812020.jpg]

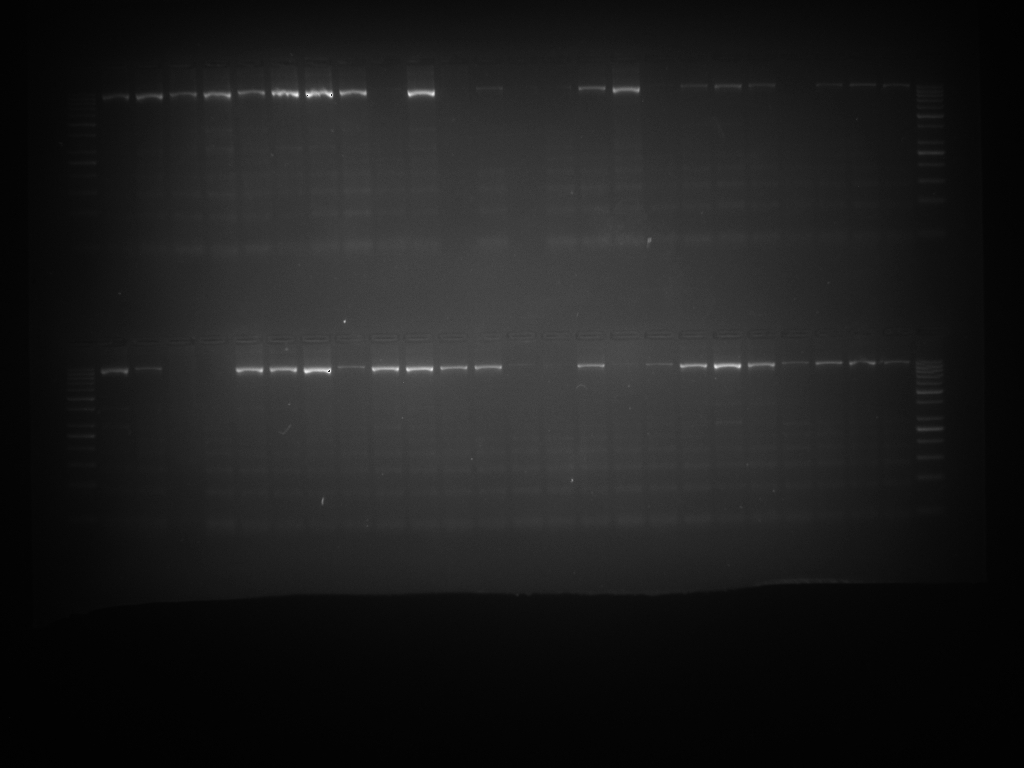

Supplement: S1 File — (ZIP) [file pone.0260246.s002.zip › 20-2-2020 mMYB B LANE 1 AND 2.jpg]

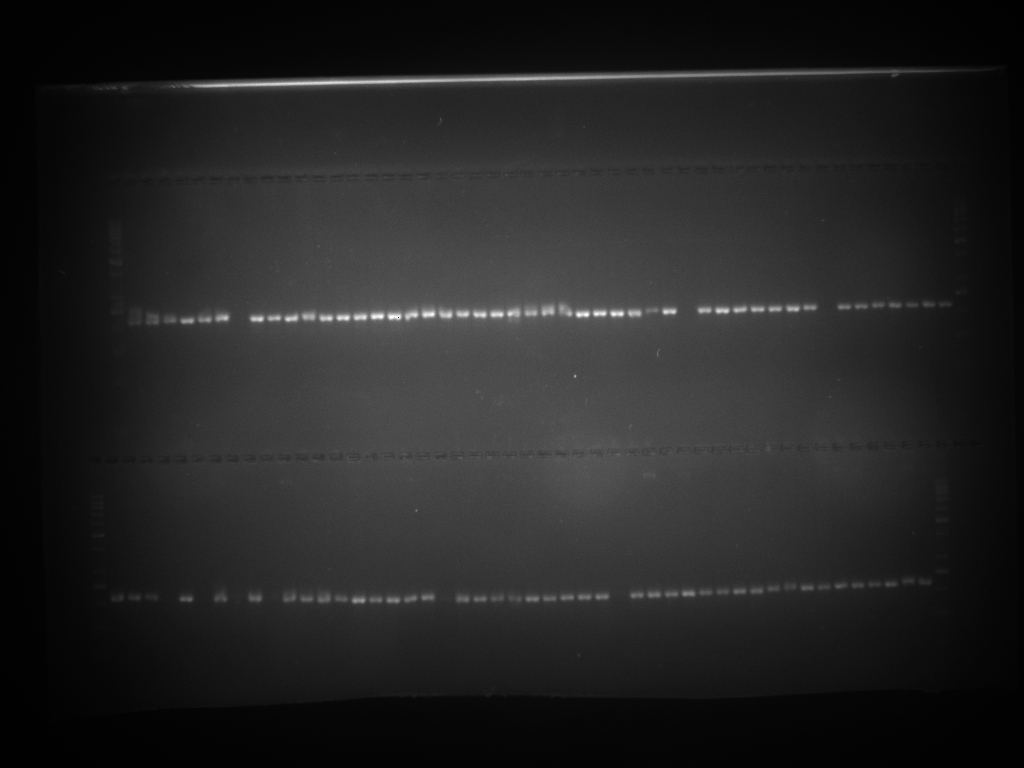

Supplement: S1 File — (ZIP) [file pone.0260246.s002.zip › 22. FITO 348 LANE 1 AND 2 2012020.jpg]

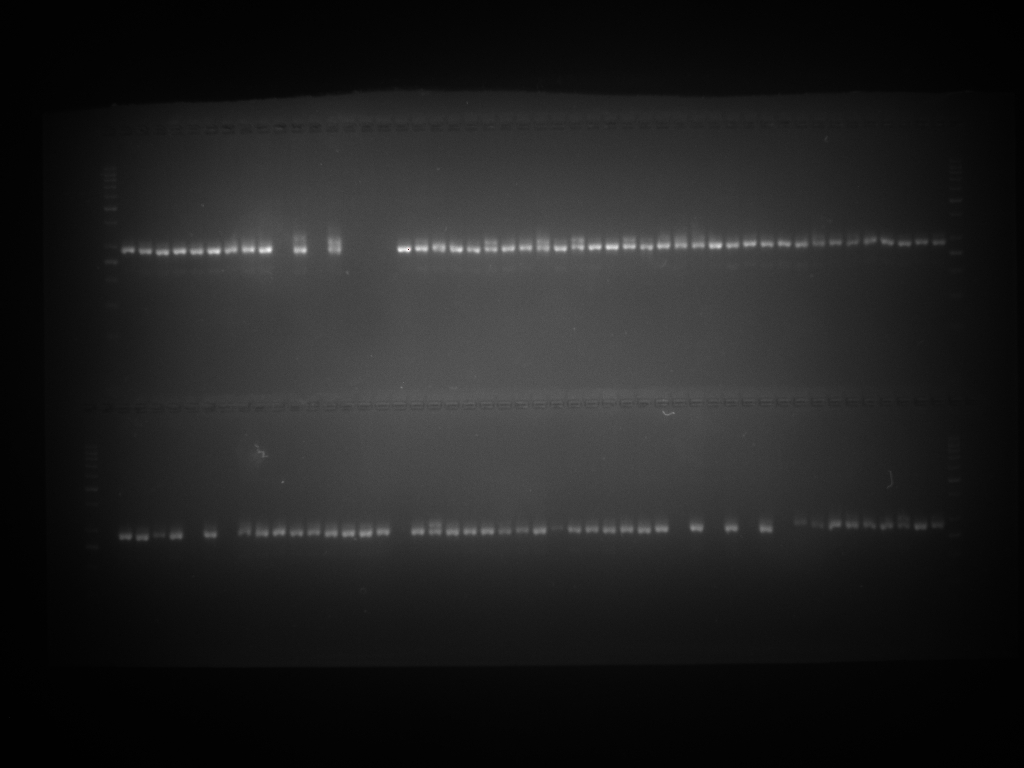

Supplement: S1 File — (ZIP) [file pone.0260246.s002.zip › 23.BOSF 2212 LANE3 AND 4 2012020 - Copy.jpg]

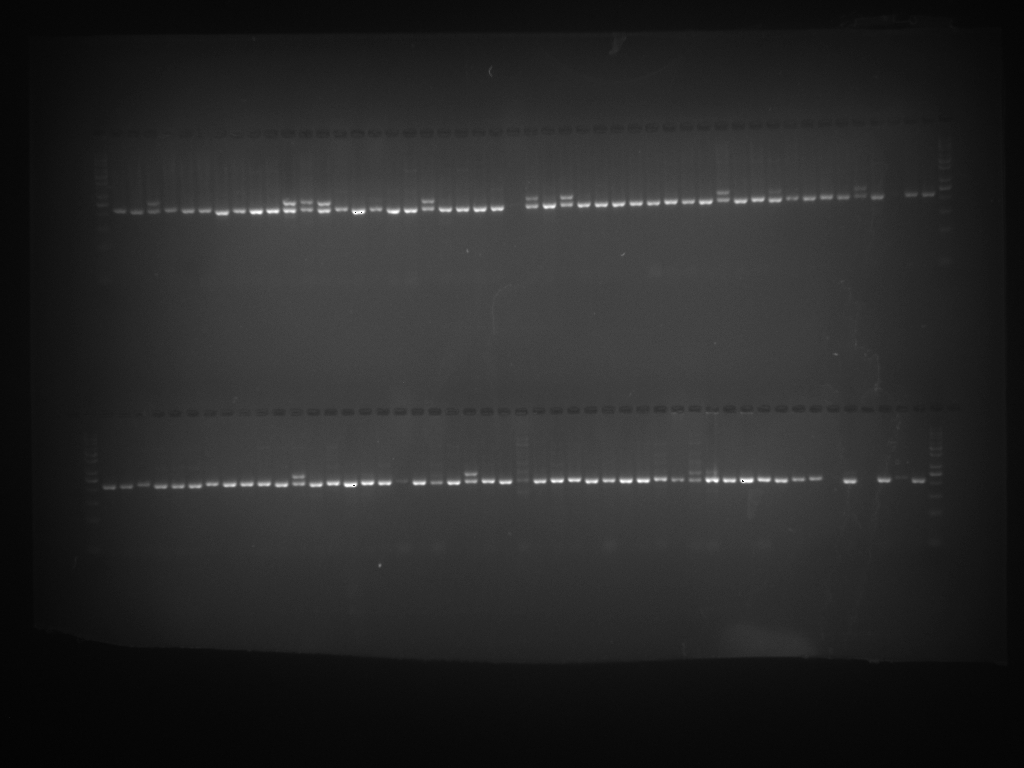

Supplement: S1 File — (ZIP) [file pone.0260246.s002.zip › 24. BOPM14 2012020 LANE 1 AND 2.jpg]

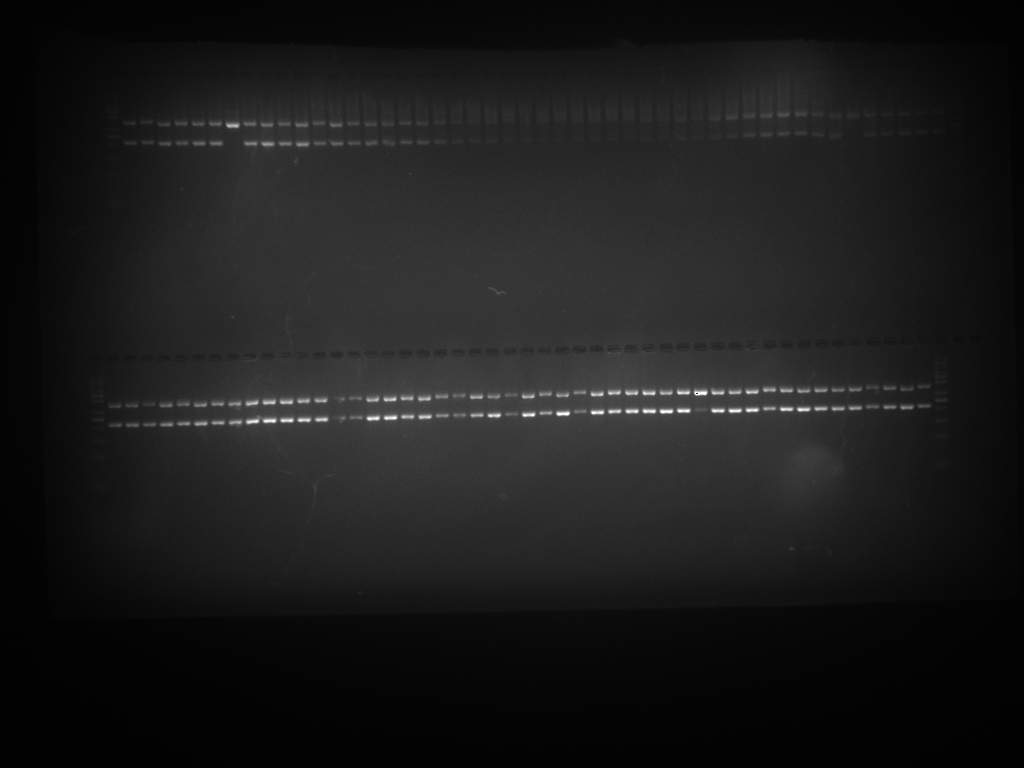

Supplement: S1 File — (ZIP) [file pone.0260246.s002.zip › 25. BOPM 16 2012020 LANE 3 AND 4.jpg]

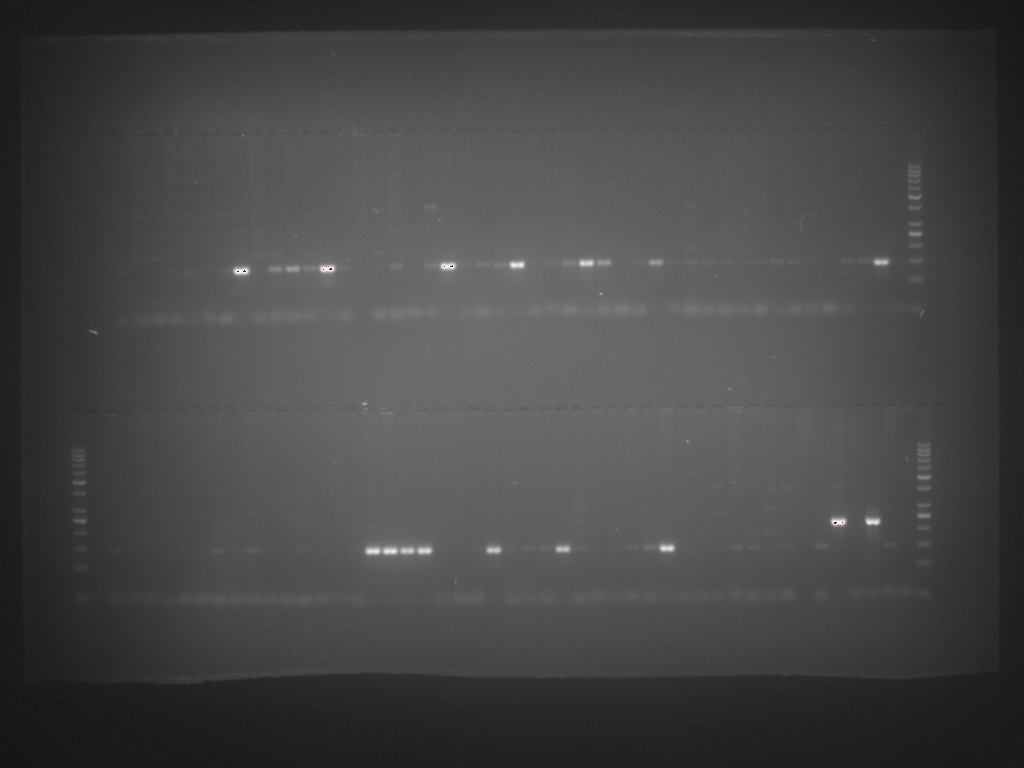

Supplement: S1 File — (ZIP) [file pone.0260246.s002.zip › 26.bopm15 2212020 lane 1 and 2.jpg]

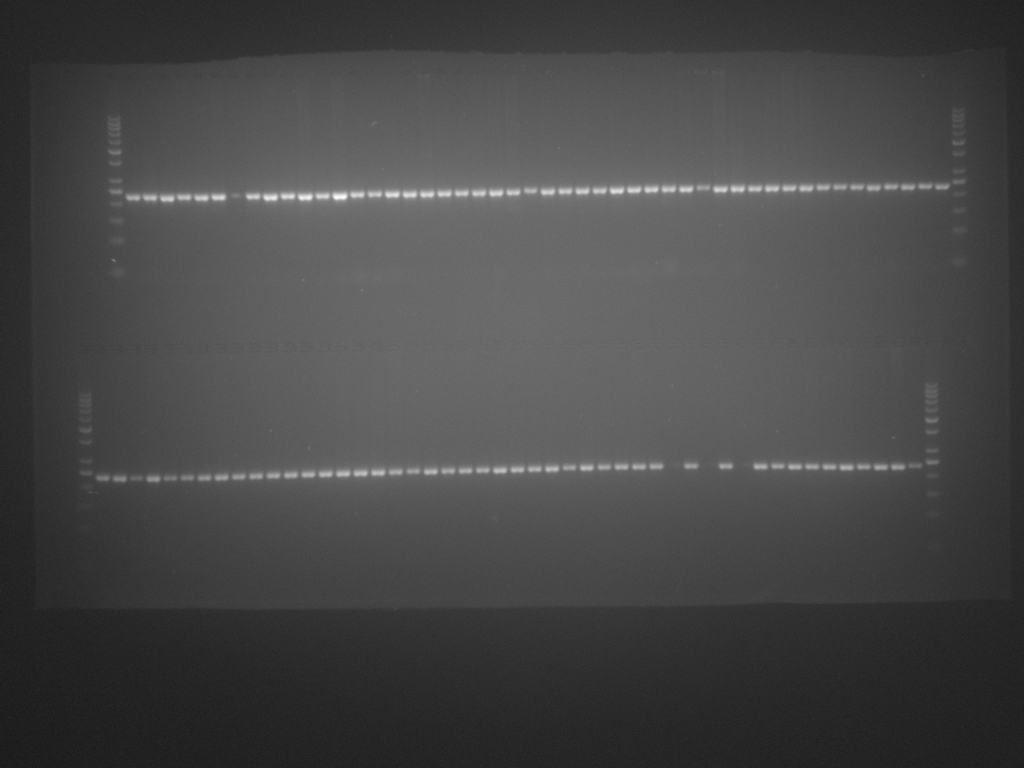

Supplement: S1 File — (ZIP) [file pone.0260246.s002.zip › 27. BOPM 21 LANE 3 AND 4 22-1-2020.jpg]

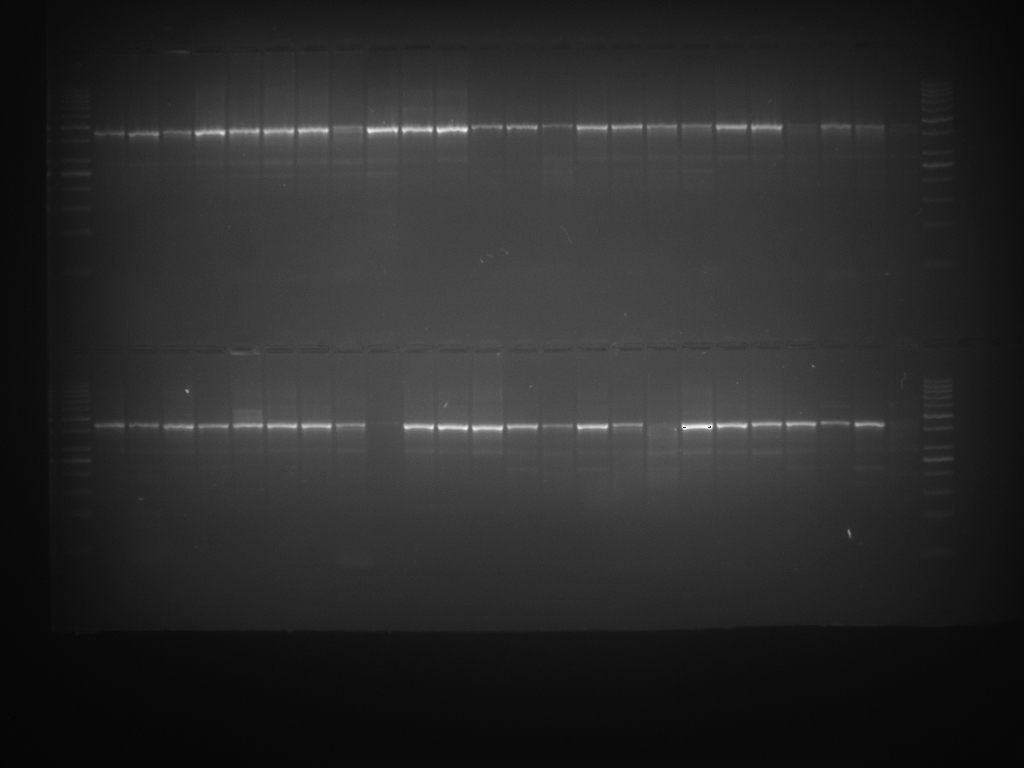

Supplement: S1 File — (ZIP) [file pone.0260246.s002.zip › 28. BOPM6 2312020 LANE 1 AND 2.jpg]

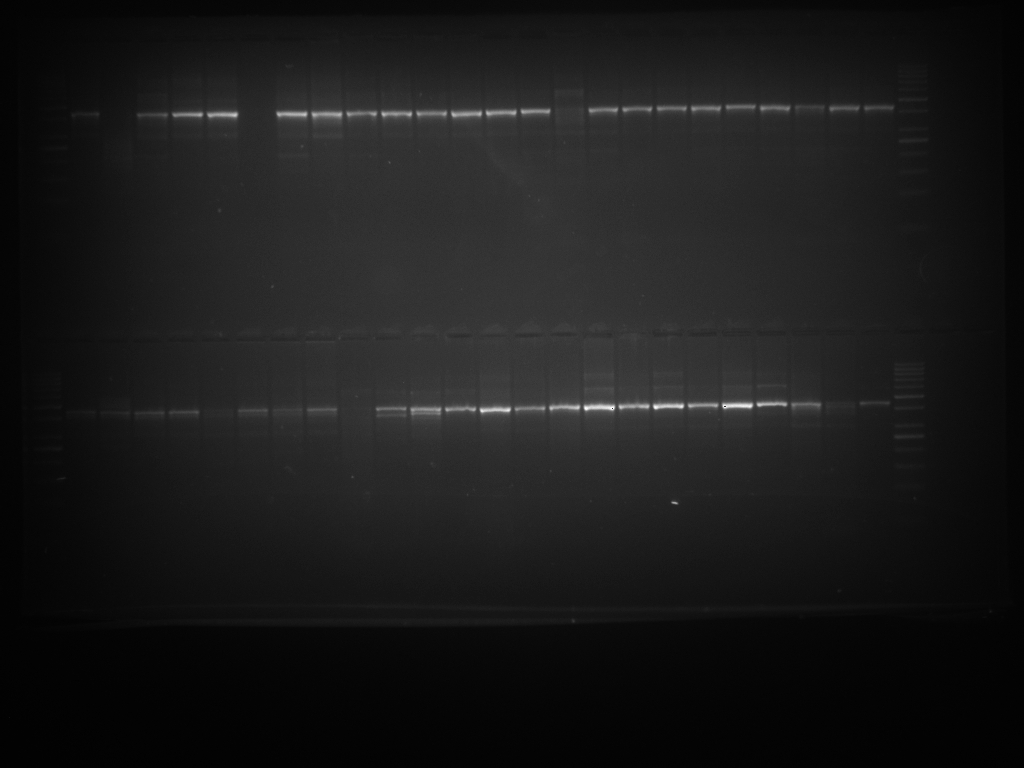

Supplement: S1 File — (ZIP) [file pone.0260246.s002.zip › 28. BOPM6 2312020 LANE 3 AND 4.jpg]

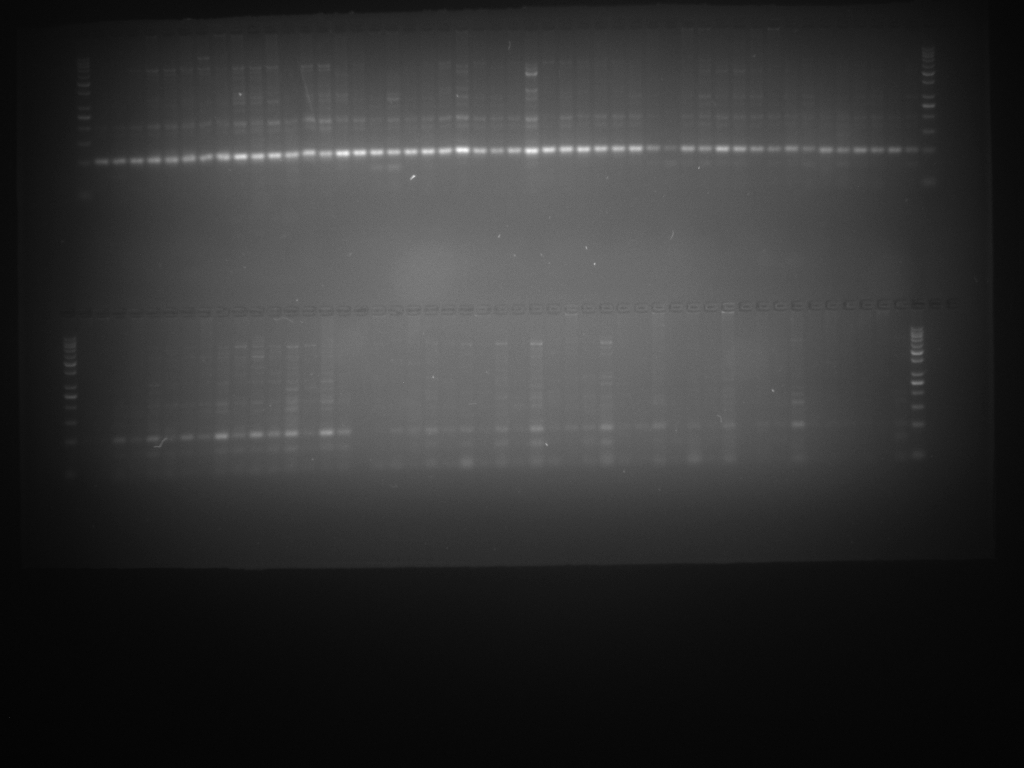

Supplement: S1 File — (ZIP) [file pone.0260246.s002.zip › 30.BOE 878 LANE 3 AND 4 2512020.jpg]

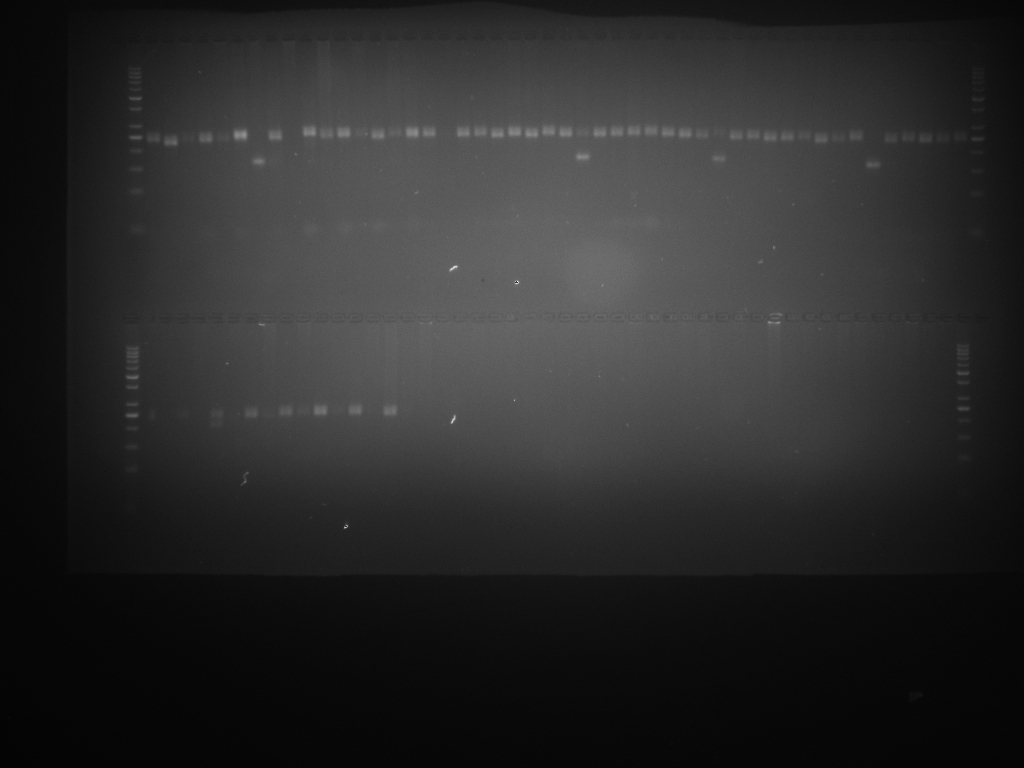

Supplement: S1 File — (ZIP) [file pone.0260246.s002.zip › 31. CNu 286 2412020 lan e 3 and 4.jpg]

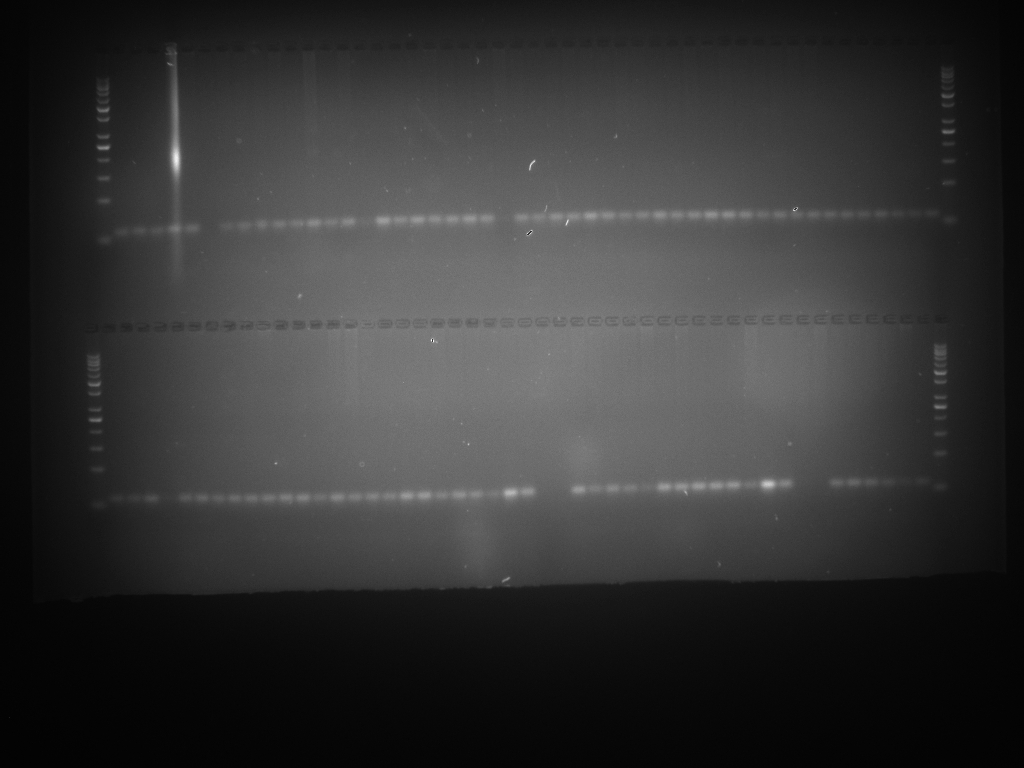

Supplement: S1 File — (ZIP) [file pone.0260246.s002.zip › 32. bosf 1637 2512020 lane 1 and 2.jpg]

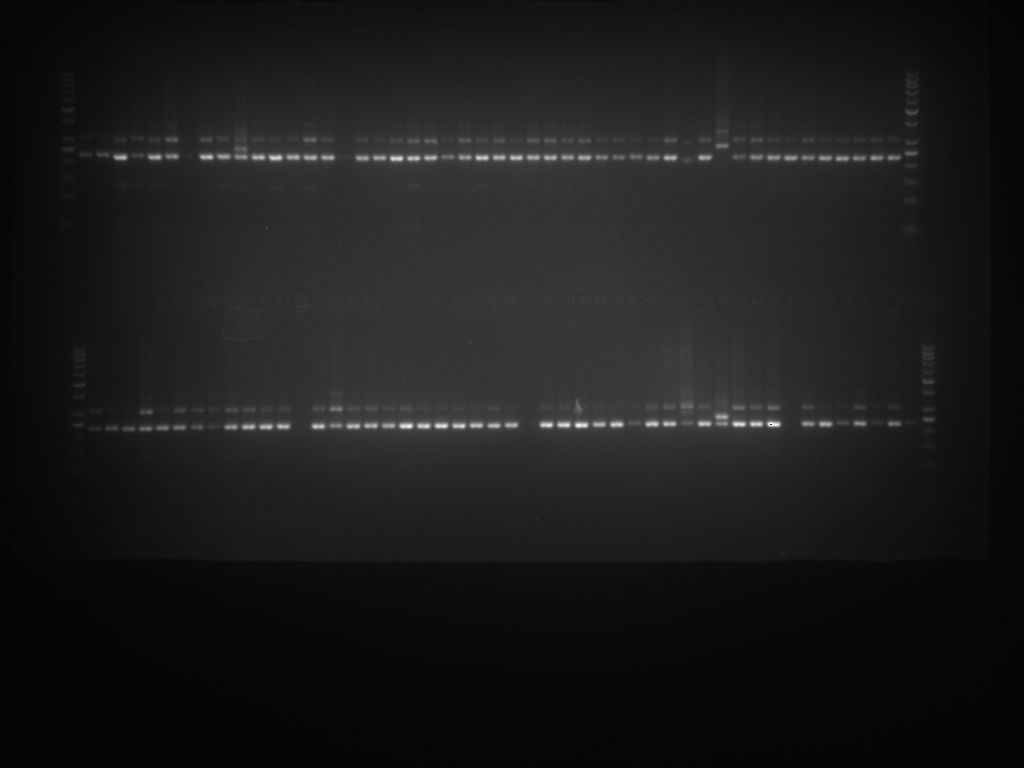

Supplement: S1 File — (ZIP) [file pone.0260246.s002.zip › 33.BOSF1537 LANE 3 AND 4 2712020.jpg]

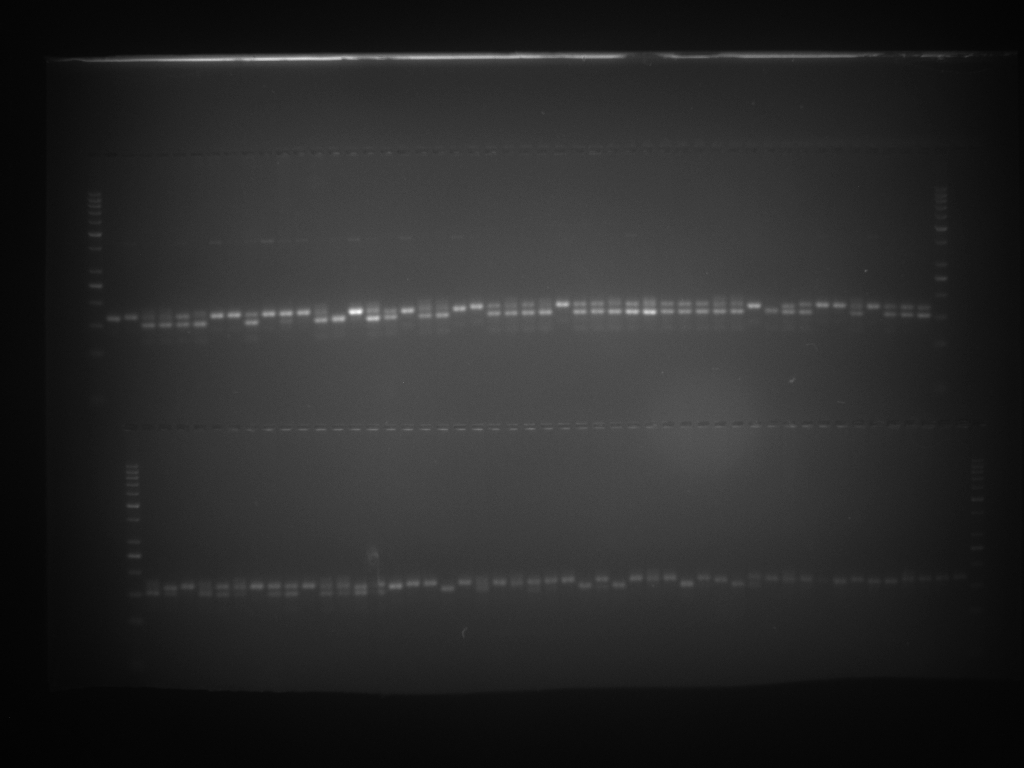

Supplement: S1 File — (ZIP) [file pone.0260246.s002.zip › 34. bosf 2717 lane 1 and 2 1812020.jpg]

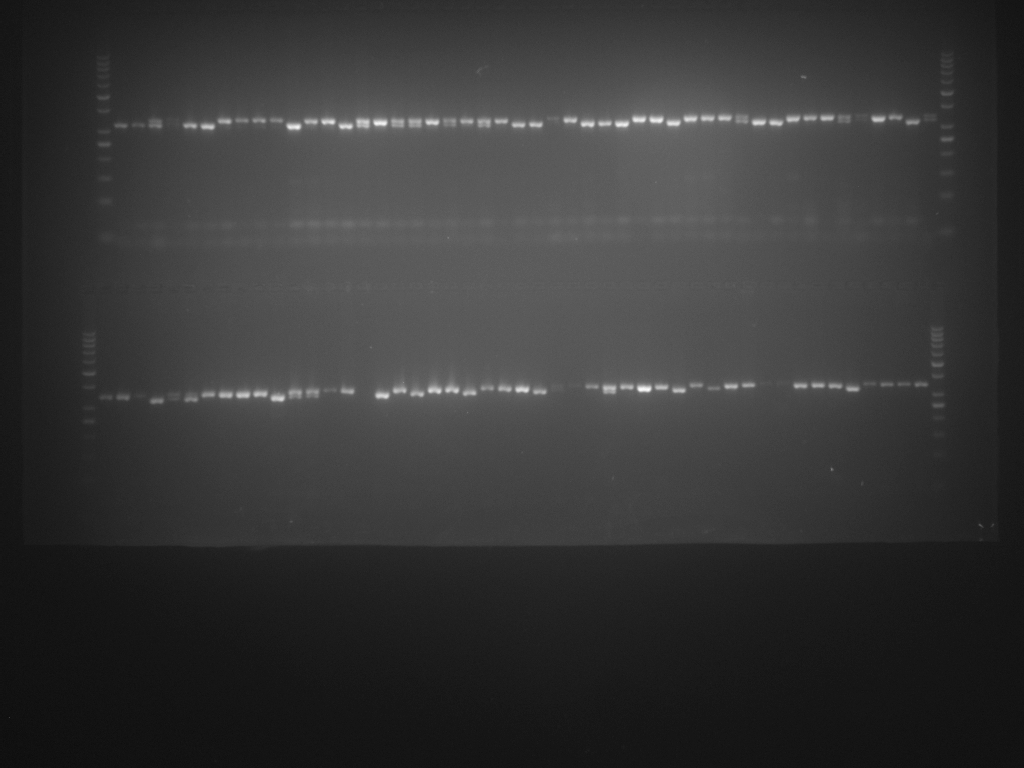

Supplement: S1 File — (ZIP) [file pone.0260246.s002.zip › 35. BOGMS0692 LAE 3 AND 4 27-1-2020.jpg]

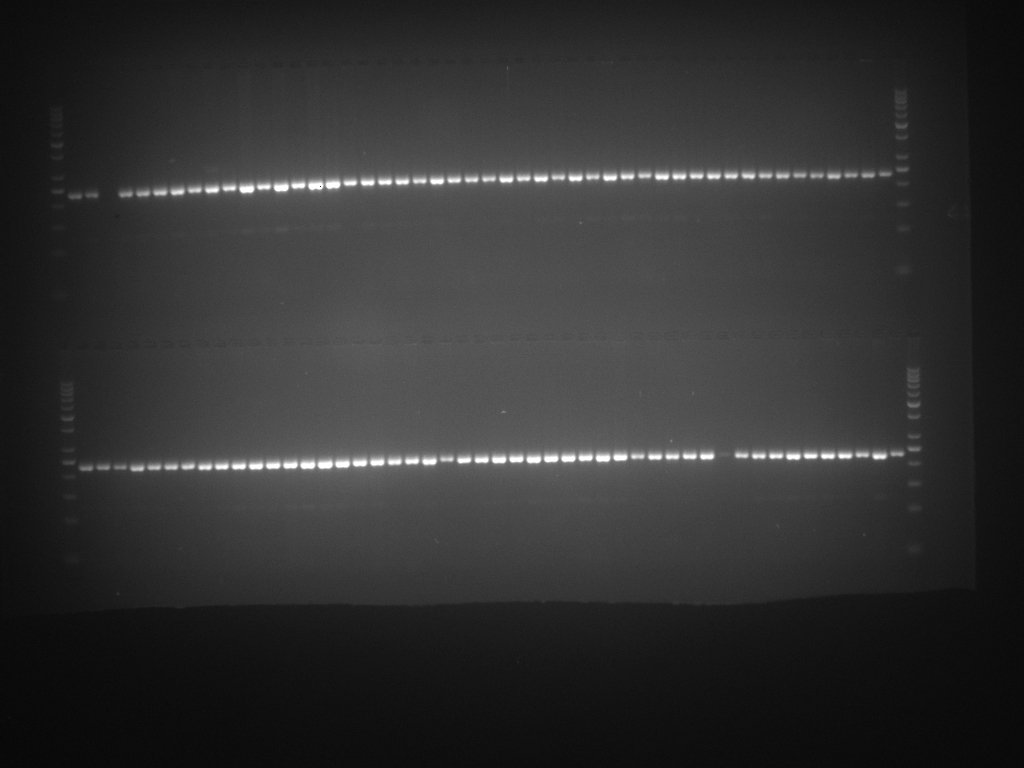

Supplement: S1 File — (ZIP) [file pone.0260246.s002.zip › 36. BOGMS0510 LANE 1 AND 2 2512020.jpg]

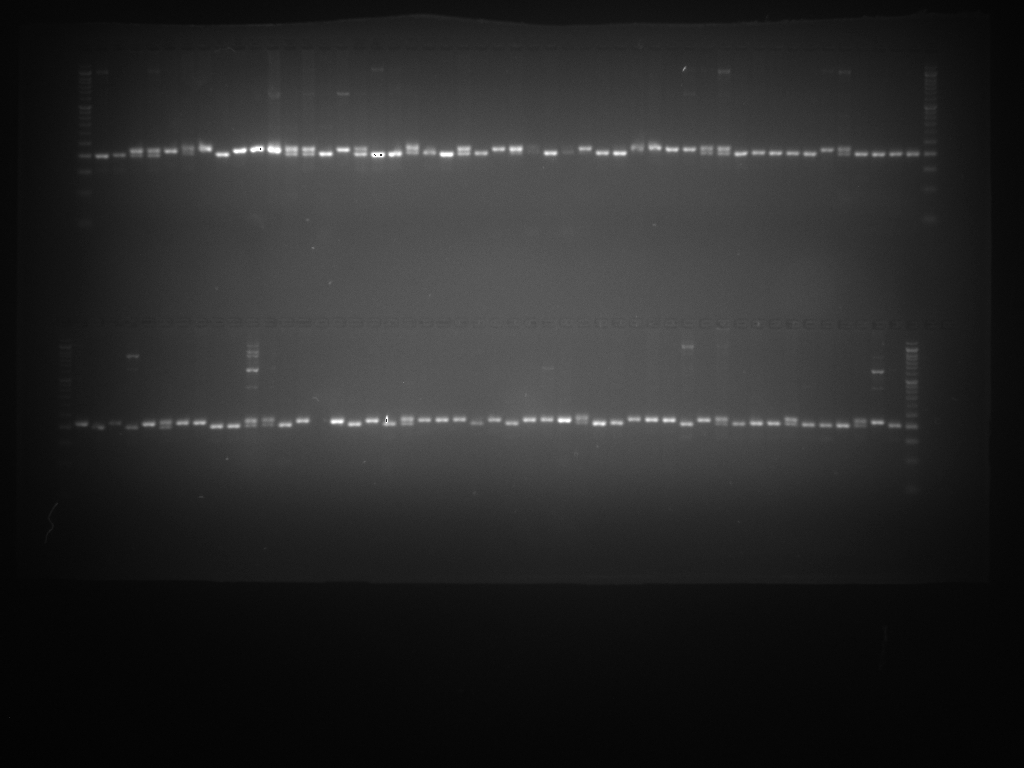

Supplement: S1 File — (ZIP) [file pone.0260246.s002.zip › 37.BOGMS1164 LANE 3 AND 4 2812020.jpg]

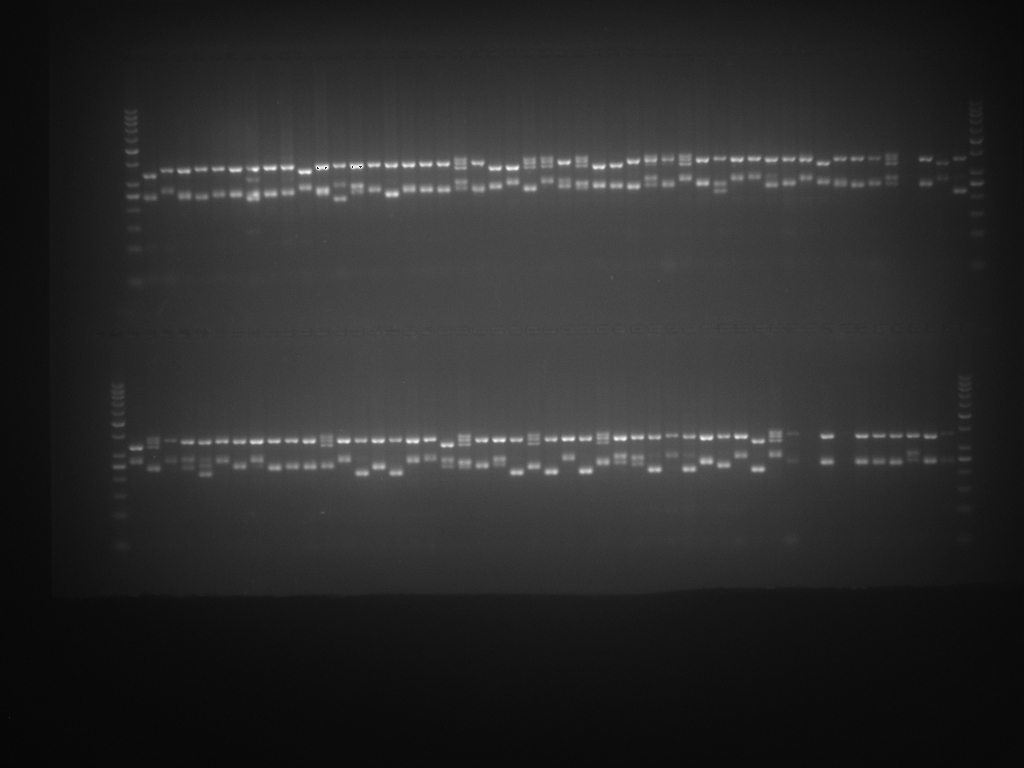

Supplement: S1 File — (ZIP) [file pone.0260246.s002.zip › 38. BOGMS 0742 LANE 1 AND 2 2712020 - Copy.jpg]

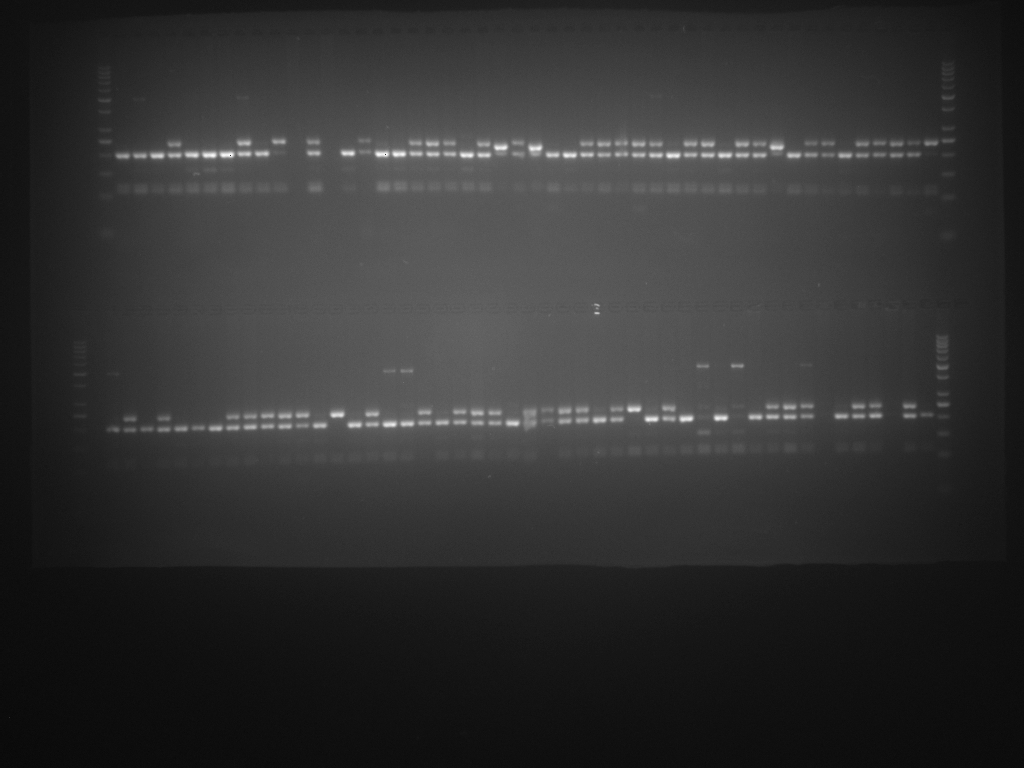

Supplement: S1 File — (ZIP) [file pone.0260246.s002.zip › 39. BOGMS 0929 LANE 3 AND 4 2512020.jpg]

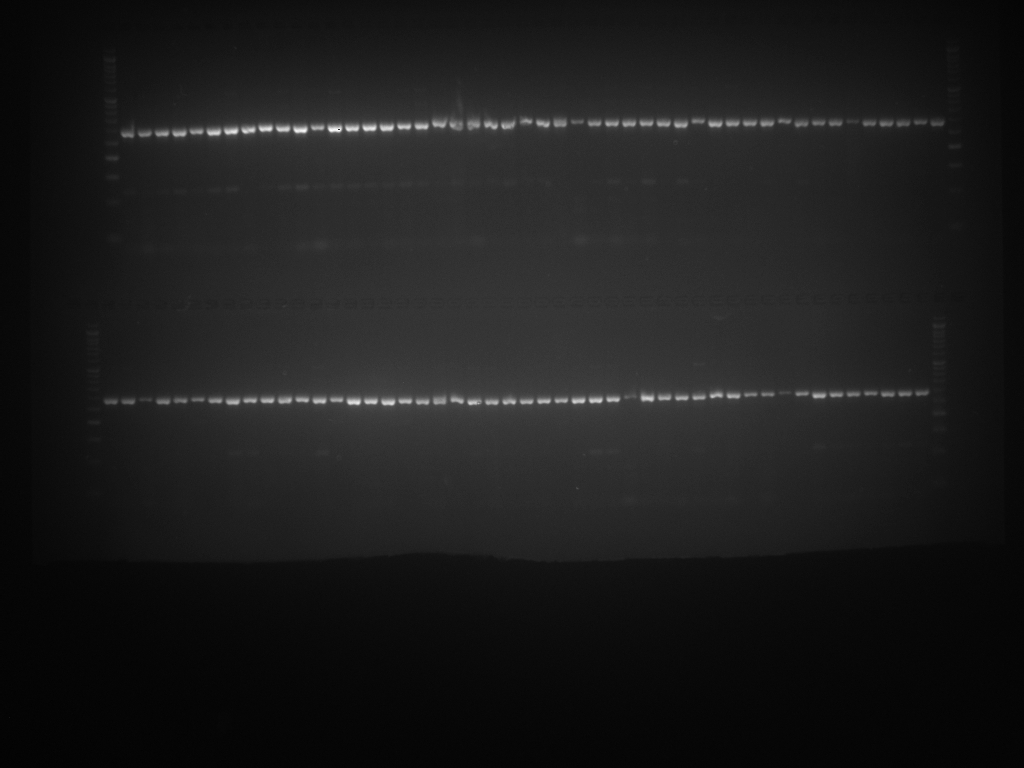

Supplement: S1 File — (ZIP) [file pone.0260246.s002.zip › 44.BOGMS 1465 2712020 LANE 1 AND 2.jpg]

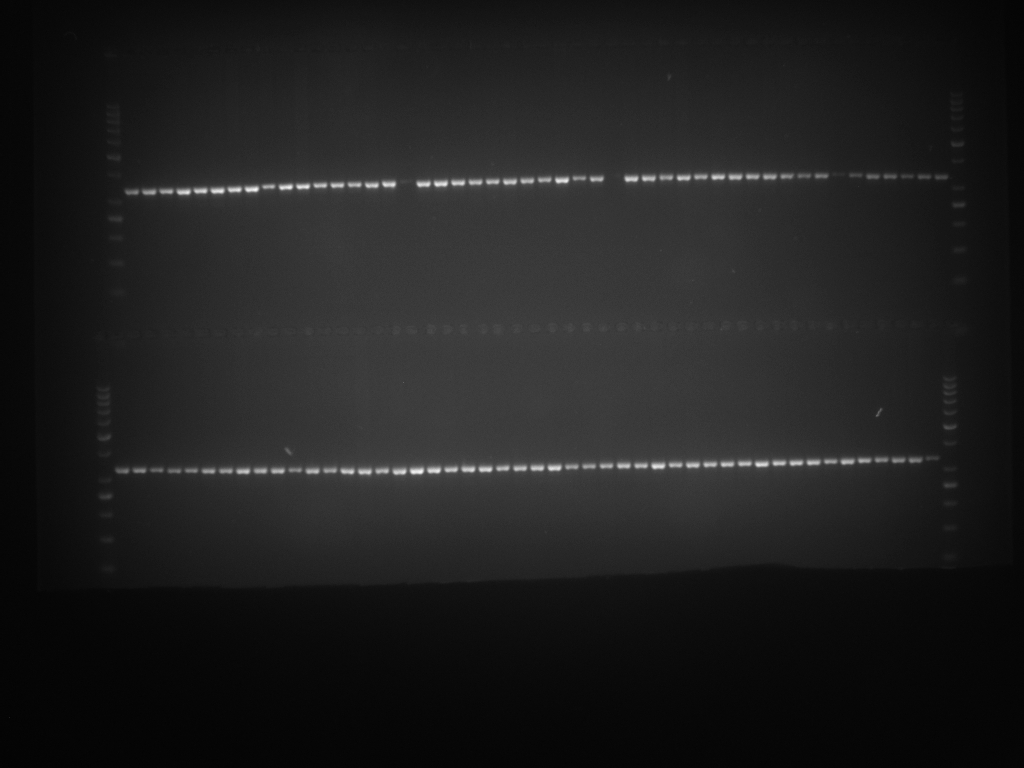

Supplement: S1 File — (ZIP) [file pone.0260246.s002.zip › BNGMS 490 3-2-2020.jpg]

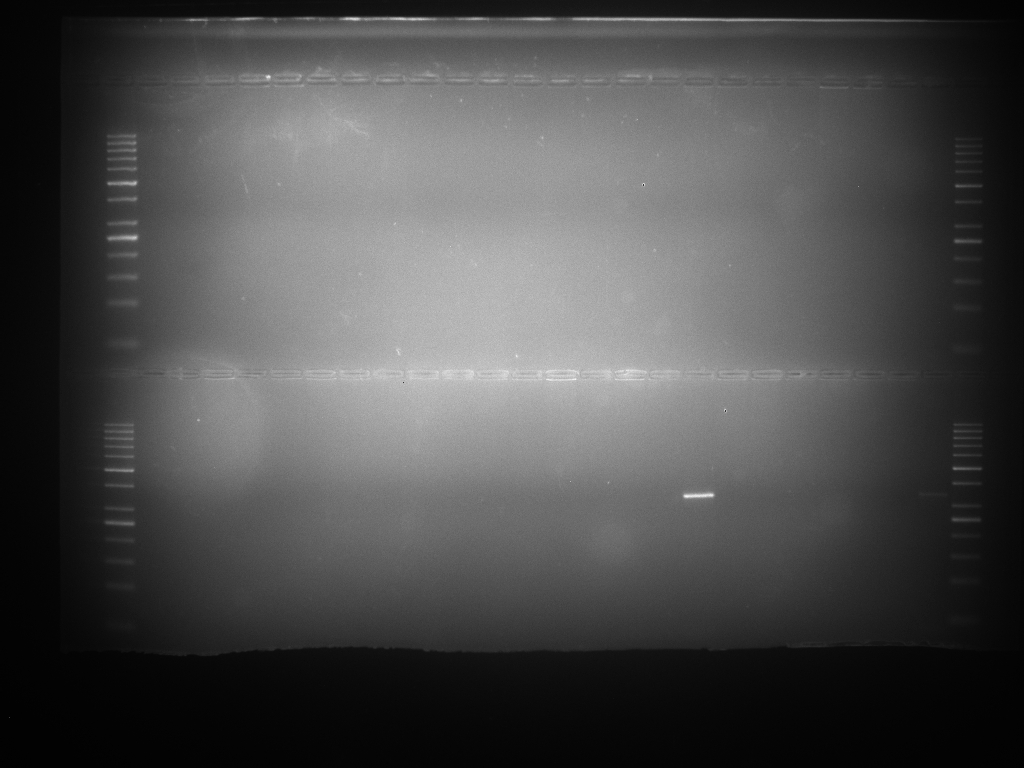

Supplement: S1 File — (ZIP) [file pone.0260246.s002.zip › BOESSR 122 11-3-2020 - Copy.jpg]

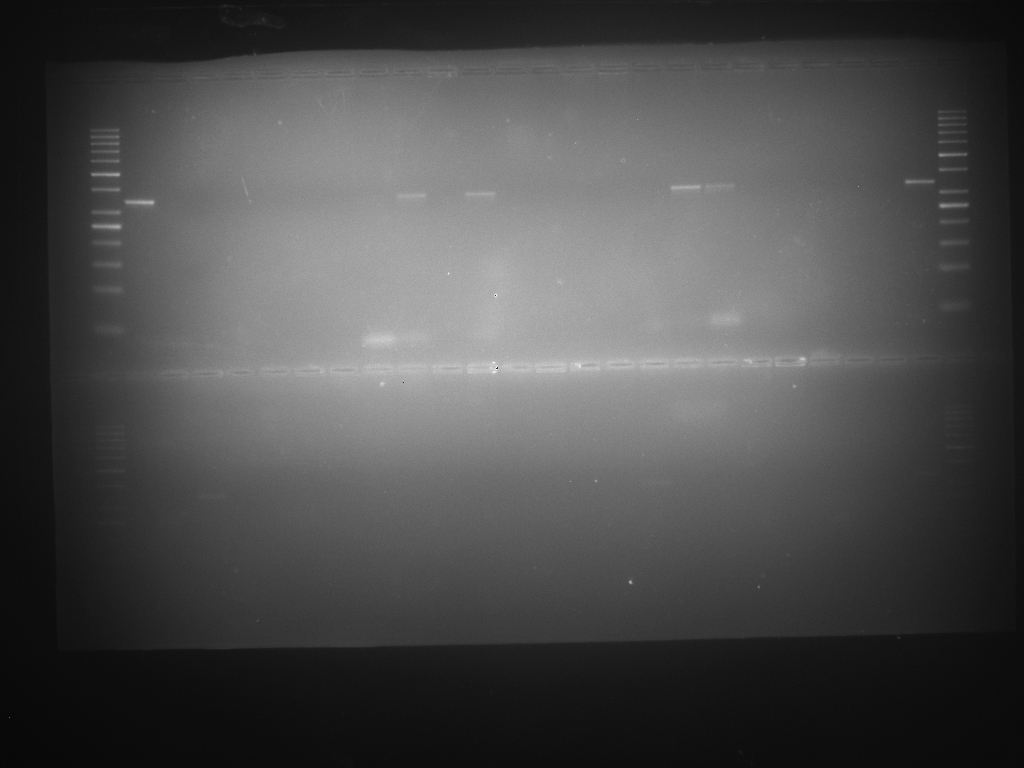

Supplement: S1 File — (ZIP) [file pone.0260246.s002.zip › BOESSR122 11-3-2020 LANE3,4 - Copy.jpg]

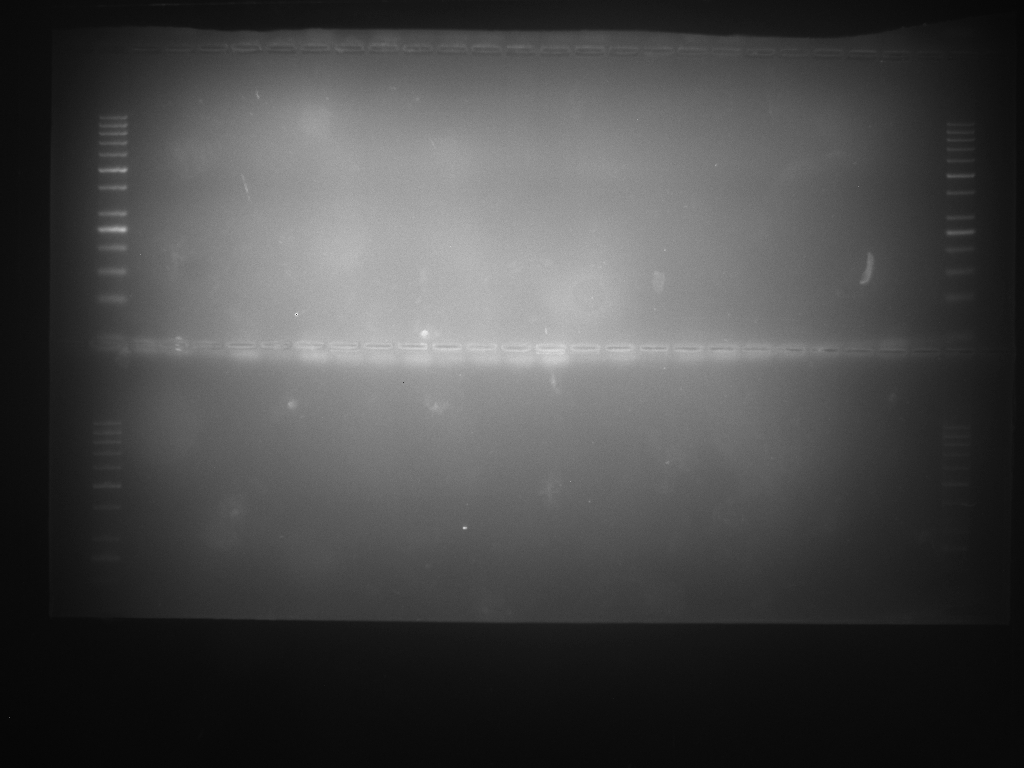

Supplement: S1 File — (ZIP) [file pone.0260246.s002.zip › boessr186 11-3-2020 lane 3,4.jpg]

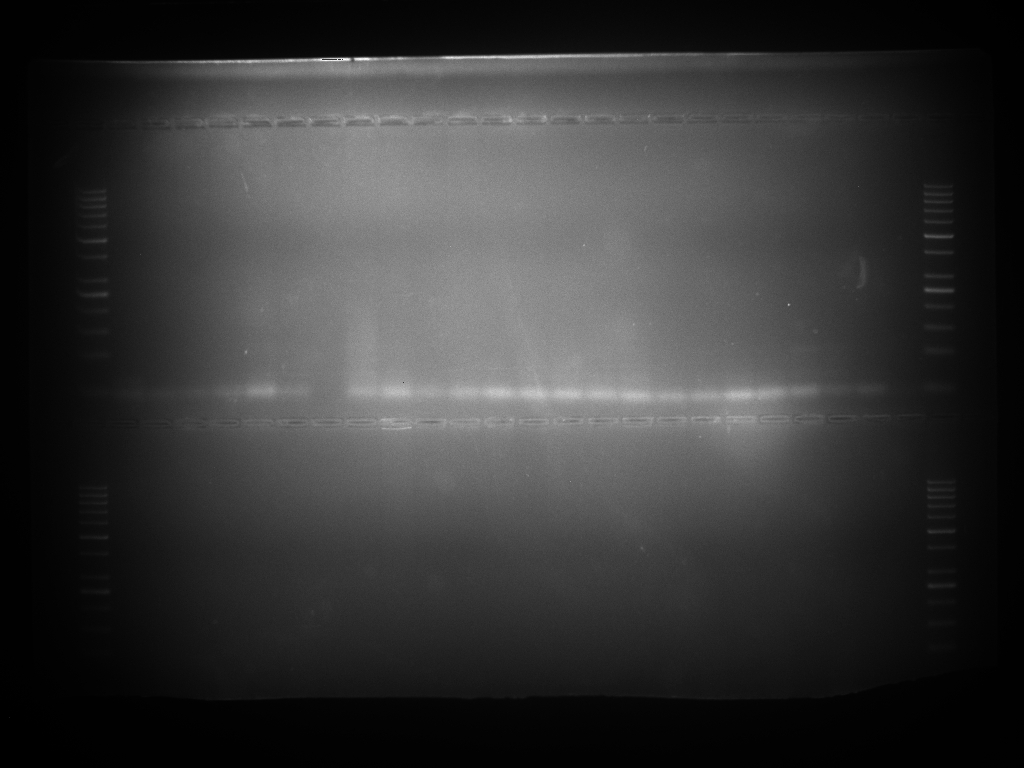

Supplement: S1 File — (ZIP) [file pone.0260246.s002.zip › boessr186 11-3-2020.jpg]

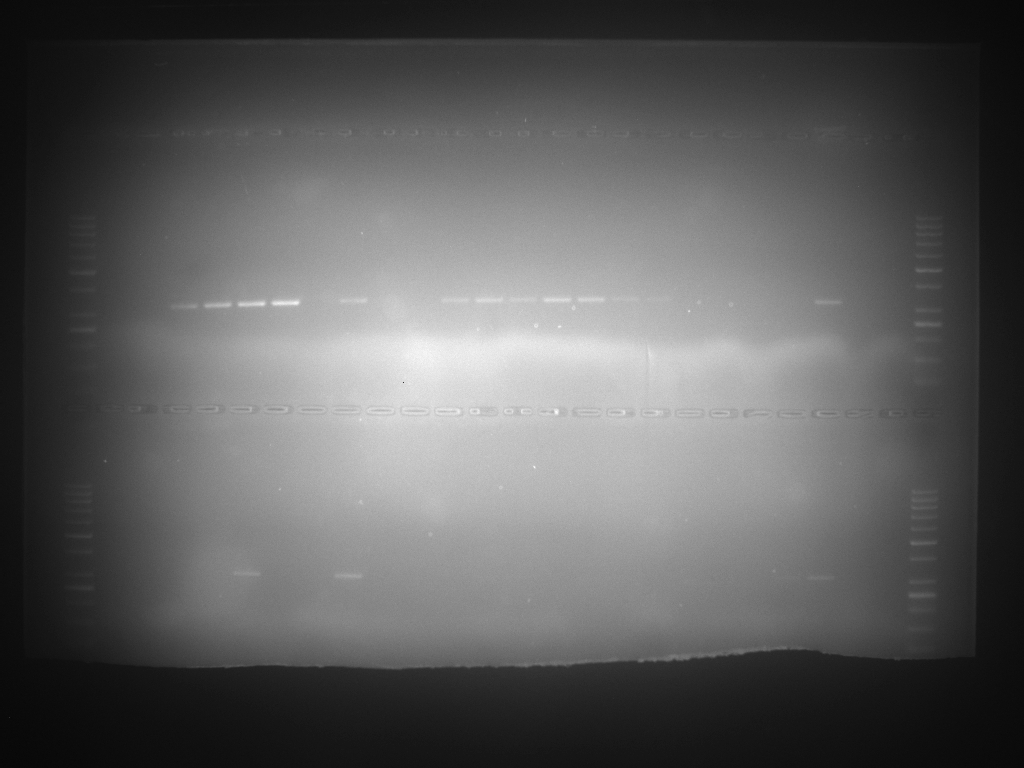

Supplement: S1 File — (ZIP) [file pone.0260246.s002.zip › BOESSR216 11-3-2020 LANE1 , 2.jpg]

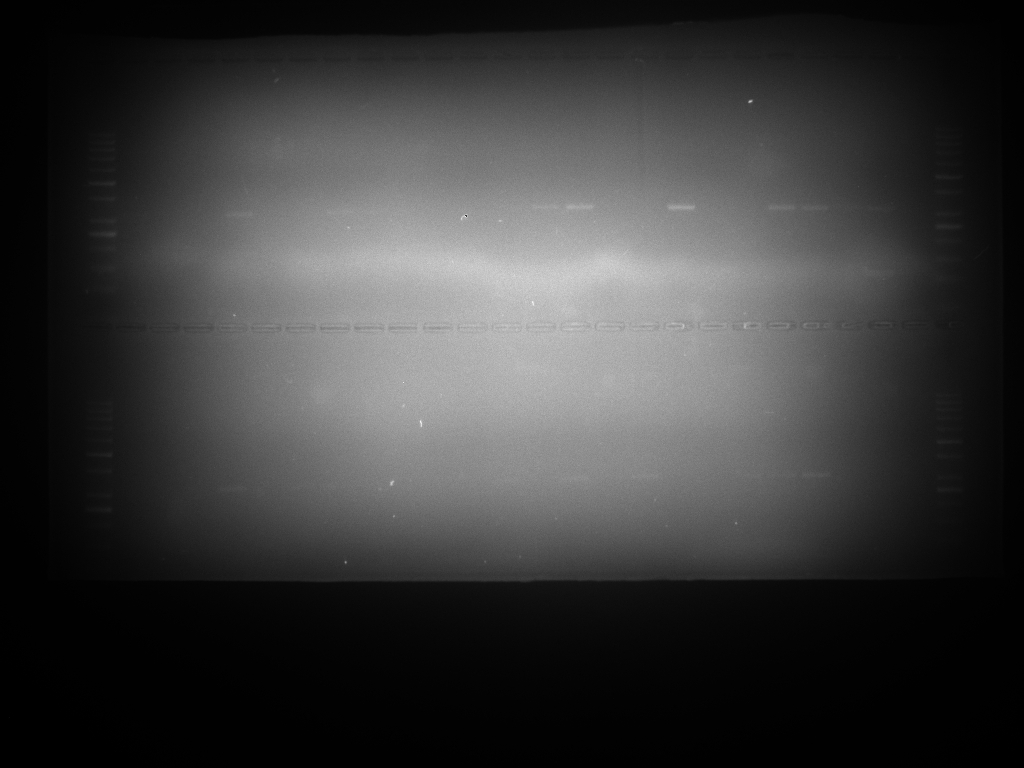

Supplement: S1 File — (ZIP) [file pone.0260246.s002.zip › BOESSR216 11-3-2020 LANE3 ,4.jpg]

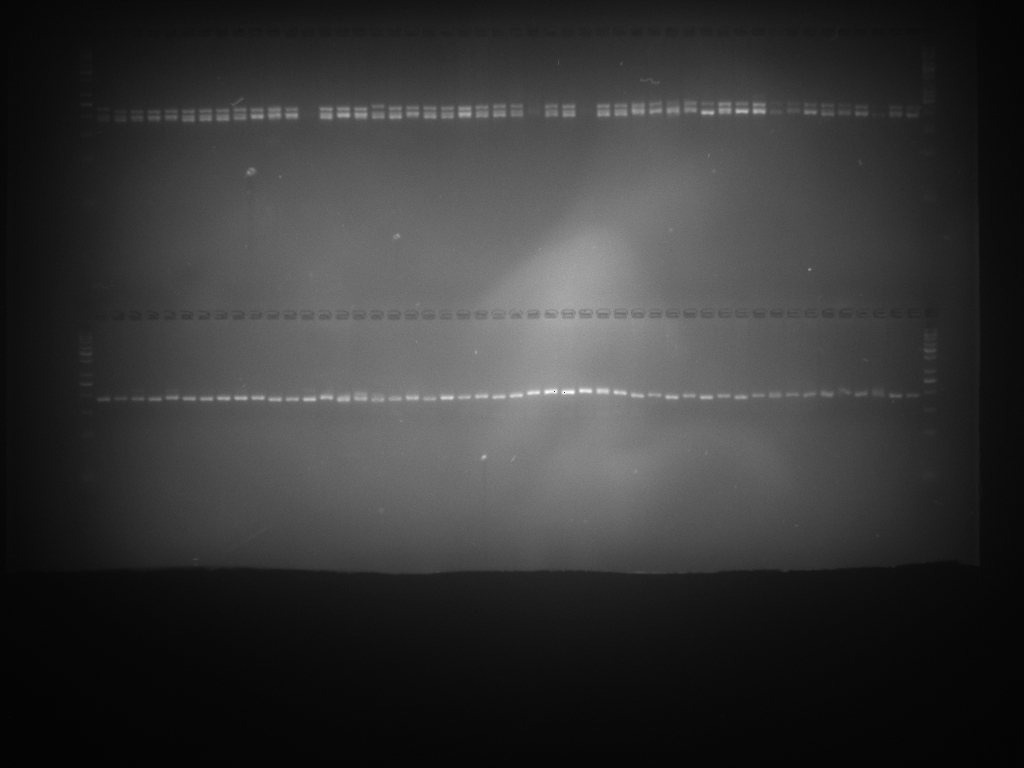

Supplement: S1 File — (ZIP) [file pone.0260246.s002.zip › BOESSR216 LANE 1 AND 2 3-2-2020.jpg]

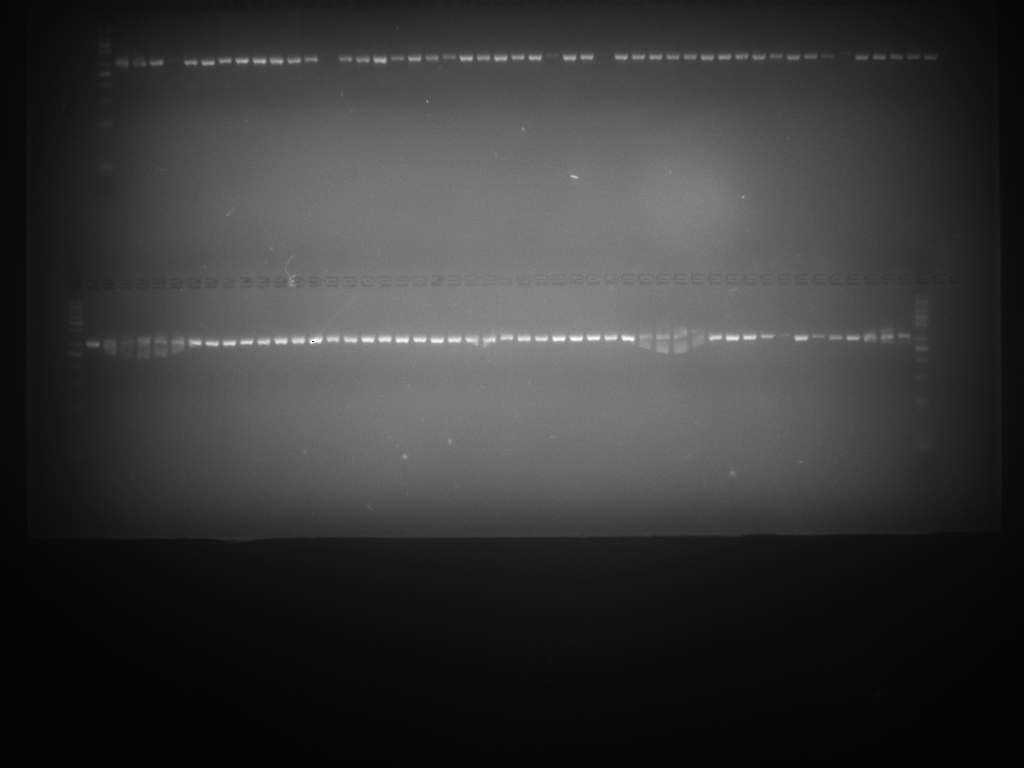

Supplement: S1 File — (ZIP) [file pone.0260246.s002.zip › BOESSSR719 LAE 3 AND 4 3-2-2020.jpg]

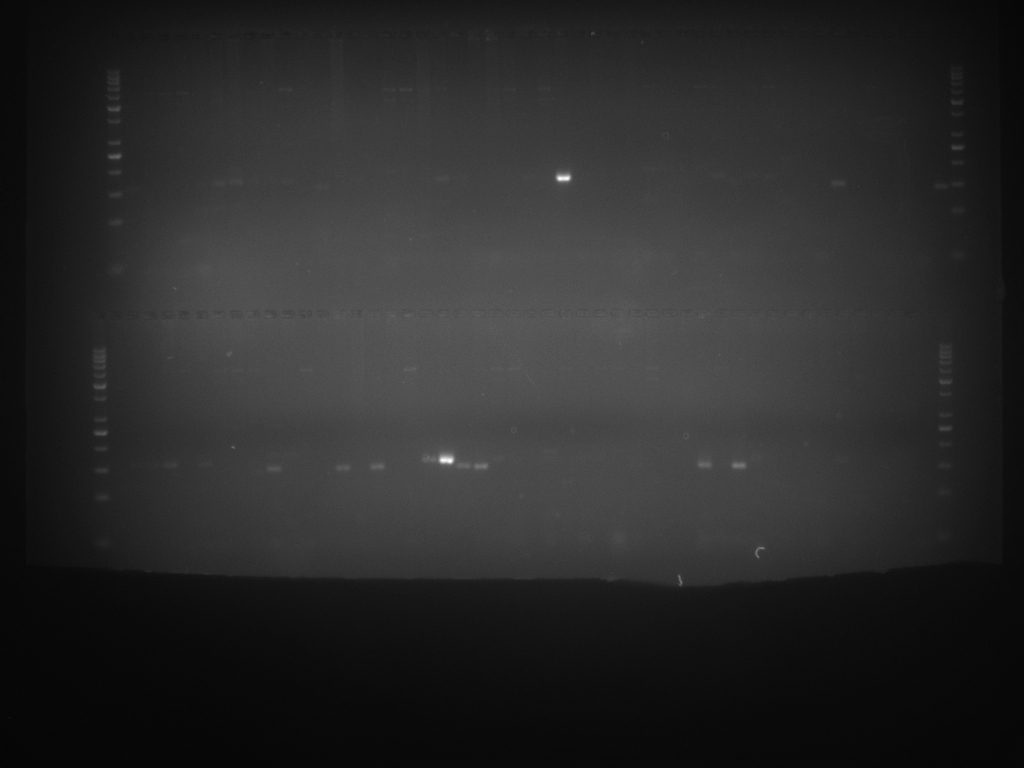

Supplement: S1 File — (ZIP) [file pone.0260246.s002.zip › BOGMS 0576 4-1-2020.jpg]

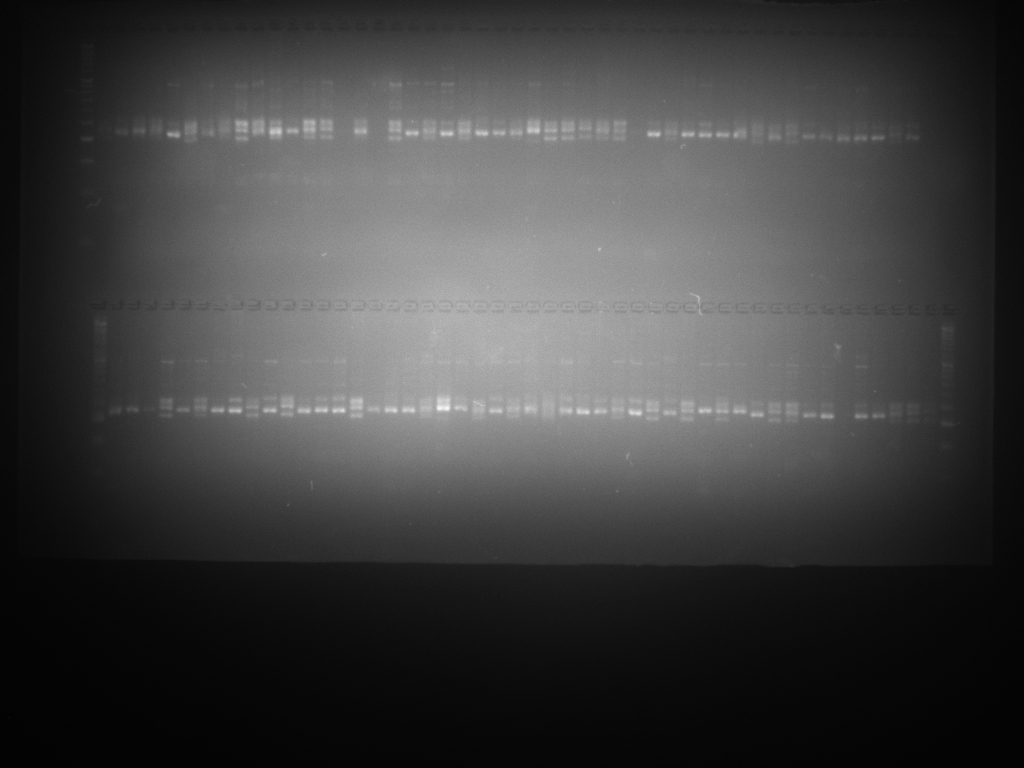

Supplement: S1 File — (ZIP) [file pone.0260246.s002.zip › BOGMS 0941 LANE 3 AND 4 3012020.jpg]

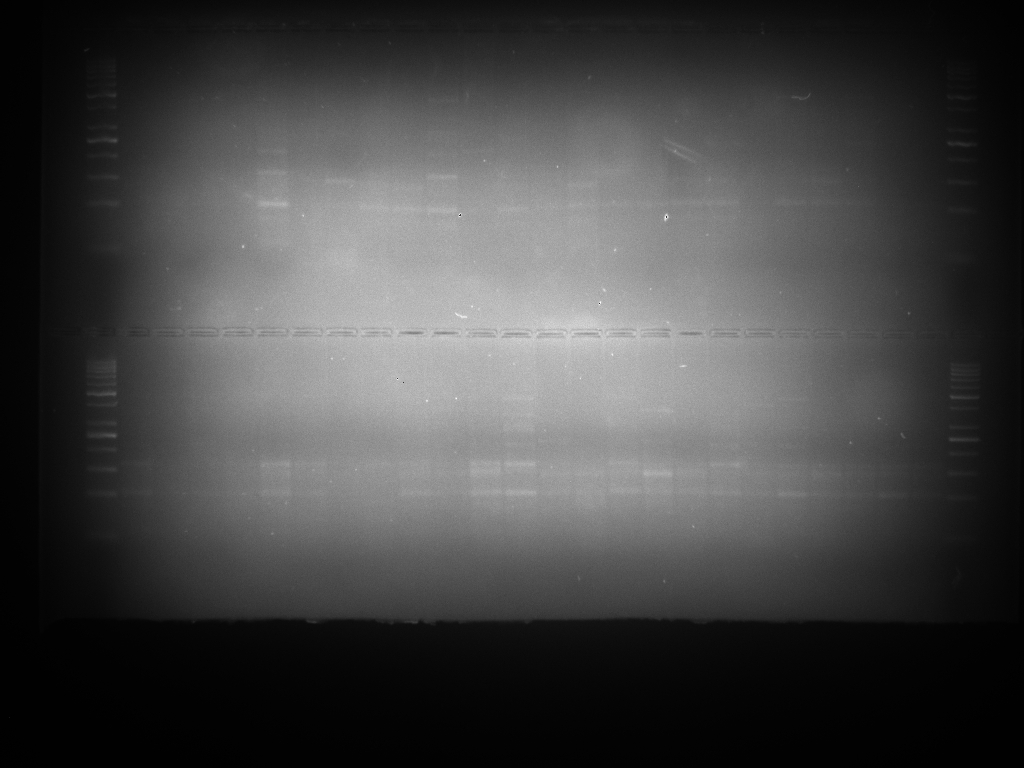

Supplement: S1 File — (ZIP) [file pone.0260246.s002.zip › bogms 1452 31-1-2020 lane 1,2.jpg]

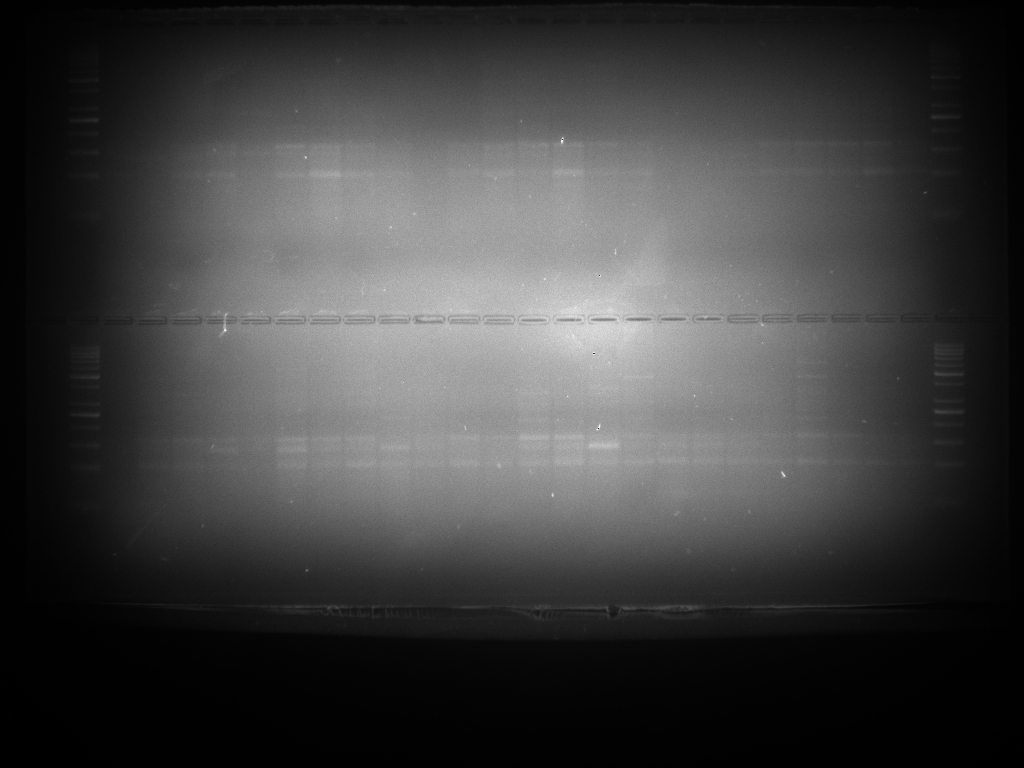

Supplement: S1 File — (ZIP) [file pone.0260246.s002.zip › bogms 1452 31-1-2020 lane 3 and 4.jpg]

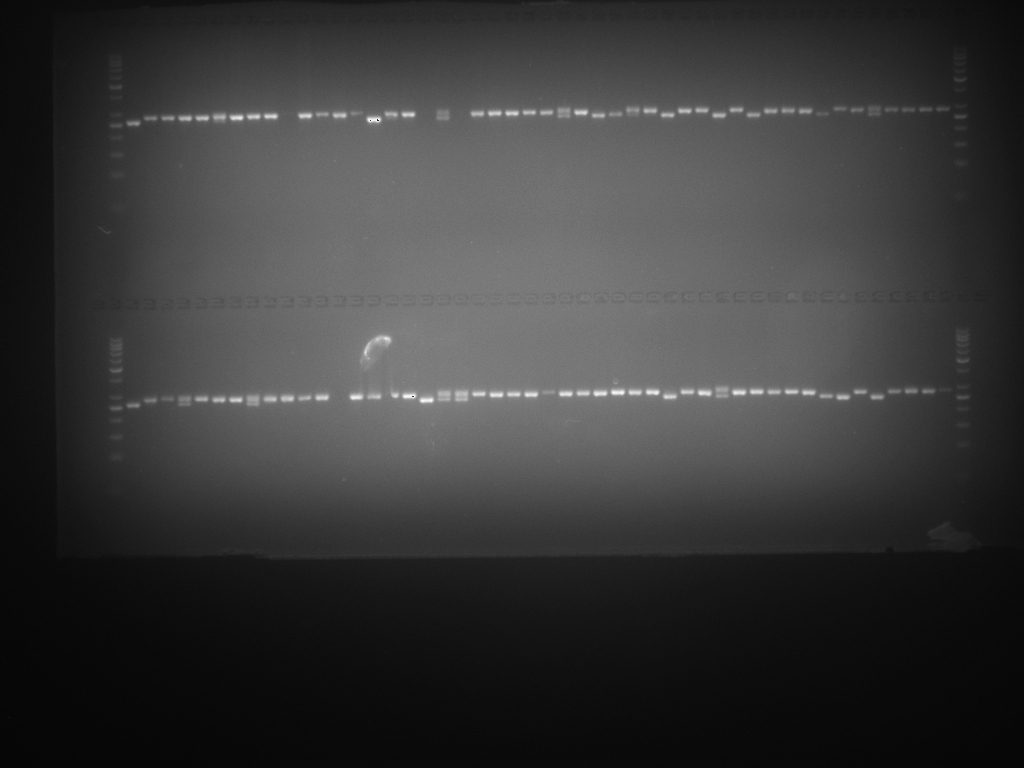

Supplement: S1 File — (ZIP) [file pone.0260246.s002.zip › bogms 1464 lane 3 and 4 1-2-2020.jpg]

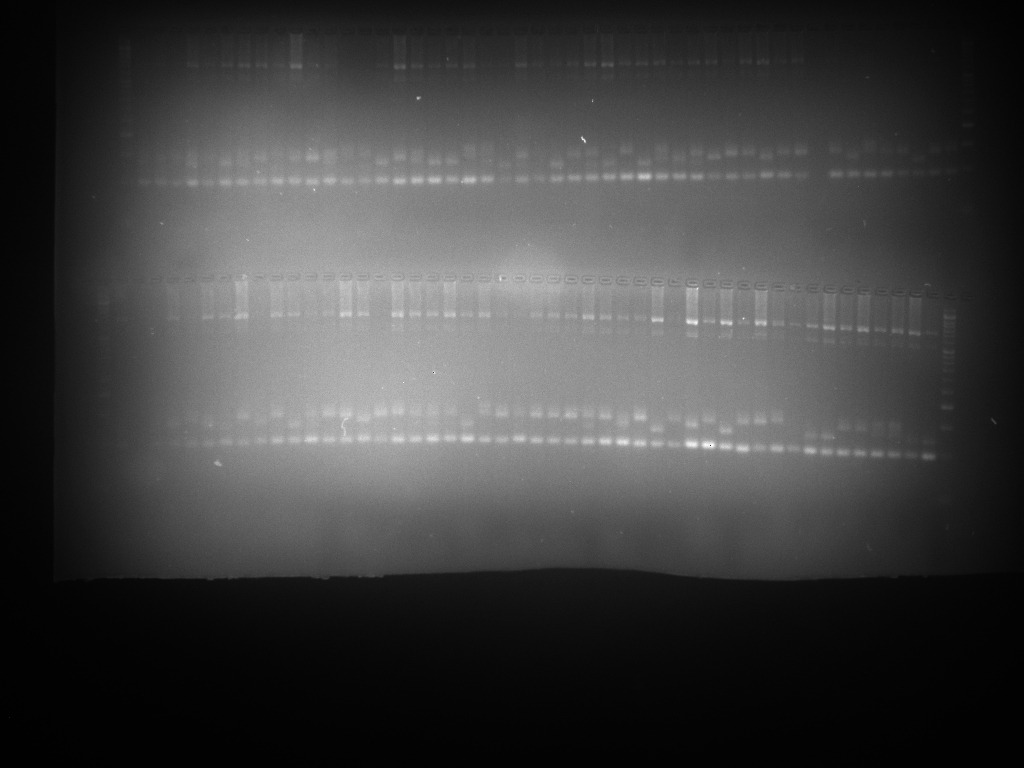

Supplement: S1 File — (ZIP) [file pone.0260246.s002.zip › bogms0162 lane 1 annd 2 28-12020.jpg]

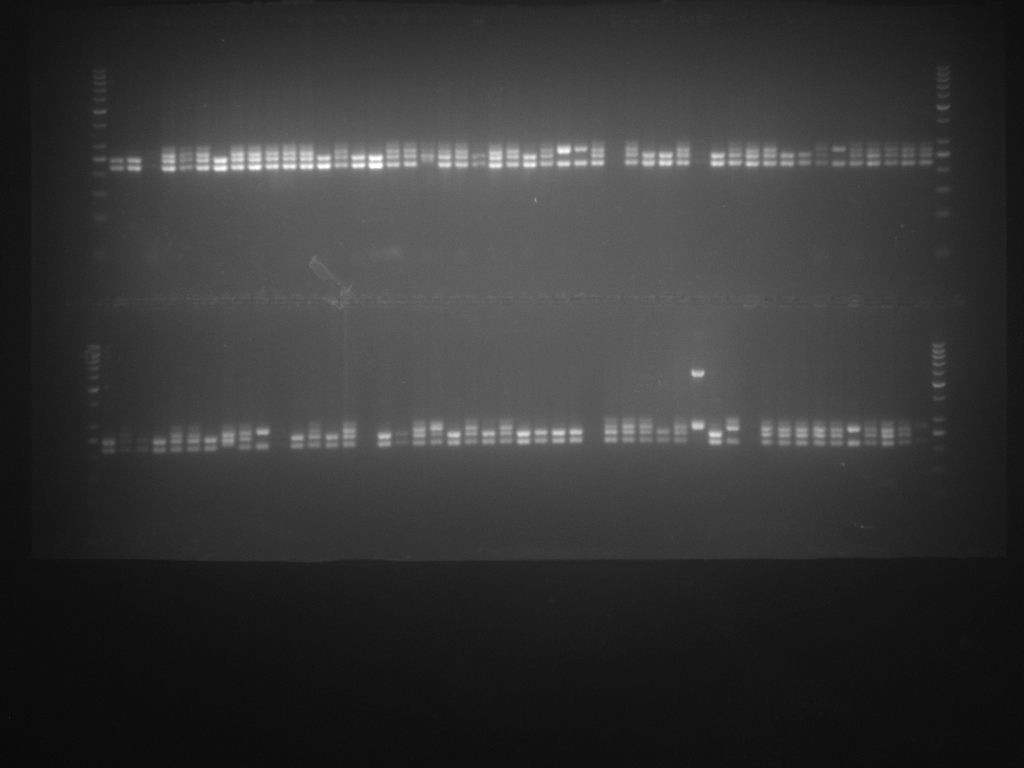

Supplement: S1 File — (ZIP) [file pone.0260246.s002.zip › BOGMS0327 30-1-2020 LANE 3 AND 4.jpg]

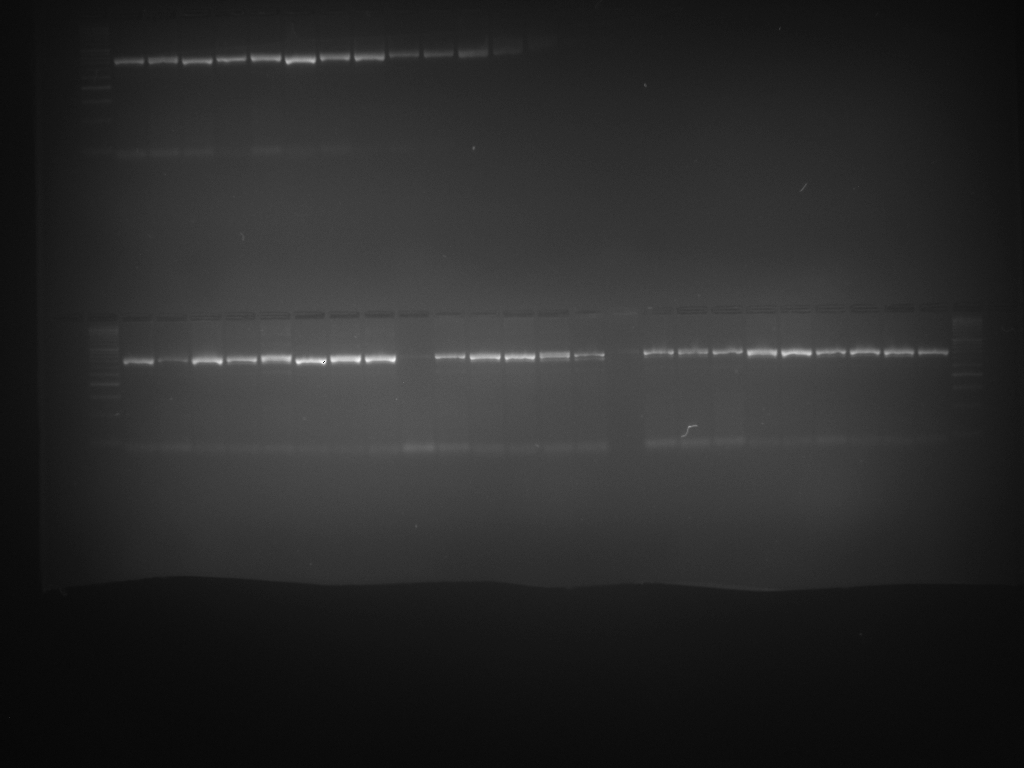

Supplement: S1 File — (ZIP) [file pone.0260246.s002.zip › BOGMS0374 LANE 1 AND 2 28-1-2020.jpg]

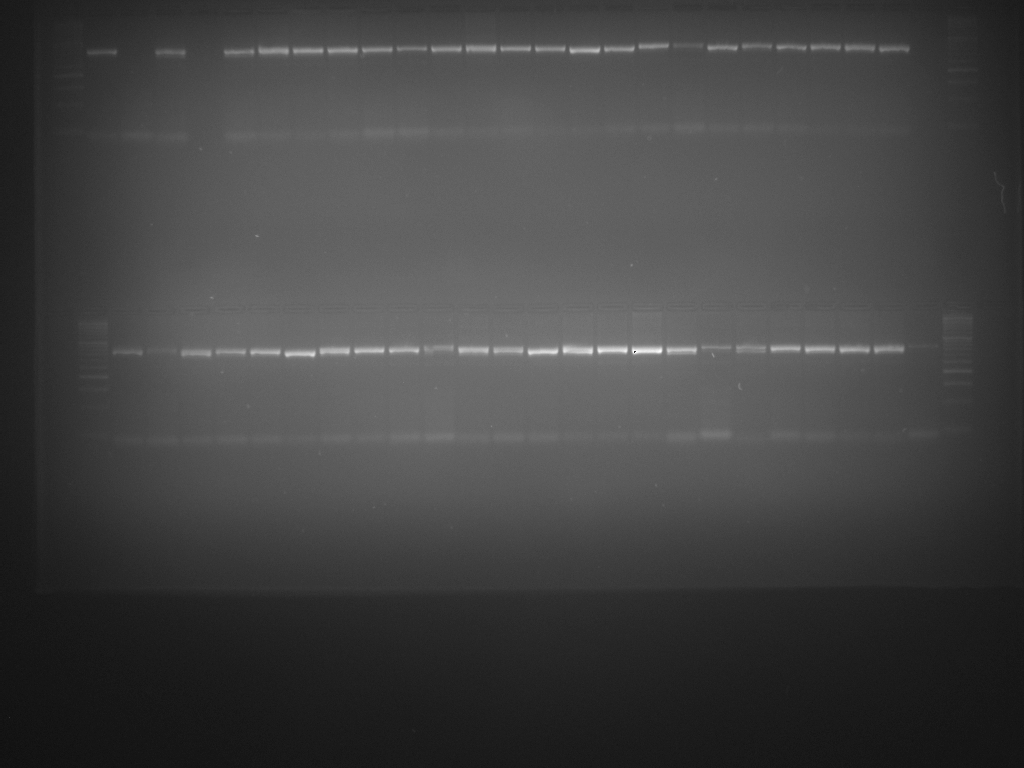

Supplement: S1 File — (ZIP) [file pone.0260246.s002.zip › BOGMS0374 LANE 3 AND 4 28-1-200.jpg]

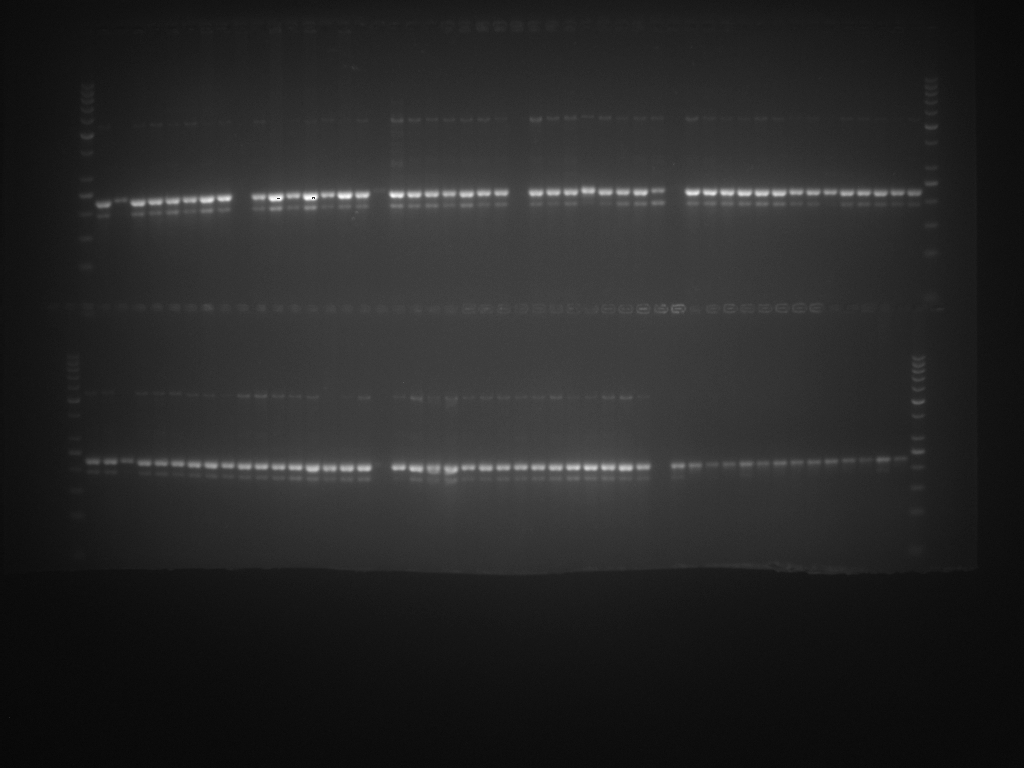

Supplement: S1 File — (ZIP) [file pone.0260246.s002.zip › BOGMS0596 30-1-2020 LANE 1 AND 2.jpg]

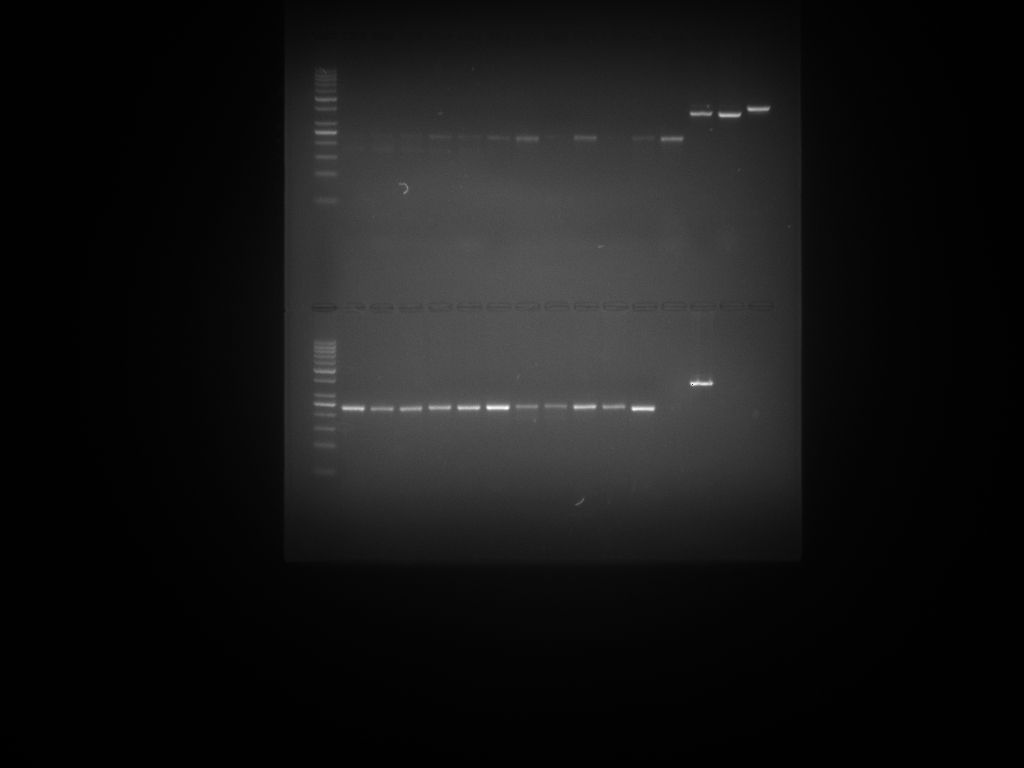

Supplement: S1 File — (ZIP) [file pone.0260246.s002.zip › BOGMS1432 (73-96) REPET 31-1-2020.jpg]

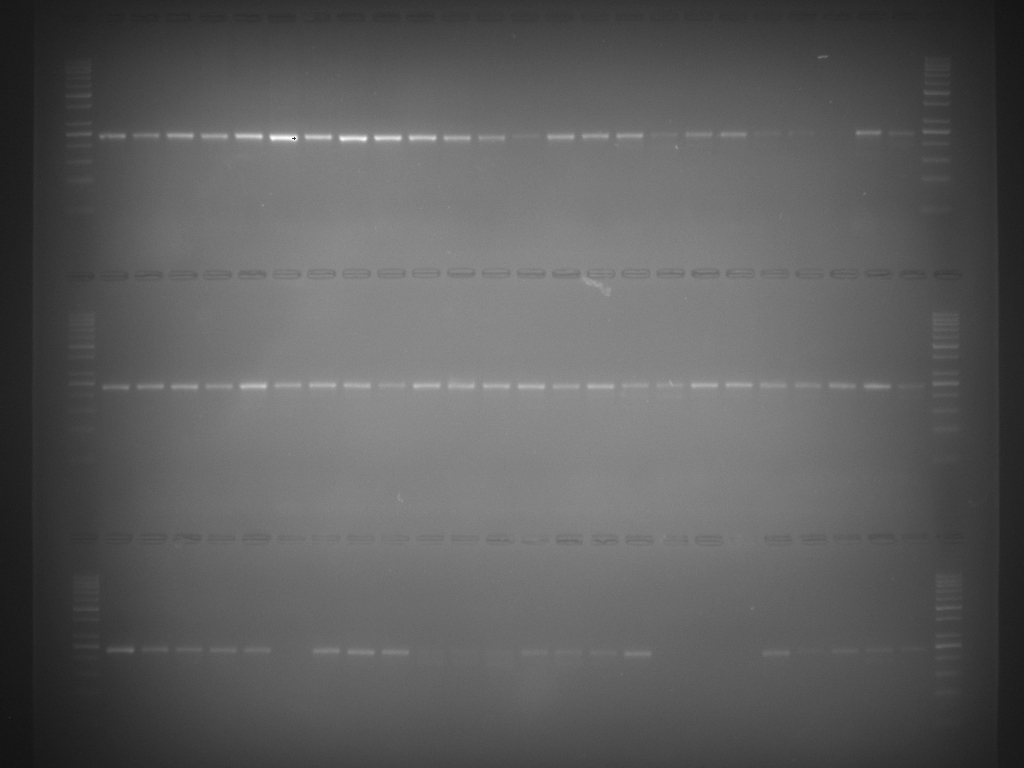

Supplement: S1 File — (ZIP) [file pone.0260246.s002.zip › BOGMS1432 31-1-2020 ( 1-72).jpg]

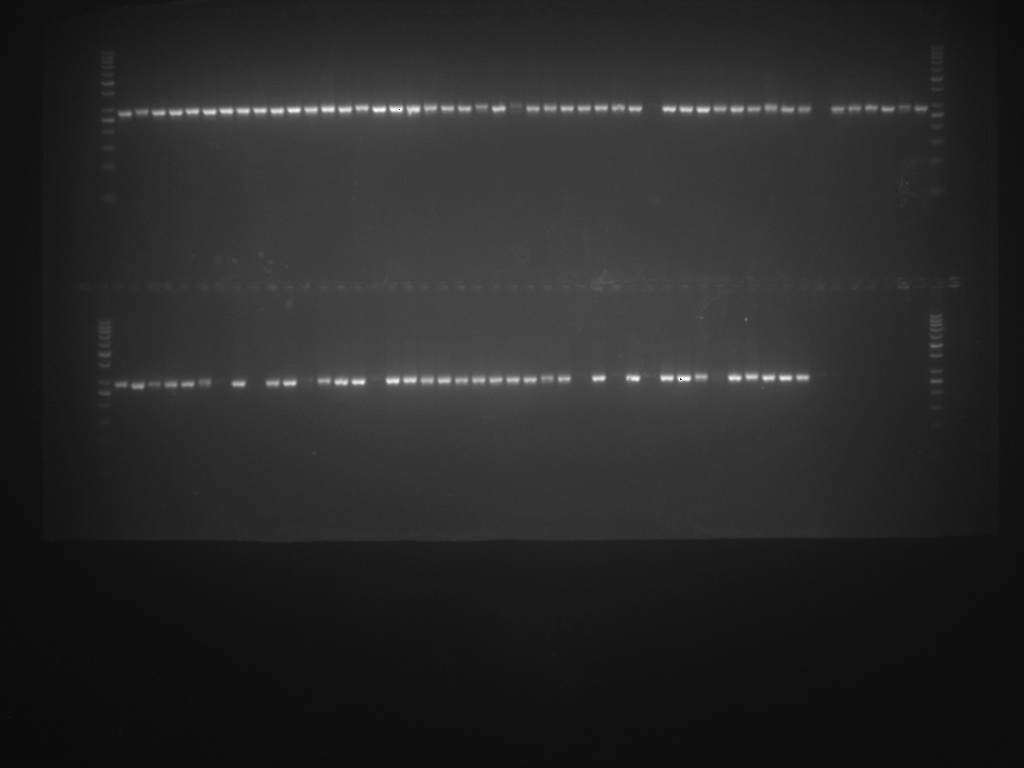

Supplement: S1 File — (ZIP) [file pone.0260246.s002.zip › BOSF 1162 22-2-2020 LANE 3 AND4.jpg]

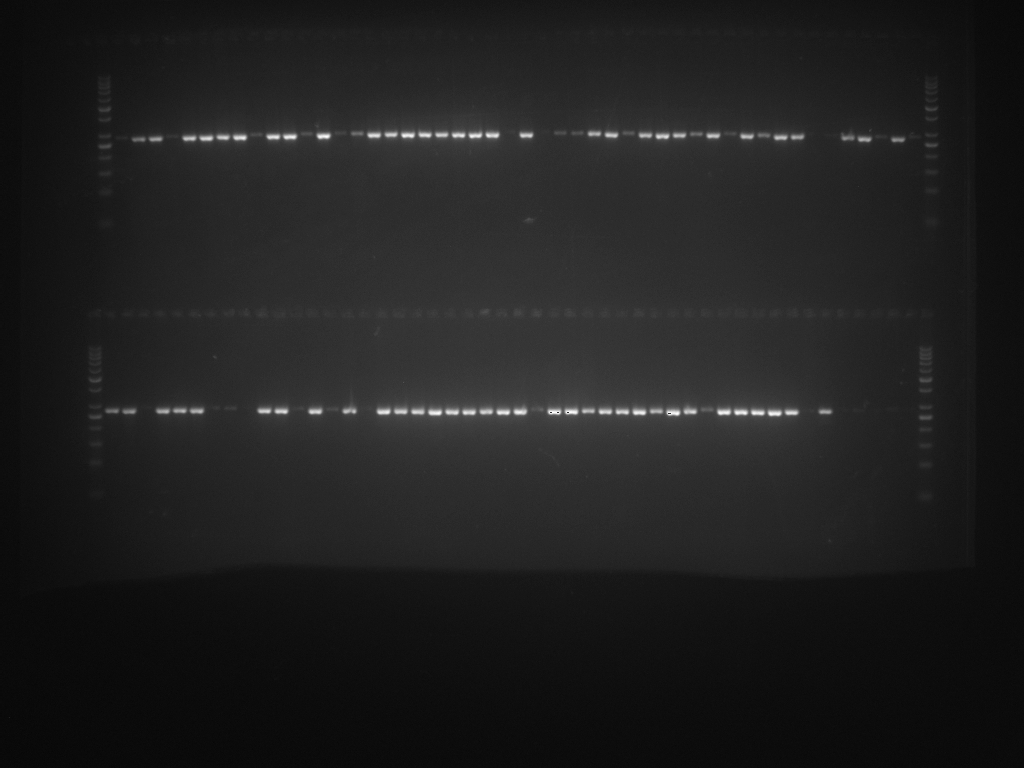

Supplement: S1 File — (ZIP) [file pone.0260246.s002.zip › BOSF 1166 LANE 1 AND 2 22-2-2020.jpg]

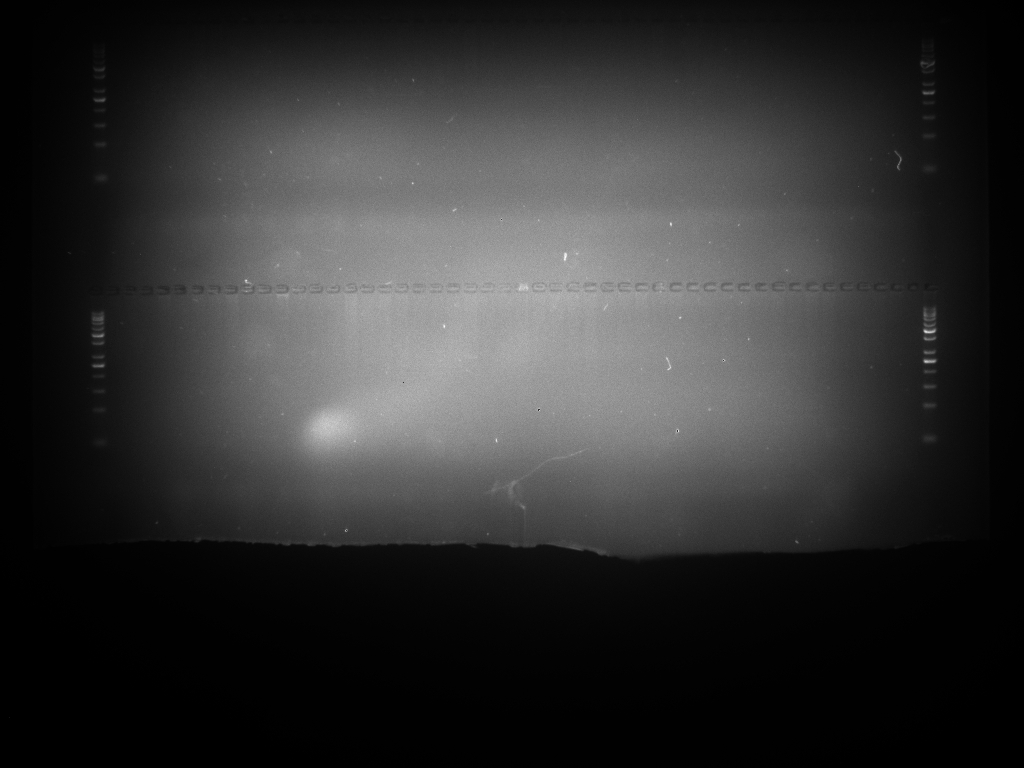

Supplement: S1 File — (ZIP) [file pone.0260246.s002.zip › BOSF 1279 REPET 21-2-2020.jpg]

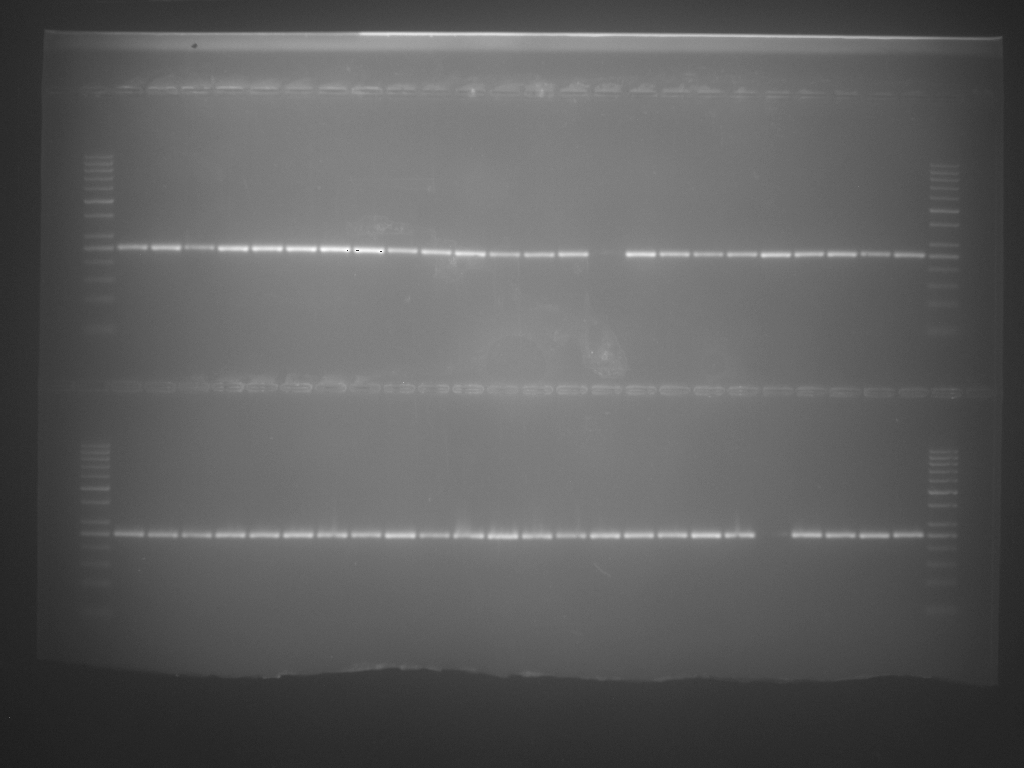

Supplement: S1 File — (ZIP) [file pone.0260246.s002.zip › BOSF1202 LANE 1,2 24-2-2020.jpg]

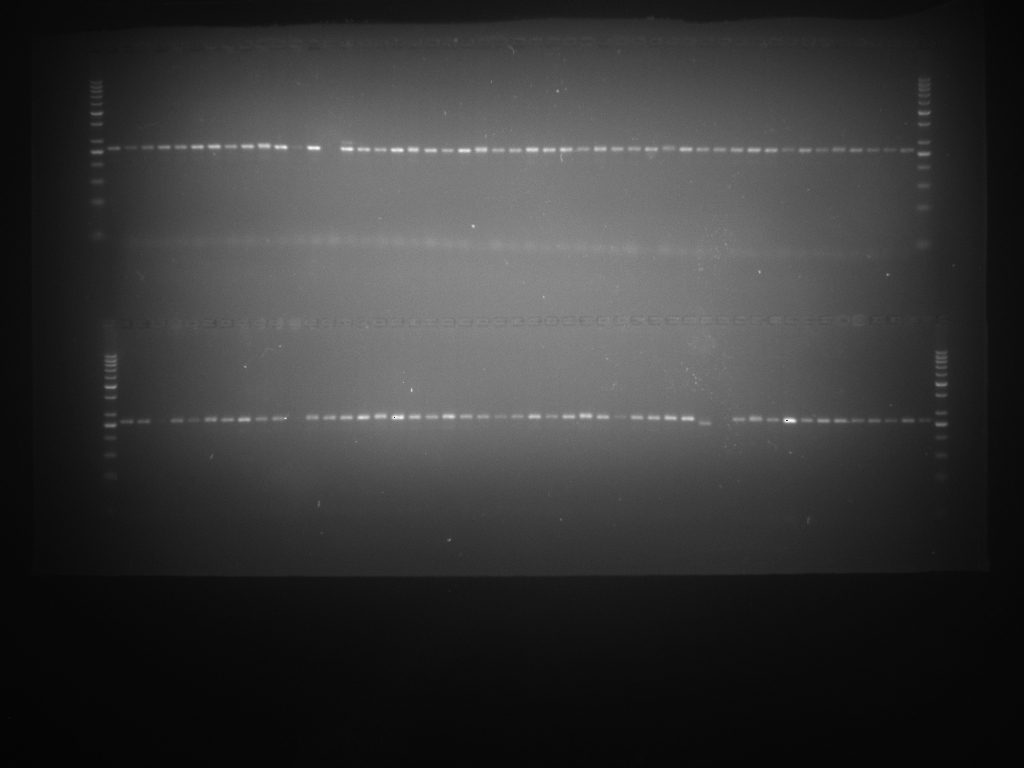

Supplement: S1 File — (ZIP) [file pone.0260246.s002.zip › BOSF1205 27-02-2020.jpg]

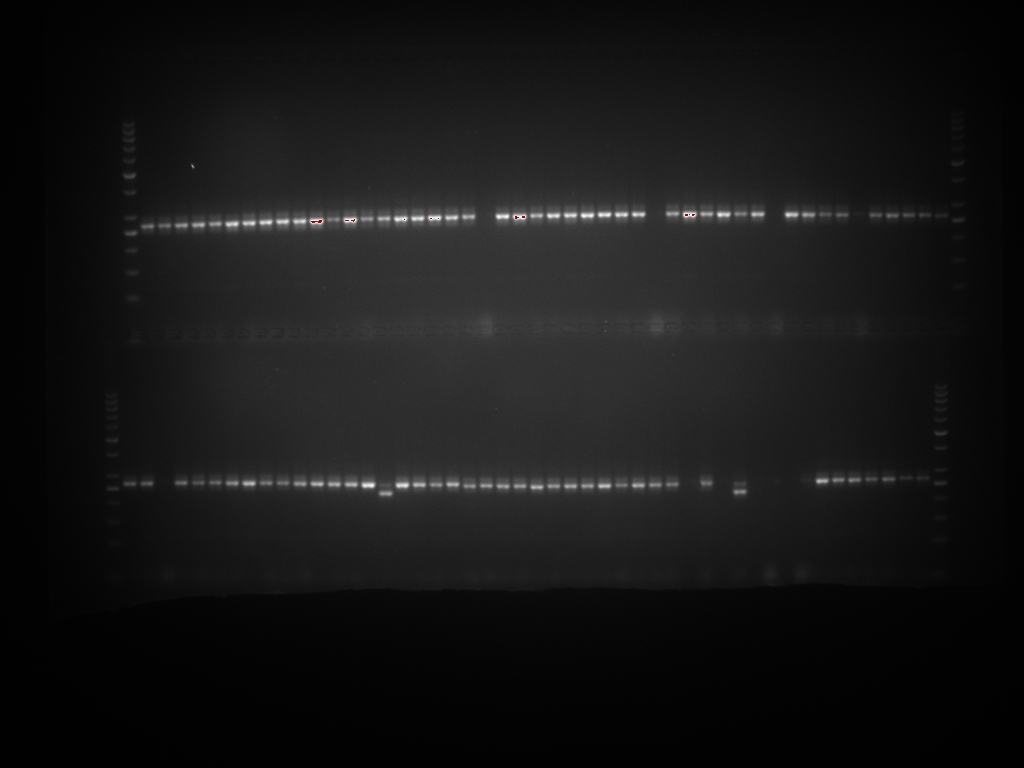

Supplement: S1 File — (ZIP) [file pone.0260246.s002.zip › BOSF1210 26-2-2020.jpg]

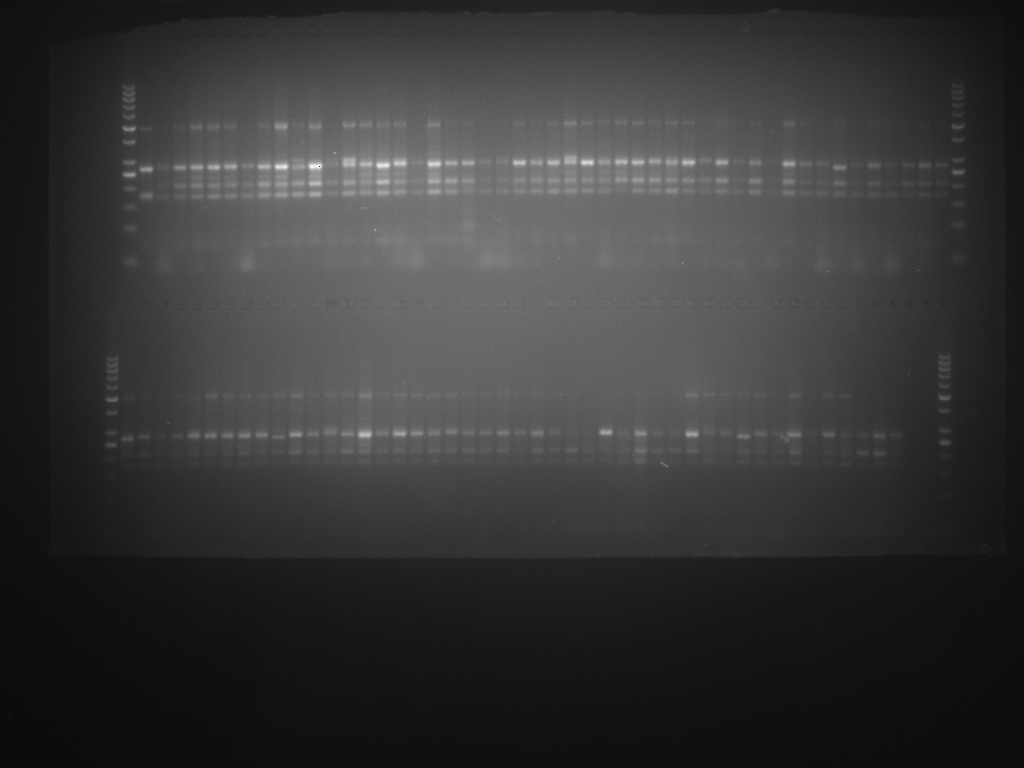

Supplement: S1 File — (ZIP) [file pone.0260246.s002.zip › BOSF1212 26-2-20.jpg]

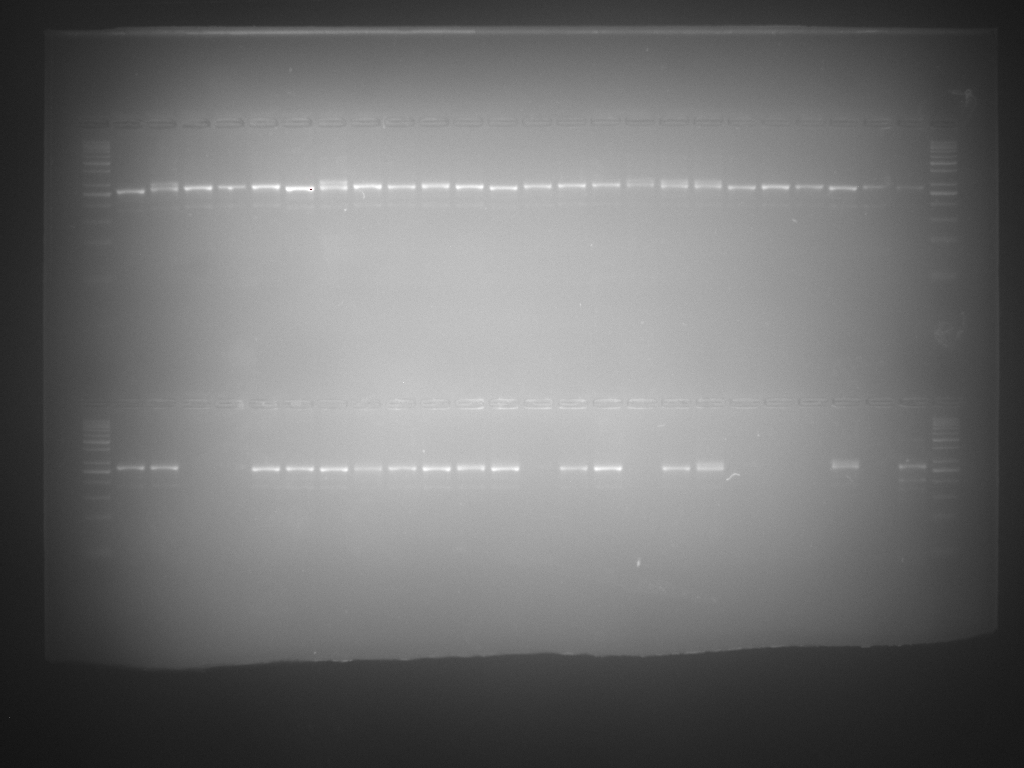

Supplement: S1 File — (ZIP) [file pone.0260246.s002.zip › BOSF1221 LANE 1,2 24-2-2029.jpg]

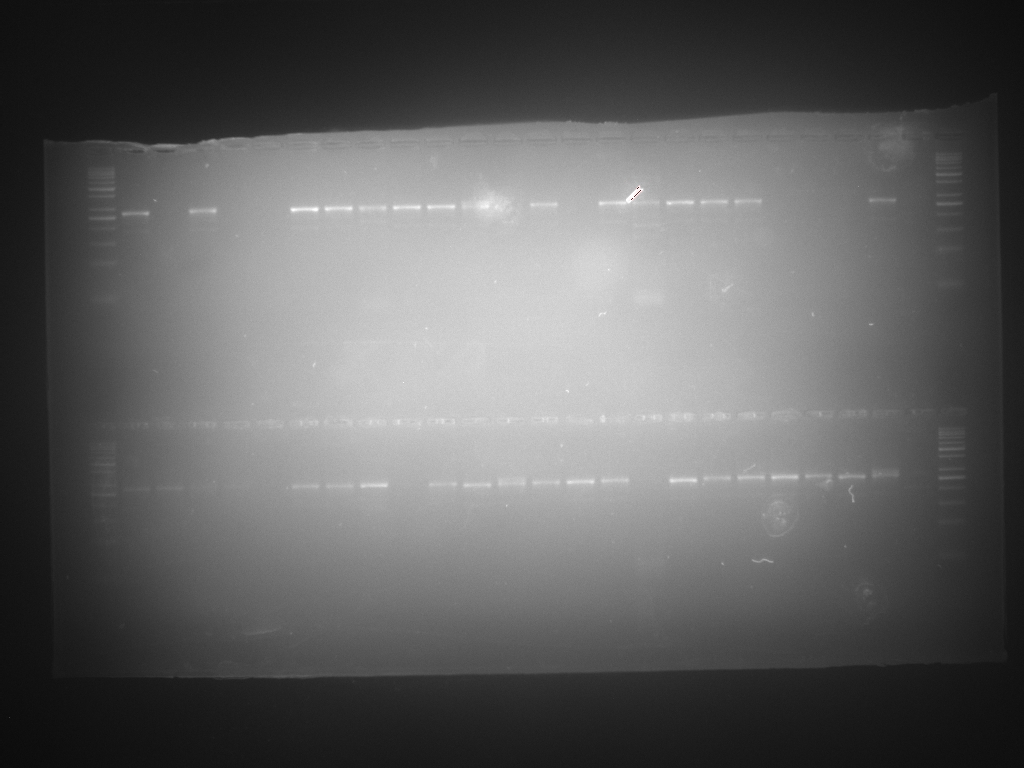

Supplement: S1 File — (ZIP) [file pone.0260246.s002.zip › BOSF1221 LANE 3,4 24-2-2020.jpg]

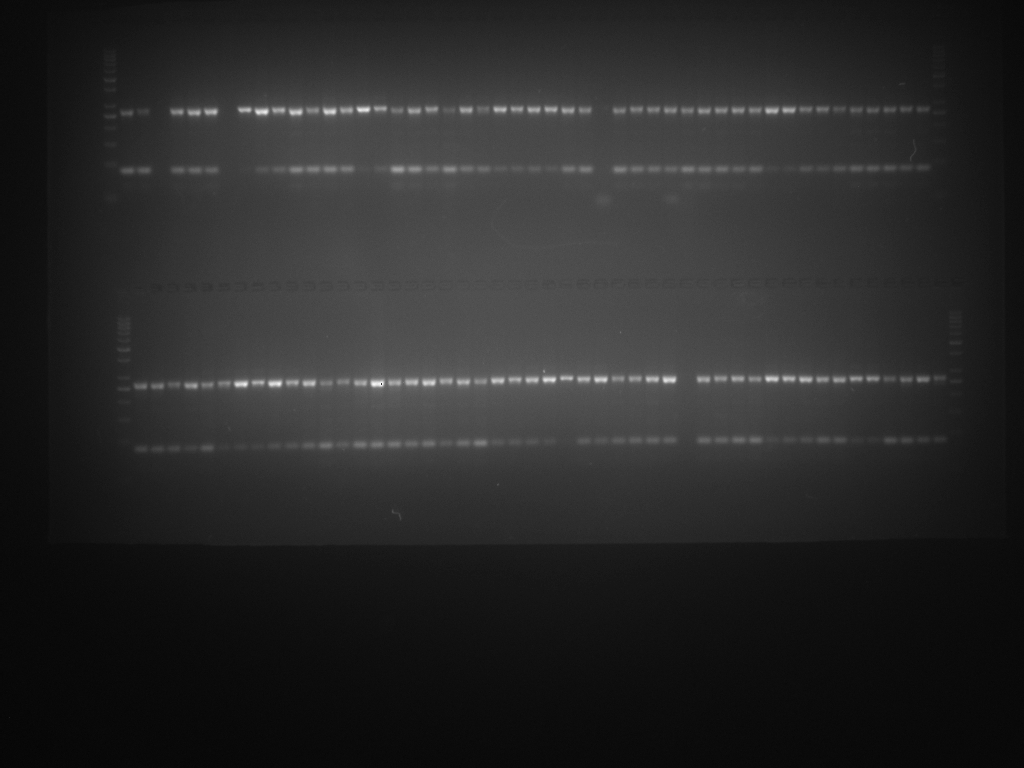

Supplement: S1 File — (ZIP) [file pone.0260246.s002.zip › BOSF1252 21-2-2020.jpg]

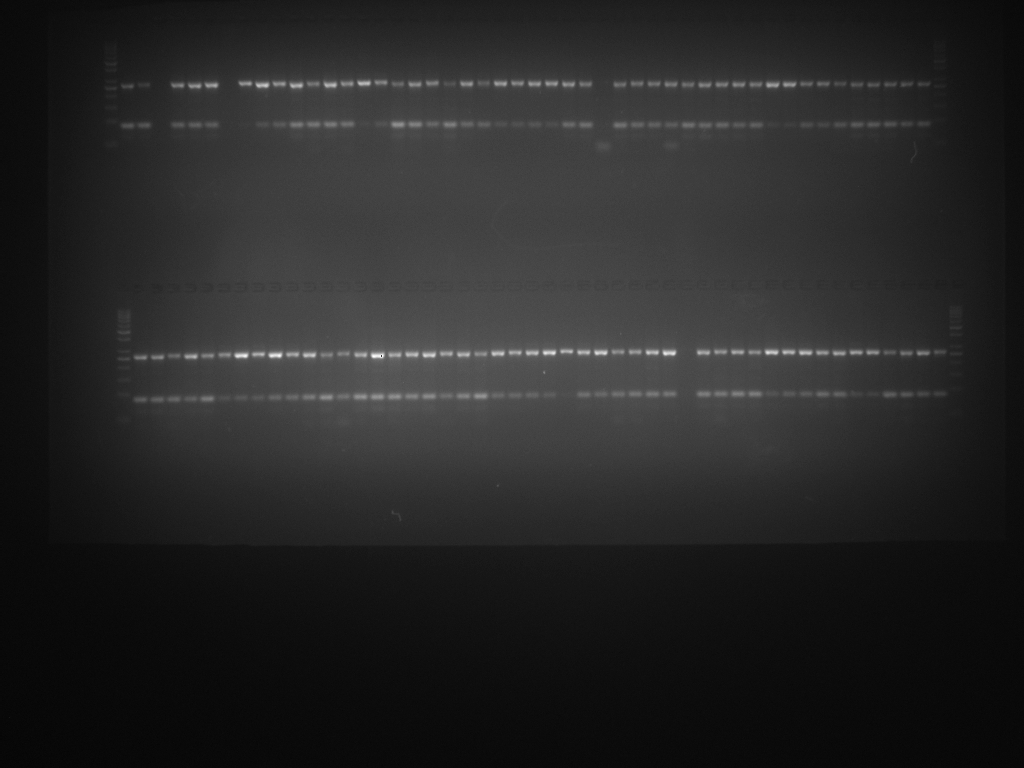

Supplement: S1 File — (ZIP) [file pone.0260246.s002.zip › BOSF1252 MORNING 21-2-2020.jpg]

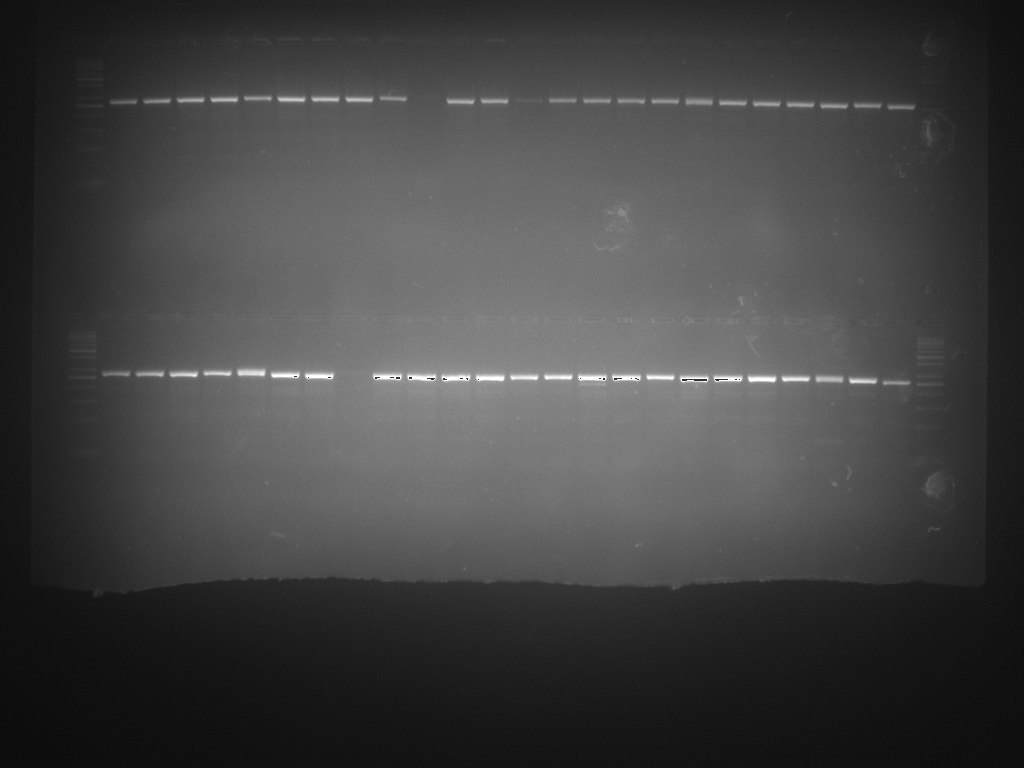

Supplement: S1 File — (ZIP) [file pone.0260246.s002.zip › BOSF1269 22-2-2020 LANE 1 AND 2.jpg]

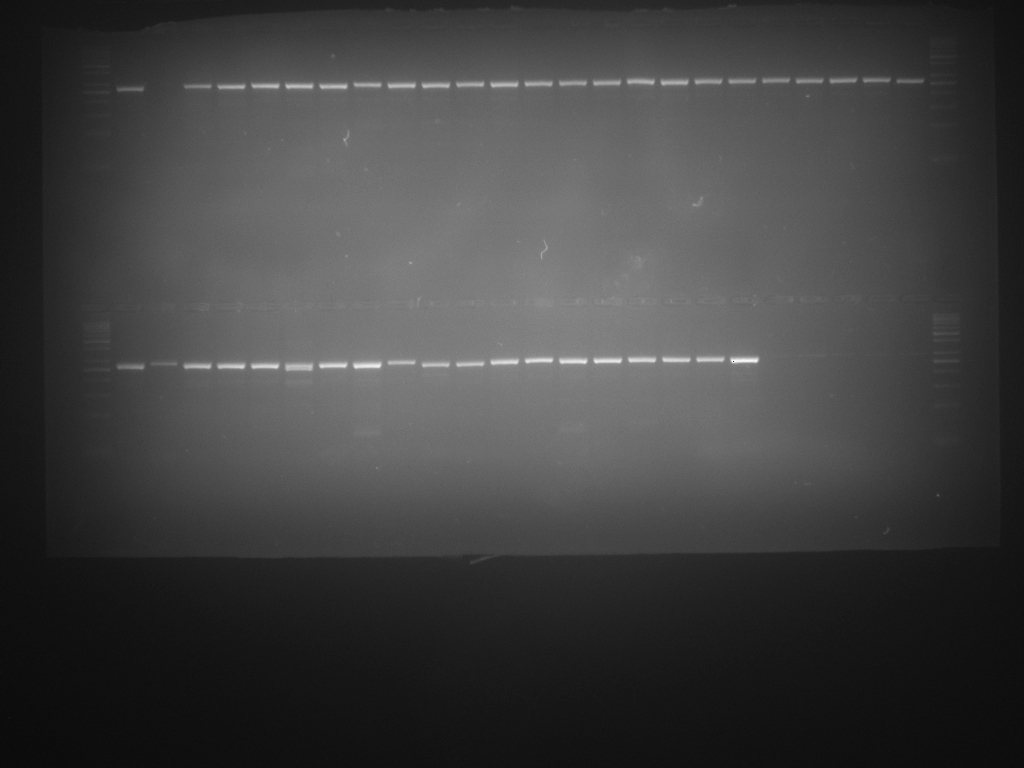

Supplement: S1 File — (ZIP) [file pone.0260246.s002.zip › BOSF1269 LANE 3 AND 4 22-2-2020.jpg]

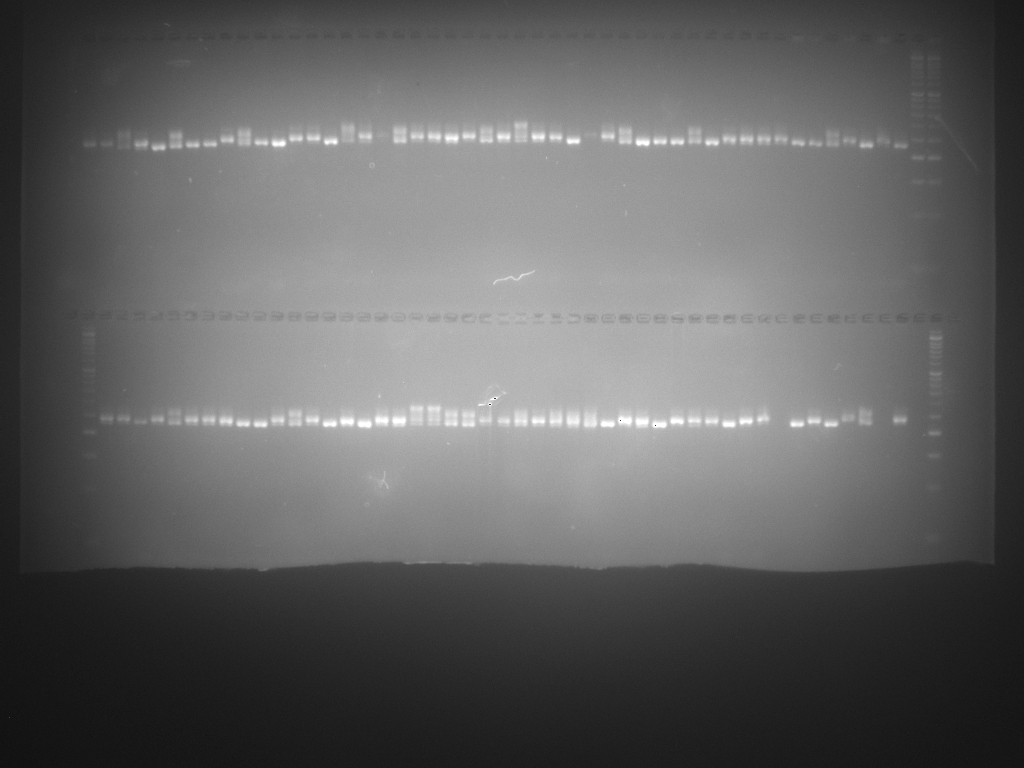

Supplement: S1 File — (ZIP) [file pone.0260246.s002.zip › BOSF2717 3012020 LAE 1 AND 2.jpg]

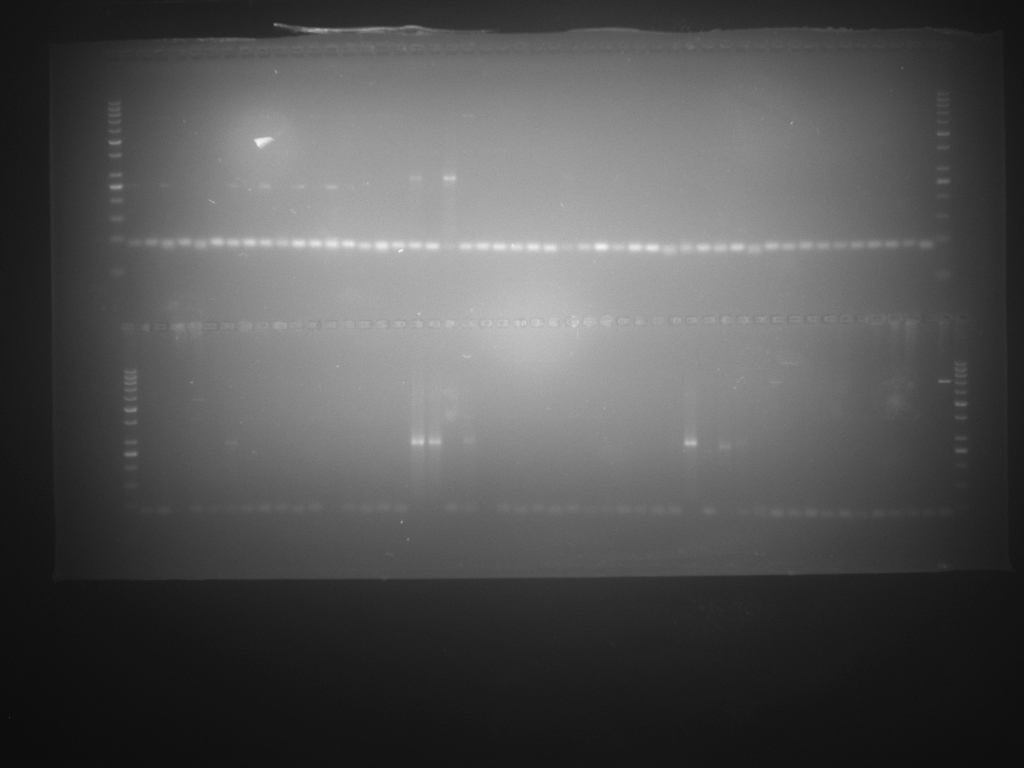

Supplement: S1 File — (ZIP) [file pone.0260246.s002.zip › CB1034A 28-2-2020.jpg]

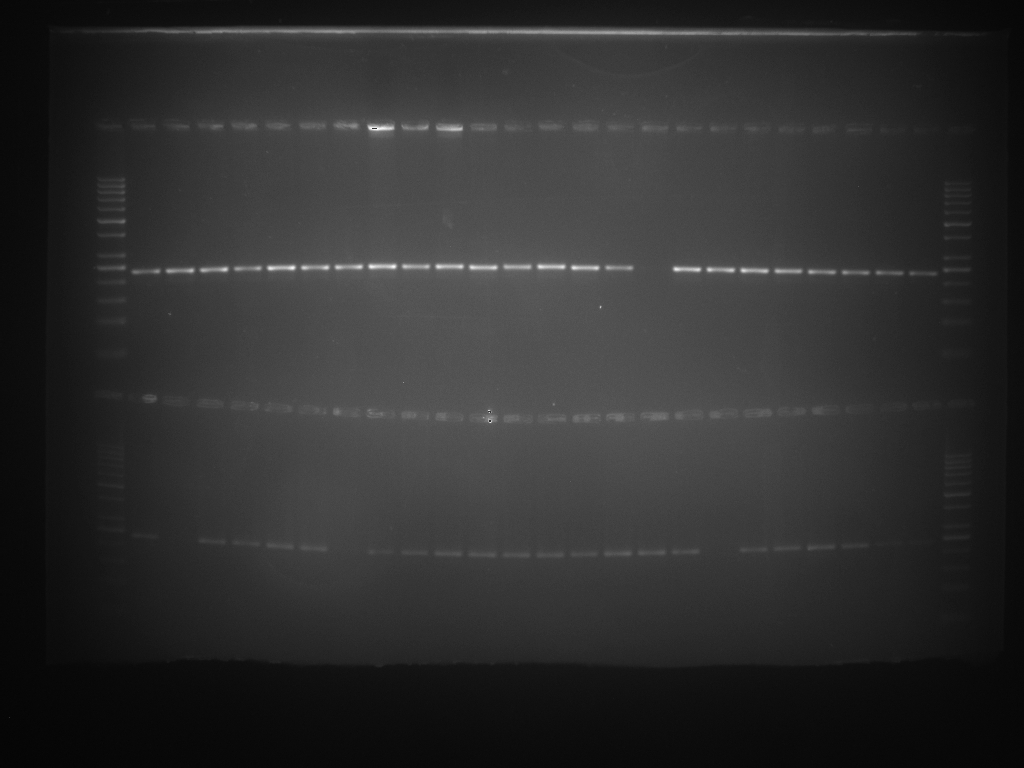

Supplement: S1 File — (ZIP) [file pone.0260246.s002.zip › CB10089 28-2-2020.jpg]

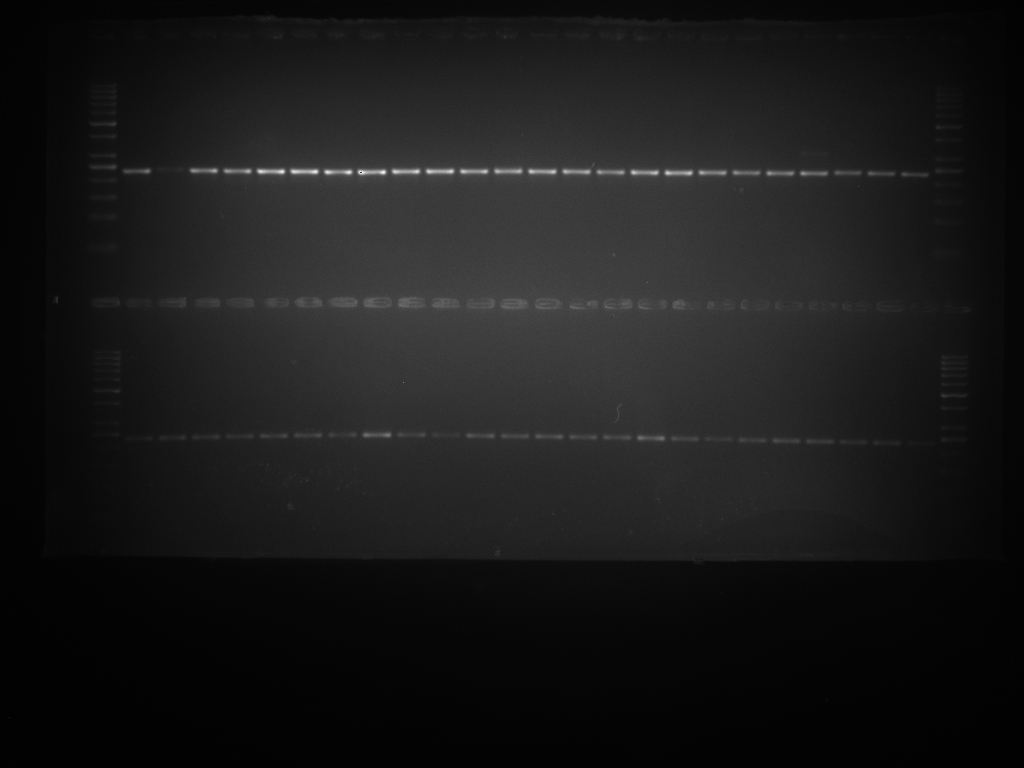

Supplement: S1 File — (ZIP) [file pone.0260246.s002.zip › CB10089 LANE3,4 28-2-2020.jpg]

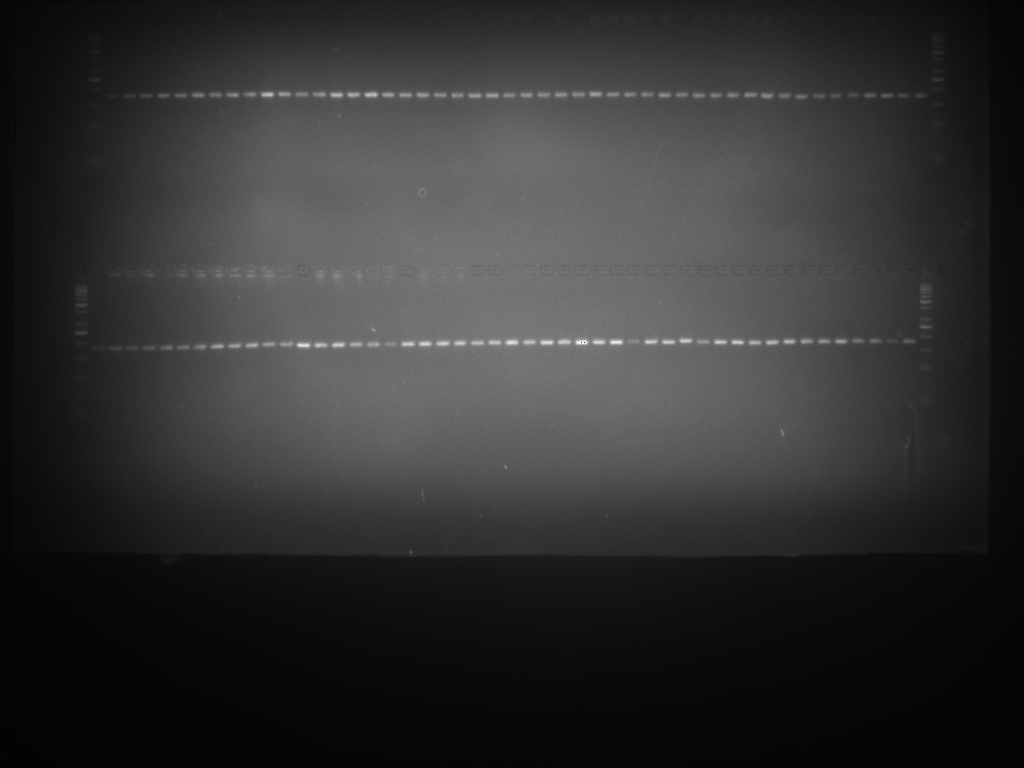

Supplement: S1 File — (ZIP) [file pone.0260246.s002.zip › cb10179.jpg]

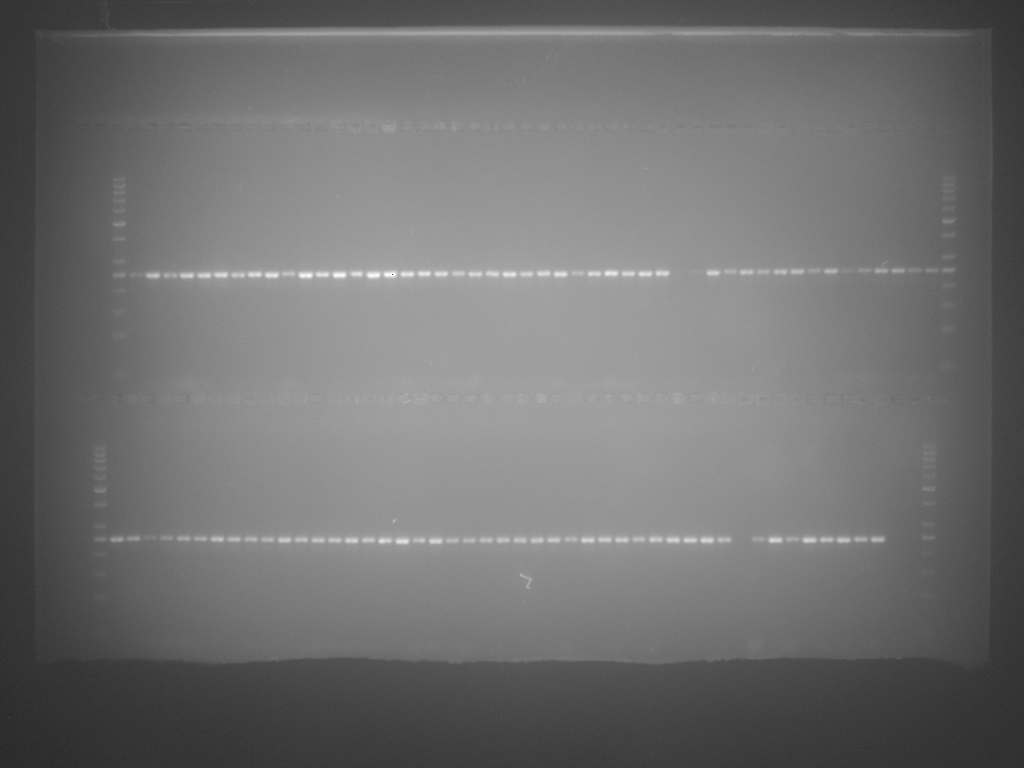

Supplement: S1 File — (ZIP) [file pone.0260246.s002.zip › CB10350 28-2-2020.jpg]

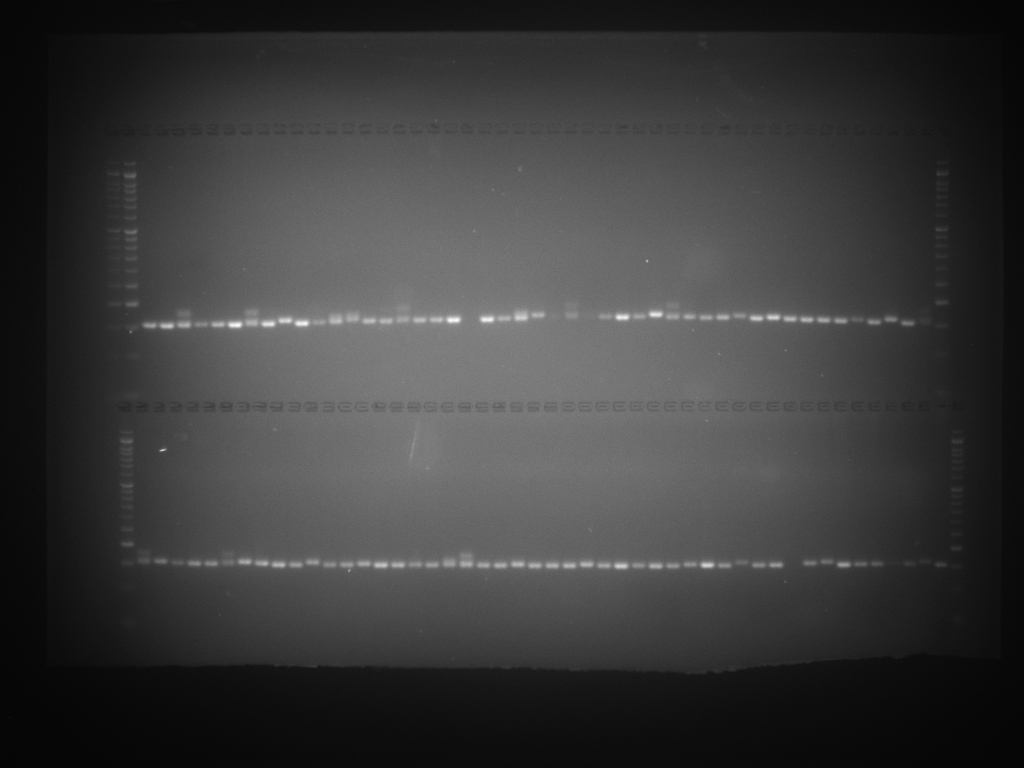

Supplement: S1 File — (ZIP) [file pone.0260246.s002.zip › CB10623 1-3-2020.jpg]

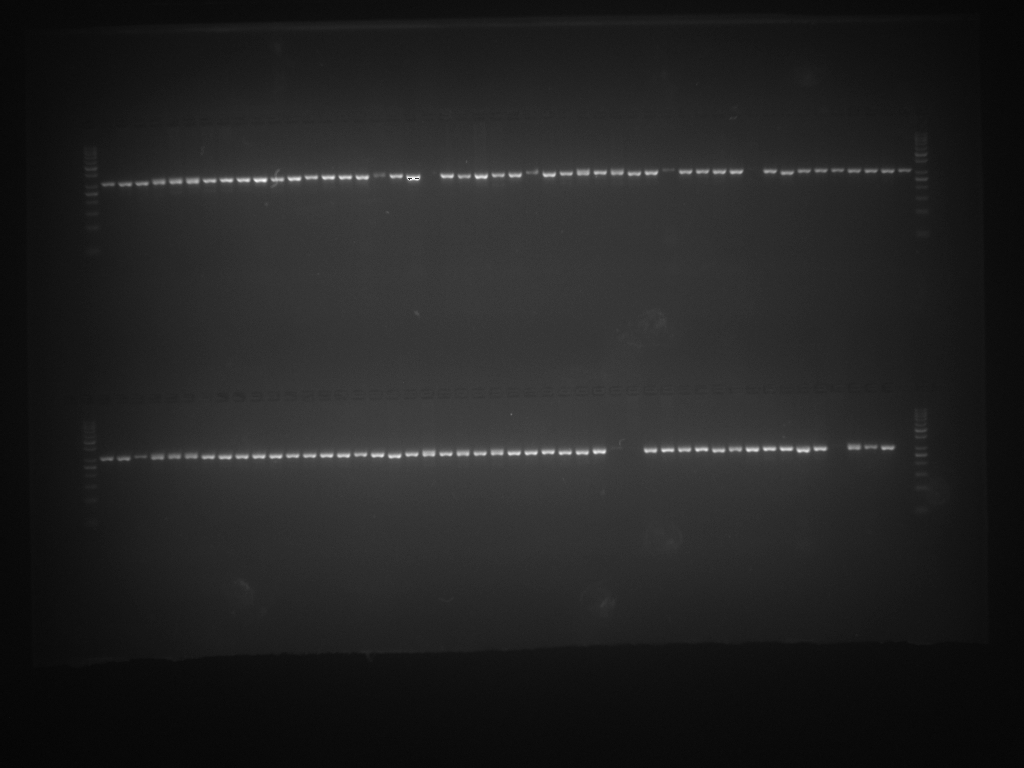

Supplement: S1 File — (ZIP) [file pone.0260246.s002.zip › MYB A09 21-2-2020.jpg]

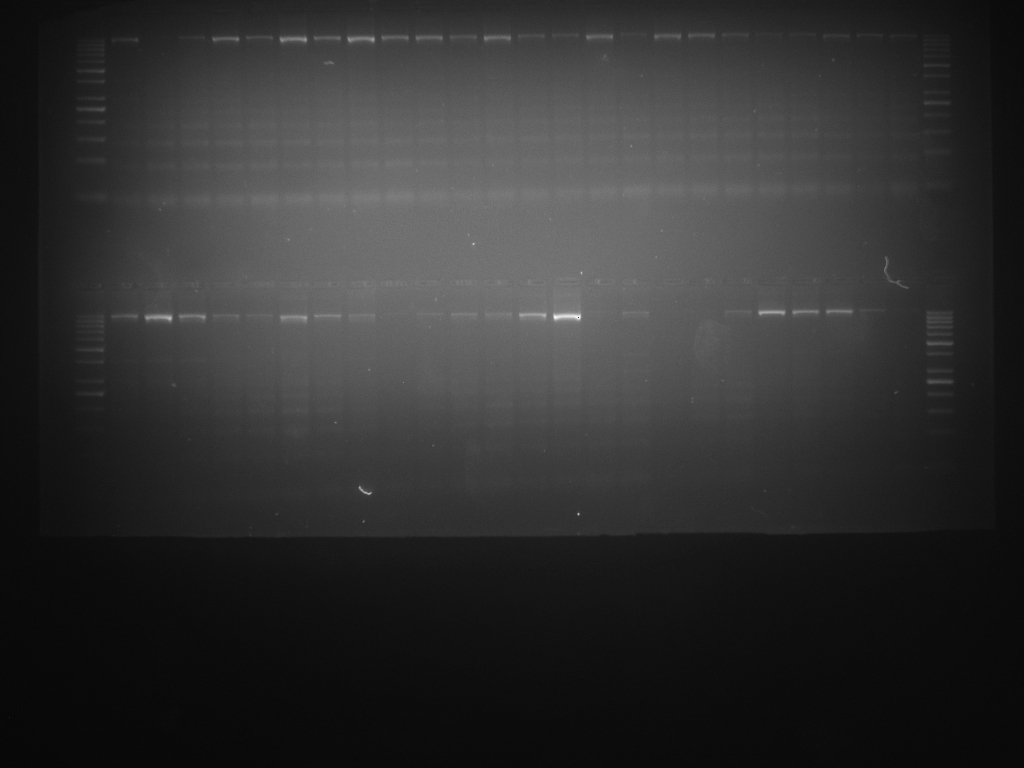

Supplement: S1 File — (ZIP) [file pone.0260246.s002.zip › MYB B 20-2-2020 LANE 3 AND 4.jpg]

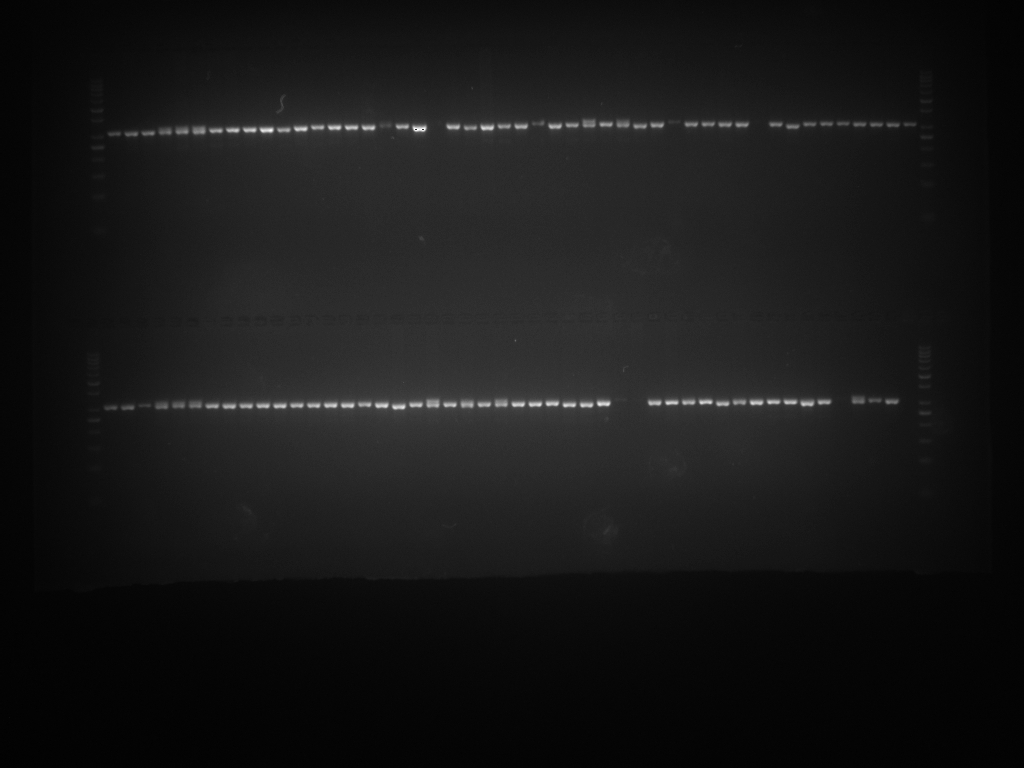

Supplement: S1 File — (ZIP) [file pone.0260246.s002.zip › MYB28A09 21-2-2020.jpg]

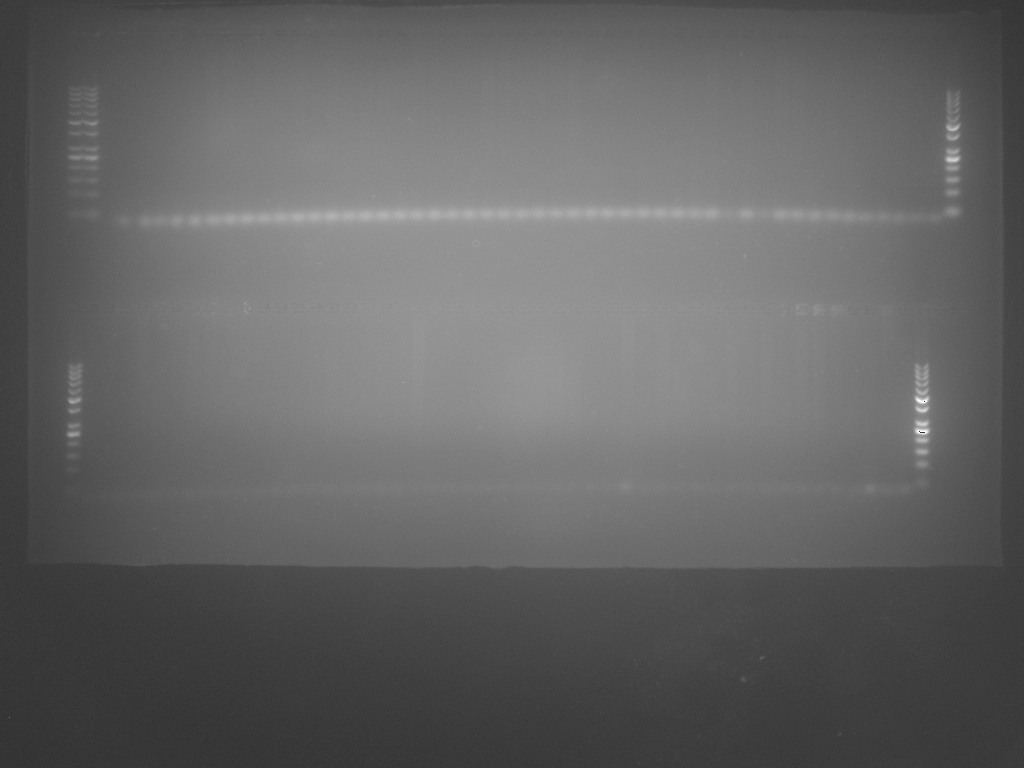

Supplement: S1 File — (ZIP) [file pone.0260246.s002.zip › NA10 B08 5-2-2020.jpg]

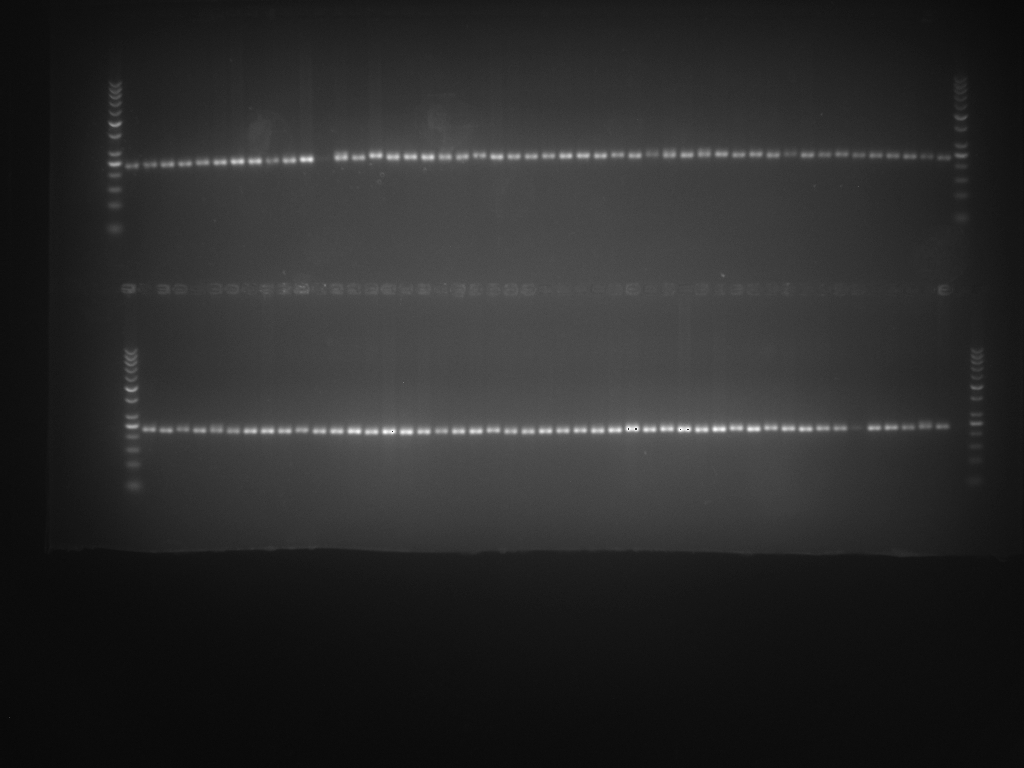

Supplement: S1 File — (ZIP) [file pone.0260246.s002.zip › NA10 G06 5-2-2020.jpg]

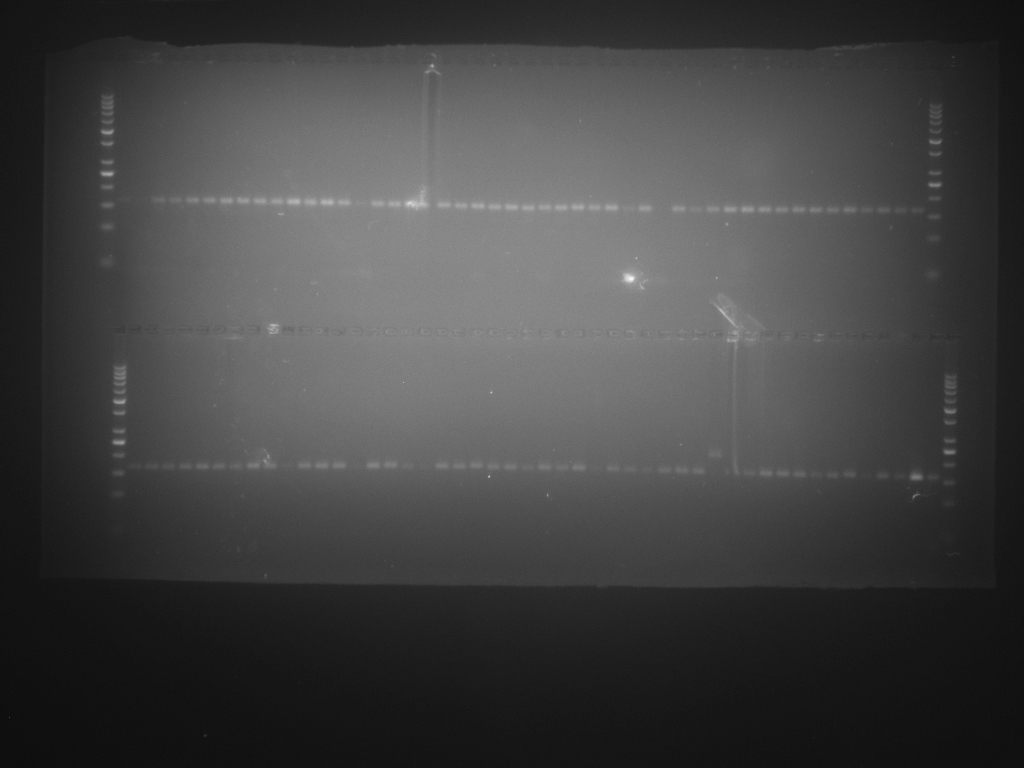

Supplement: S1 File — (ZIP) [file pone.0260246.s002.zip › NA10D11 LANE 3 AND 4 27-2-2020.jpg]

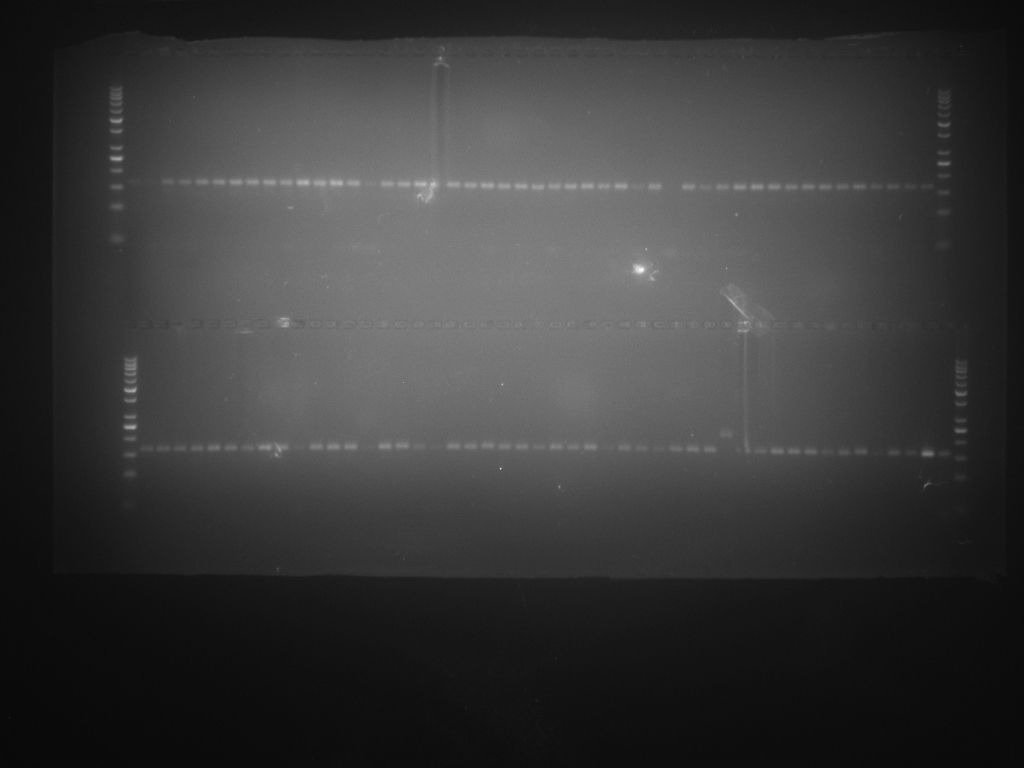

Supplement: S1 File — (ZIP) [file pone.0260246.s002.zip › NA10D11 27-2-2020.jpg]

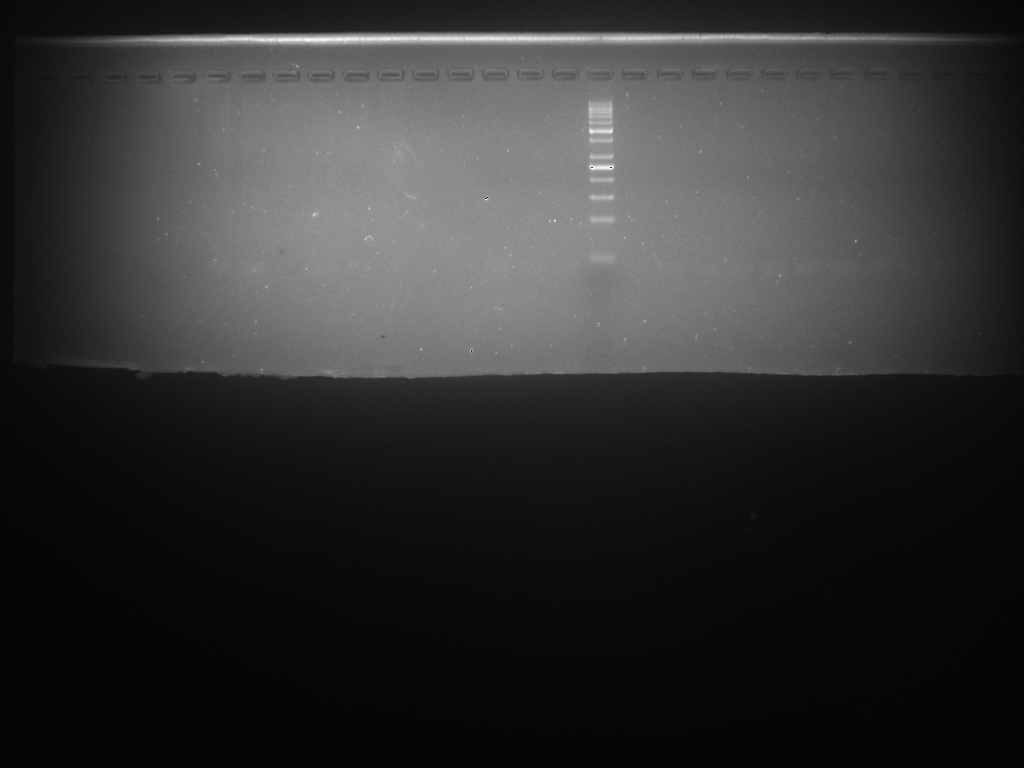

Supplement: S1 File — (ZIP) [file pone.0260246.s002.zip › NA12 G04 4-2-2020.jpg]

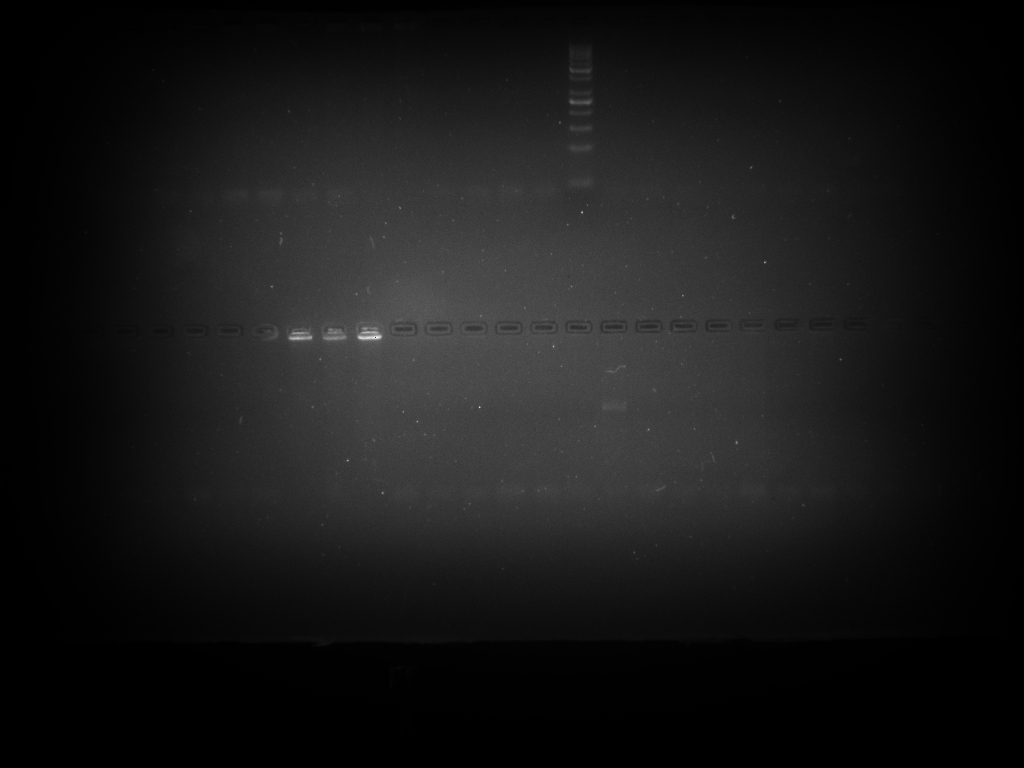

Supplement: S1 File — (ZIP) [file pone.0260246.s002.zip › NA12G04 LANE 2 AND 3 4-2-2020.jpg]

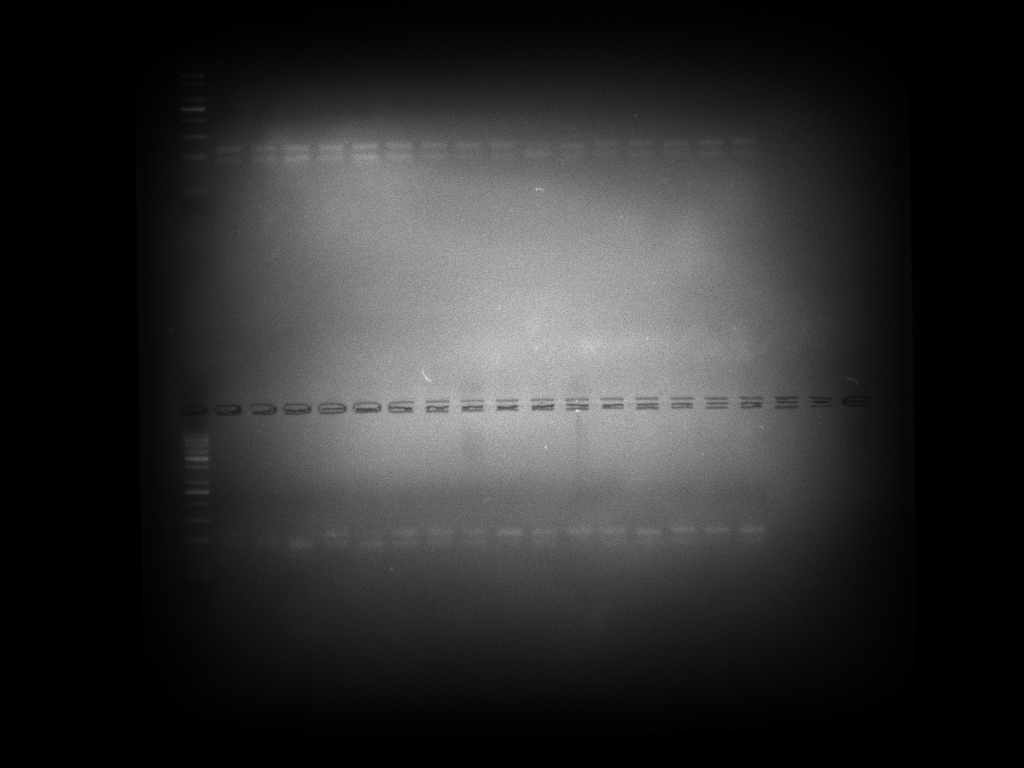

Supplement: S1 File — (ZIP) [file pone.0260246.s002.zip › NA14 E 11 4-2-2020.jpg]

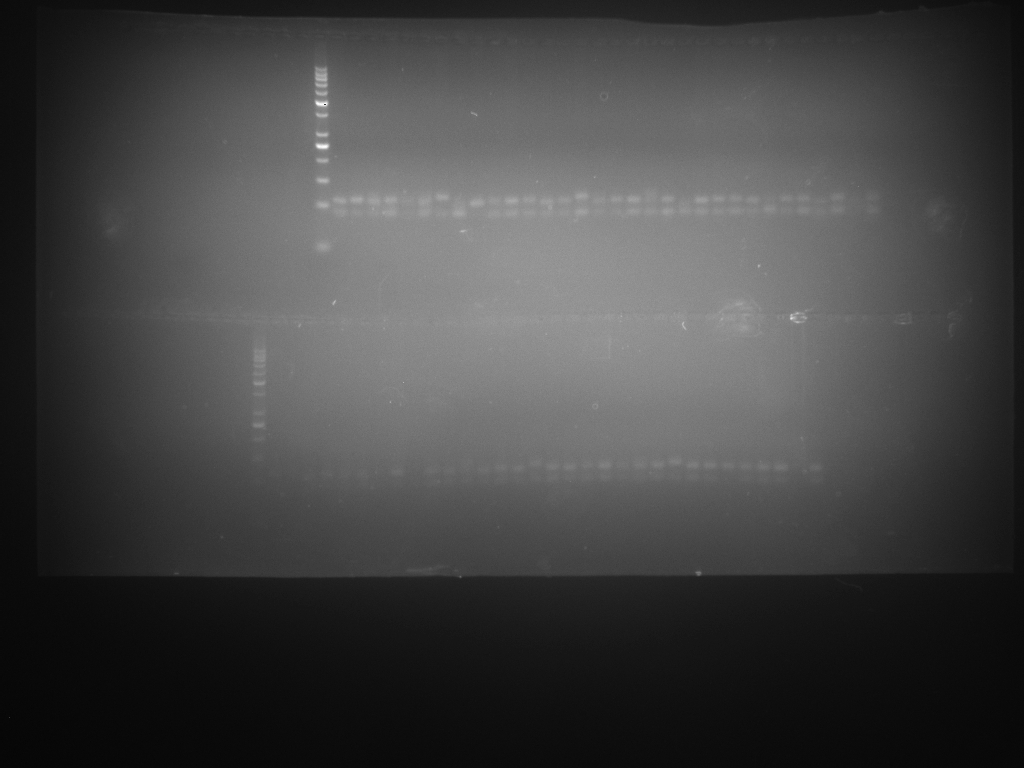

Supplement: S1 File — (ZIP) [file pone.0260246.s002.zip › NA14 E-11 4-2-2020.jpg]

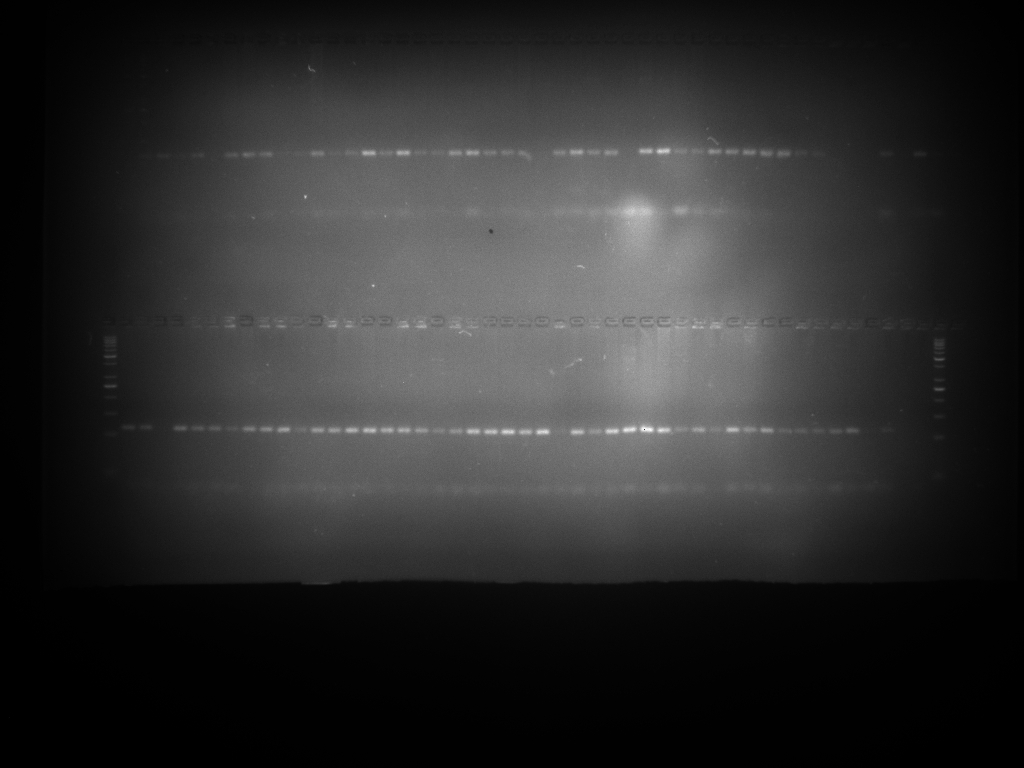

Supplement: S1 File — (ZIP) [file pone.0260246.s002.zip › Ni4B-06 4-2-2020 LANE 1 AND 2.jpg]

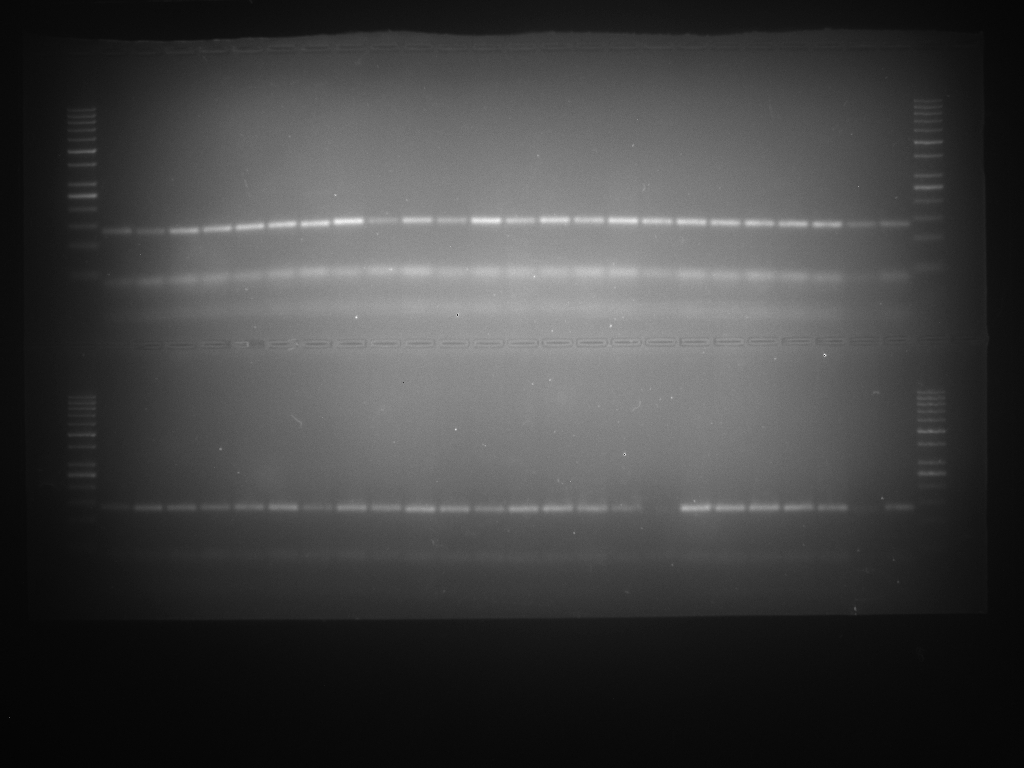

Supplement: S1 File — (ZIP) [file pone.0260246.s002.zip › O10B02 27-02-2020 ,,.jpg]

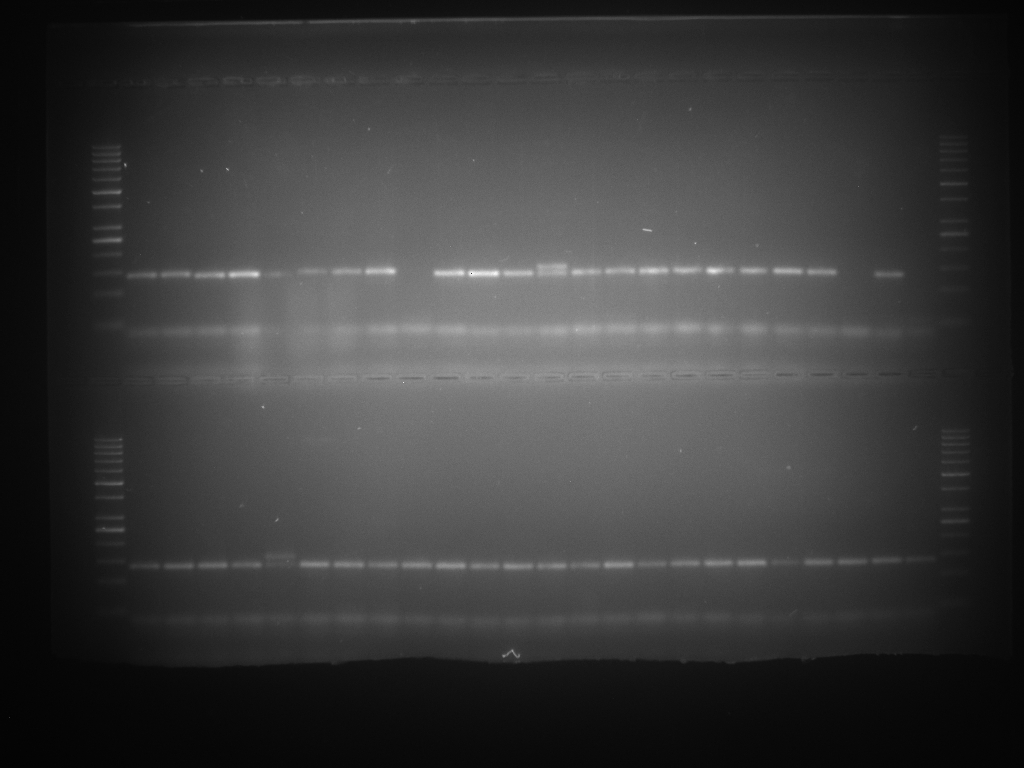

Supplement: S1 File — (ZIP) [file pone.0260246.s002.zip › O10B02 27-2-2020.jpg]

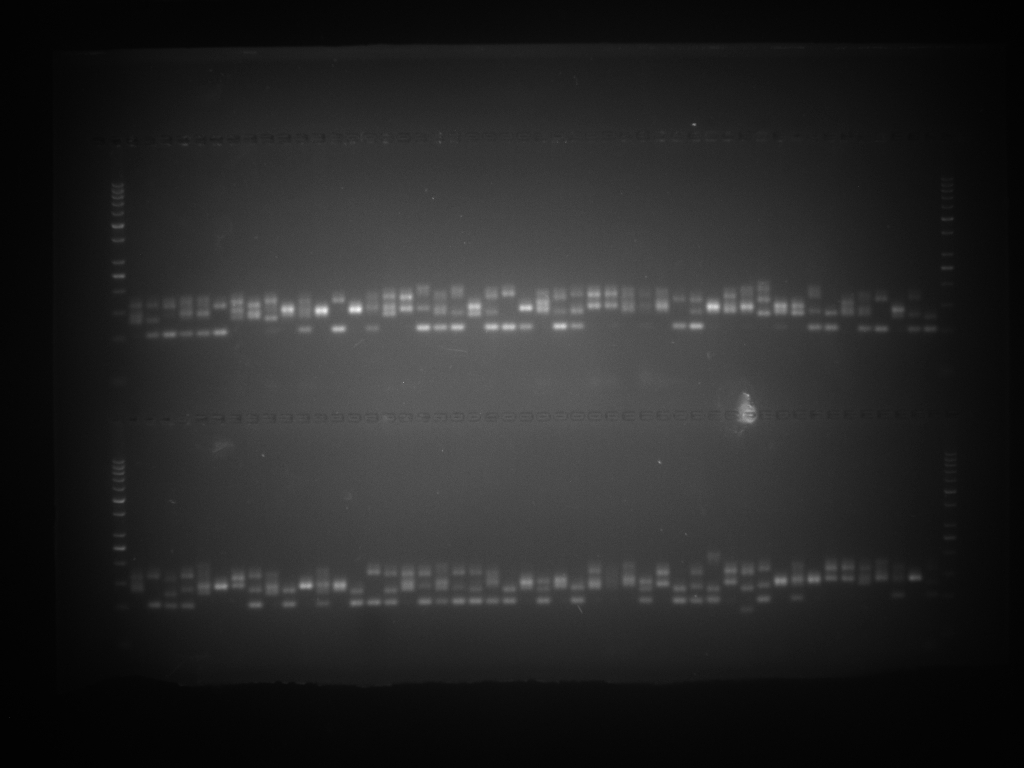

Supplement: S1 File — (ZIP) [file pone.0260246.s002.zip › OI 10D03 27-2-2020.jpg]

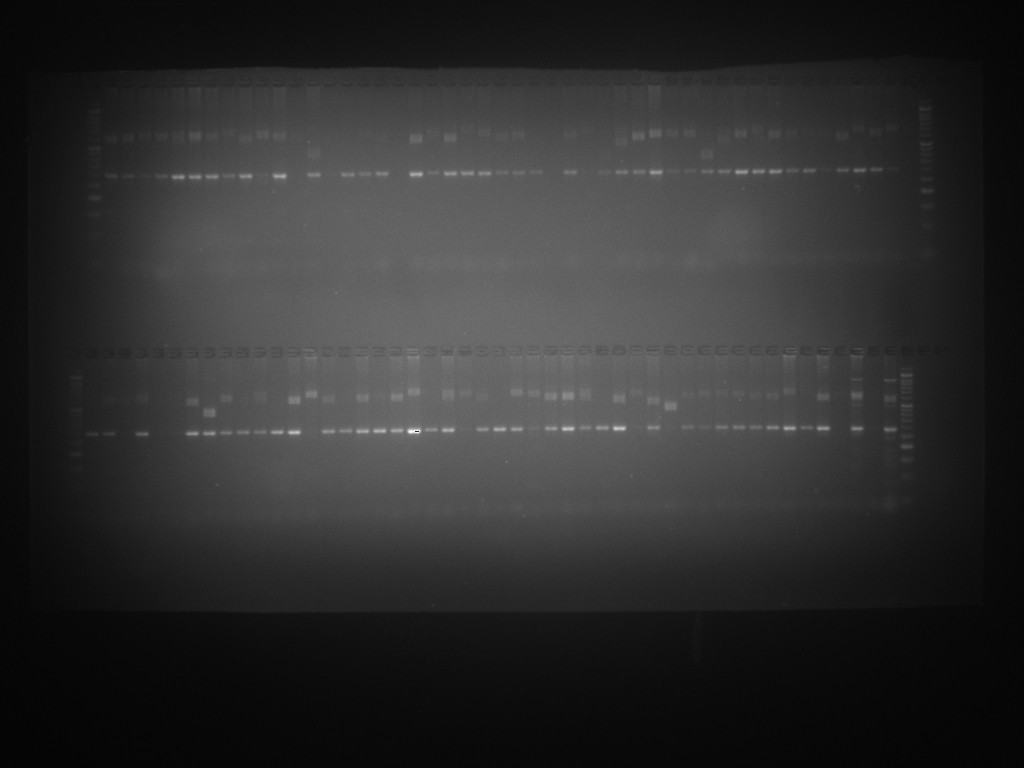

Supplement: S1 File — (ZIP) [file pone.0260246.s002.zip › OI13C12 1-3-2020.jpg]

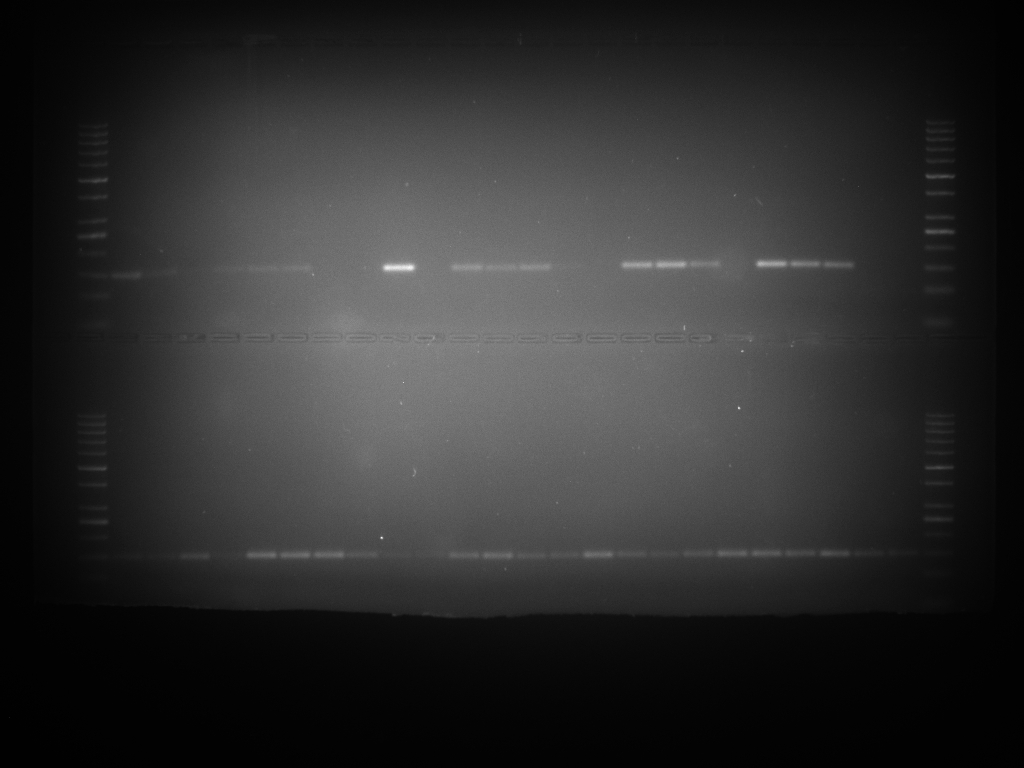

Supplement: S1 File — (ZIP) [file pone.0260246.s002.zip › OI0179 LANE 1,2 27-2-2020.jpg]
